# Major Greenwood (1880–1949): the biography

**DOI:** 10.1002/sim.7126

**Published:** 2016-09-22

**Authors:** Vern Farewell, Tony Johnson

**Affiliations:** ^1^ MRC Biostatistics Unit, Institute of Public HealthUniversity Forvie SiteRobinson WayCambridgeCB2 0SRU.K.; ^2^ MRC Clinical Trials Unit at UCLAviation House, 125 KingswayLondonWC2B 6NHU.K.

**Keywords:** Biography, Major Greenwood

## Abstract

Background is provided on the discovery of an unpublished biography of Major Greenwood written by one of his sons. The motivation and preparation for online publication of the biography in *Statistics in Medicine* are outlined. © 2016 The Authors. *Statistics in Medicine* Published by John Wiley & Sons Ltd.

## 1 Introduction

After completing a pair of papers that touched on the life of Major Greenwood 1, 2, we published in *Statistics in Medicine* 3 a study of his early career covering the period from his birth in 1880 to the end of the year 1910 when he left the London Hospital to join the Lister Institute. It was during the last part of this period that he established the first Department of Medical Statistics and gave the first lecture course on the subject.

Following the publication of this paper, Professor Peter Armitage highlighted the fact that although much had been written about Major Greenwood (3, Supporting Information, Appendix 2), there was no biography of him. While we shared his sentiment that such a biography would be welcome, we felt the task was beyond us for we could have only relied on his published works and the limited biographical material already available. In particular, we lacked significant knowledge of the personal details of his life, which would be essential to a full biography.

Nevertheless, in 2015, we did proceed to write a longer paper, which covered Greenwood's entire career from 1880 to his death in 1949, and this was also published in *Statistics in Medicine* 4. For both of our biographical papers 3, 4, we benefitted from comments from Dr Roger Major Greenwood who, like his grandfather, was qualified in medicine and trained as a statistician, and who also provided us with photographs from the family collection.

In a serendipitous conversation following publication of these two papers, Roger recalled that he had an unpublished‐typed manuscript of a biography of Major Greenwood written by his uncle, George Baur Greenwood (1912–1985). After locating it, he kindly made the manuscript available to us.

## 2 The original manuscript

### 2.1 Background

The author of the manuscript was the younger of Major Greenwood's two sons. Before and after World War II, George Greenwood held management positions in department store chains. During the war, he was a Labour Officer at the Royal Ordnance Factory in Chorley. He had a particular responsibility for compiling absenteeism statistics (it was a criminal offence to be absent without good reason when working at a Royal Ordnance Factory). Interestingly, his father dealt with absenteeism statistics from munitions factories during World War I when, as a Captain in the Royal Army Medical Corps, he was seconded to the Ministry of Munitions.

The original manuscript, dated 1984 and copyrighted to the author, was originally sent to us as .jpg images. To allow access to the text for editing, we re‐typed the manuscript in MSWord making only minor changes (such as spelling corrections). We then read the biography carefully and were impressed by its level of detail, quality and the extent of the personal information it yielded about Greenwood and some of his friends and colleagues. It also included references to some publications, especially newspaper articles by Greenwood of which we were unaware. We had no doubts that the material would be of interest to medical historians, epidemiologists and statisticians.

We were aware that the manuscript had been seen by Austin Bradford Hill because a letter from him to George Greenwood, dated 15 October 1984, was included with the original manuscript. Hill thanked the author for ‘letting me cast my eye over the account of your father's life again’, so clearly he had already viewed it, or some of it, previously. The reason for Hill's request was that the *British Medical Journal* was republishing *The Medical Dictator and Other Biographical Studies*, and Hill had been asked to write a Foreword of 2000–3000 words. This explains the personal detail regarding Major Greenwood that appeared in Hill's Foreword 5.

### 2.2 Second opinion from Professor Peter Armitage

To obtain a second opinion of the biography, we provided a confidential copy to Professor Peter Armitage who was acquainted with Major Greenwood at the London School of Hygiene and Tropical Medicine, and who had provided his personal reflections on Greenwood that formed a part of our second paper 4. His view was that it was ‘a remarkable find’, and he commented ‘how beautifully it is written’ and that it ‘covers the family background more fully’ than we had been able to do previously and ‘provides perhaps unique insight into Greenwood's relations with so many of the key players such as his father, Bacot, Karl Pearson and Yule’.

### 2.3 Re‐typed manuscript

We sent our re‐typed manuscript to Roger Greenwood asking for permission to investigate ways of publishing it. He circulated it to other members of the Greenwood family including Mrs Joyce Greenwood, the author's widow, her son, Mr John Major Greenwood, and daughter, Ms Diana Greenwood. They all agreed that the biography could be published but requested that this should essentially be as written by George Greenwood.

We have honoured their instructions and thank them all for allowing us to investigate and proceed to publication. The changes we have made are detailed in the succeeding discussion and have been made with the family's permission.

### 2.4 Changes to the original manuscript

We have instituted some minor formatting changes to reflect current standards. For example, book and journal titles are placed in italics instead of quotation marks, underlining is replaced by emboldened section headings or italicized text as appropriate, full stops between initials and individual capital letters in qualifications have been removed and two places where words have been omitted are indicated. We have also inserted main section headings (with dates) to demarcate the principal periods in Greenwood's life and placed the original sections as sub‐sections within these. We have also inserted two headings where there appeared to be a break in the narrative, expanded some references to identify the sources, integrated footnotes into the main text and inserted the full text of a poem mentioned in the text, Rudyard Kipling's *The Power of a Dog*, because, as is noted in the text, ‘a faded typescript copy’ of this was found in Greenwood's pocket book after his death and dogs were clearly a very important part of his life.

Finally, we added a list of contents and five photographs of Greenwood (one provided for 3, one additional photo from Roger Greenwood, one from the Wellcome Library and two from our earlier paper on Greenwood's colleague Hilda Woods 2).

### 2.5 Statistics in medicine and a family foreword

While editing the manuscript as described previously, we asked the Editors of *Statistics in Medicine* if they would consider publication in some form. We thank them greatly for their ready willingness to publish the biography online, along with this brief introductory article; we thank Editor Dr Simon Day for his comments on the manuscript that directed some of our final editing.

Major Greenwood's family kindly agreed to the inclusion of a foreword to the published version of the biography that was provided by Roger Greenwood.

## 3 Content and structure of the biography

Both Professor Peter Armitage and Dr Simon Day commented that, while the content of the Biography was very interesting, it left an impression of being ‘not quite complete’. This was also our impression on first reading and requires some comment because it does not have the structure of a ‘standard’ biography.

It is written primarily as a series of snapshots of Greenwood's career, organized predominantly in chronological sequence, with each snapshot having its own section. The account is factual with limited discussion and presented almost solely in the context of Greenwood's career without amplification of the broader scientific and political background. However, the biography portrays aspects of Greenwood's personal and family life not found elsewhere, as commented upon earlier.

The individual sections are reasonably detailed up to 1934, the year in which Greenwood started to keep a diary; at this point, the style changes and events of note are interspersed with extracts from the diary itself. As George Greenwood remarks, Major Greenwood was ‘no diarist at heart’ for he never recorded conversations. Indeed, the diary was mainly a listing of ‘his main appointments for the day, the things he did, people he saw and books he was reading’. The extracts nonetheless reveal some important details of Greenwood's life, and ‘for all their scantiness … are at times amusingly self‐revealing’.

One of the most surprising revelations is that Greenwood finished writing a book entitled *Epidemics of Body and Mind* that has never been published. Given Greenwood's association with Culpin, a major researcher in this field, it would be fascinating to gain some knowledge of Greenwood's ideas of epidemics of diseases of the mind, if a manuscript of the book could be located.

## 4 Envoi

Once again, we record our thanks to the Greenwood family for allowing us privileged access to this biography of Major Greenwood and in addition for allowing us to prepare it for publication. We thank the Editors of *Statistics in Medicine* for enabling its publication.

## Supporting information

Supporting info item
