# Supplementary material for: Major Greenwood (1880–1949): the biography
Source: Stat Med. 2016 Sep 22;35(30):5533–5. doi: 10.1002/sim.7126 (PMC5157783; doi:10.1002/sim.7126)
Supplement: Supplementary file 1 — Supporting info item [file SIM-35-5533-s001.pdf]

MAJOR GREENWOOD, F. R. S.

Pioneer of Medical Statistics.

George Baur Greenwood

[1912-1985]

Communicated by: Mrs. Joyce Greenwood 2016.

Contents*

Foreword by Dr Roger Major Greenwood

List of Photographs

1. Family History and the Early Years (1880 – 1900)
- **1.1. Forebears**
- **1.2. Early Years**
- **1.3. Sickness and Research**

2. The London Hospital, Marriage, and the Opsonic Index (1900 – 1910)
- **2.1. Karl Pearson**
- **2.2. Study Under Pearson**
- **2.3. Demonstrator in Physiology**
- **2.4. Opsonic Index Controversy**
- **2.5. Marriage**

3. The Lister Institute (1910 – 1914)
- **3.1. Appointment to the Lister Institute**
- **3.2. Indian Plague**
- **3.3. Opsonic Index Enquiry Continues**
- **3.4. Predisposition and Alcoholism**
- **3.5. Scientific Papers**
- **3.6. Charles Creighton**
- **3.7. Friction with Pearson**
- **3.8. Advancement**
- **3.9. Arthur Bacot at the Lister Institute**
- **3,10. Battling for the Faith**
- **3.11. Quarrel with Wright**
- **3.12. George Udny Yule**
- **3.13. Swine Fever**

3.14. Bawdiness and the Birth Rate Committee

3.15. Threat to the Lister Institute

4. First World War, Army Service, and Ministry of Munitions (1914 – 1918)

4.1. Outbreak of War

4.2. Army Service

4.3. Ministry of Munitions

4.4. Liquor Control

4.5. Labour Wastage

5. The Ministry of Health (1919 – 1927)

5.1. Post War Uncertainties

5.2. At the Ministry of Health

5.3. Reconciliation with Pearson

5.4. First Chairmanship and Leon Isserlis

5.5. *The Health of the Industrial Worker*

5.6. Death of Arthur Bacot

5.7. International Health

5.8. Major Edge

5.9. Foreign Affairs

5.10. The Hampstead Circle

5.11. Epidemiology of Mice

6. The London School of Hygiene and Tropical Medicine (1927 – 1945)

6.1. Foundation

6.2. The Royal Society

6.3. The Culpin Circle

6.4. The Academic Plateau

7. *“Hillcrest”*

7.1. His Home

7.2. The Study
7.3. His Books
7.4. Intruder
7.5. The Daily Round
7.6. Dogs and the Sunday Ritual
7.7. Christmas

8. Major Greenwood, The Man
8.1. Music
8.2. Appearance
8.3. Conversations
8.4. Reading and Working
8.5. Experts and Amateurs

9. Notable Events (1931 – 1939)
9.1. Visit to the United States
9.2. Academic Assistance Council
9.3. Karl Pearson and the Guy Medal in Gold
9.4. Diarist
9.5. Nutrition Controversy
9.6. Centenary of the Royal Statistical Society
9.7. President of the Society
9.8. Mice
9.9. *Epidemics and Crowd Diseases*
9.10. Fathers Lopez and Albert
9.11. The Senate
9.12. A Dream
9.13. Death of Karl Pearson
9.14. *The Medical Dictator*
9.15. Lord Kennet of the Dene
9.16. Convocation

9.17. Fit of Terror

9.18. Overwork

9.19. Winston Churchill

9.20. Bisset Hawkins Medal

9.21. In imitation of Alexander Pope

10. War Again (1938 – 1945)

10.1. War Scare

10.2. Outbreak of War

10.3. Diary Extracts: 1940

10.4. Air Raids

10.5. Diary Extracts: 1942

10.6. Diary Extracts: 1943

10.7. Fitzpatrick and Linacre Lectures

10.8. Diary Extracts: 1944

10.9. *Everybody's Political What's What*

10.10. The Guy Medal in Gold

10.11. "Victory in Europe Day" Thoughts

11. The Final Years (1945 – 1949)

11.1. Retirement

11.2. Diary Extracts: 1946

11.3. The Letter from Downing Street

11.4. The Communist Danger

11.5. Towards Three Score and Ten

11.6. Diary Extracts: 1949

*** As an aid to following the sequence of events we have inserted main headings to cover the principal periods in Greenwood's life including dates. The individual sections of the Biography are then presented as sub-sections as shown above. We have maintained the original order of the sub-sections.**

Foreword

MAJOR GREENWOOD, F.R.S. : Pioneer of Medical Statistics by George Greenwood

Major Greenwood had two sons by his wife Rosa, née Baur. The younger, George wrote this biography. The elder, John, was my father. George's widow Joyce is still alive in her 90's but has no recollections on the writing of Major Greenwood's biography. George was a prolific writer, county councillor, and wrote and edited newsletters for two local history societies. He also published a novel entitled *Misadventures of a Tookeyman* under the pseudonym Richard Millar, which was a fictionalised account of his life working in a large department store before and after World War II. According to his son John, the household was 'Edwardian' in that there was a 'wall' between George's study and the rest of the household.

When Major's wife Rosa died in 1945, my parents and I moved into Major's home, "*Hillcrest*" 102 Church Hill Loughton, so that my mother could keep house for him. We all lived there until 1949 when Major suddenly and unexpectedly died.

I can remember aspects of this period well, though some of my memories may be from earlier visits when my grandmother was alive. In 1949 I was 12 years old. So it was a long time ago. The four of us lived as a family. I can remember evening meals and Sunday lunches when we sat around the dining room table, my father opposite me, my grandfather to my left and my mother to my right. My main memory is of long arguments, especially after a meal, between my father and grandfather. Most of these passed over my head. One I remember concerned rocket propulsion in outer space. Grandfather firmly maintained that a rocket could not propel itself as the exhaust had nothing to push against. My father pointed out Newton's third law and V2 rockets. I am not sure that grandfather really believed what he argued, as the family members often deliberately took an opposite view just to have an argument. I was occasionally asked what I was doing at school and grandfather had a tendency to ask rather penetrating questions which I sometimes had difficulty in answering. In general I remember him with great affection as being a kindly man. His death was a great shock.

Grandfather slept in a large spare room which was away from the road and looked out over the terraced garden at the rear of "*Hillcrest*". My parents slept in the main bedroom on the road side of the house. My room was adjacent to my parents' room and connected to it via a door. It was the room used by my father when a child and it was directly above Grandfather's study. Being young I was sent to bed earlier than the rest of the family. I can vividly remember lying in bed with the sound of a Brunsviga mechanical calculator grinding away below me. It started quietly and gradually rose to a crescendo, stopped, paused and started all over again. This was a sound I became familiar with many decades later when summing squares while I was doing the "Short Course in Medical Statistics and Epidemiology" in 1966 at the London School of Hygiene and Tropical Medicine. What grandfather was actually doing all those years ago I have no idea. When grandfather died I thought I might be able to use this machine for homework, but the machine was the property of the London School of Hygiene and Tropical Medicine and they swiftly claimed it back. My father told me that it was an unusual machine as it had a transfer register, which I understood was a mechanical version of the storage register found in some electronic calculators.

Grandfather's study I tended to regard rather as the Holy of Holies. It was just inside the front door on the right. The study door opened into the corner of a room perhaps three by four metres. On the far left was a fireplace with a huge bottle of Stevens ink in the middle of the mantelpiece. The wall above was bare in contrast with all the remaining walls which were concealed by bookcases from floor to almost the ceiling, all absolutely full of books. In the middle of the far wall in front of a big window was grandfather's desk on which there was the calculating machine and a typewriter with various papers. *Hillcrest* had three floors. At the top were two bedrooms for servants and a large central room with long rooms off to either end which were under the eaves. (I had an air rifle range in one of these.) The main central room contained a table tennis table and, like the study, was lined with very full bookcases.

I can remember playing table tennis against my grandfather in this attic. I was running to

and fro at my end while he just stood still at his end batting the ball back with a very low probability of missing it. He also taught me to play chess and loaned me a book on chess and its openings which I still have. He almost invariably won, which did not bother me in the least. I judged my performance by how long I lasted before being checkmated. I remember trying out some of the openings described in the book he loaned me. I found that when I attempted openings noted as weak that I did not last as long as when I used other ones. I usually played a defensive game. Once I did go for an all-out attack and he resigned. I suspect out of surprise rather than due to any skill on my part. We also went for long walks together in Epping Forest.

Uncle George occasionally visited us. He was a man of great enthusiasms. He and his family lived on the opposite side of London. He too had a study containing many books and did much writing.

Grandfather had a great friend of many years standing who lived within 10 minutes walking distance, a Mr. Nello. They met regularly and played chess together.

During the war grandfather did some medical work. I can remember going with him to visit a local nursing home and sitting with him in matron's office while he discussed some matters with matron and signed several medical certificates. He also had a petrol ration because he was a medical doctor.

I was in awe of my grandfather as he was rather looked up to as a 'great man'. I knew he was a Professor of Statistics and Epidemiology. I had some idea of what epidemiology was about, but none, at that time, of what 'statistics' involved. Subsequently I learned more of the latter as I pursued my own career in medical computing and statistics and came to understand more why Major Greenwood was regarded as a great man. I am very pleased that this biography can now be made available to a wider audience.

Dr Roger Major Greenwood
East Leake, Nottinghamshire,
May 2016.

List of Photographs

Figure 1: Major Greenwood age 10 years with his mother, Annie Greenwood (née Burchell, 1858 – 1904) (provided by Roger Major Greenwood).

Figure 2: Major Greenwood between the ages of 20 and 30 years (1900 -1910) (provided by Roger Major Greenwood).

Figure 3: NPG x167957, Major Greenwood by Walter Stoneman,bromide print, 1931. © National Portrait Gallery, London.

Figure 4: Major Greenwood, relaxed and smiling (reproduced from Farewell, Johnson and Gear, *Journal of the Royal Statistical Society, series* A (2012); 175 (3): 799-811).

Figure 5: Major and Rosa Greenwood (edited from Farewell, Johnson and Gear, *Journal of the Royal Statistical Society, series* A (2012); 175 (3): 799-811).

1. Family History and the Early Years (1880 – 1900)

1.1. Forebears

Greenwood's forebears on his father's side have been traced back to the 1590's. They were small yeoman farmers who held copyhold land at Haddenham in Buckinghamshire. In 1682 a younger son came to London and was apprenticed to a silk weaver. His son, also a weaver, died in poor circumstances in 1732. His son James was educated at Christ's Hospital and apprenticed on charity money to become a Funeral Undertaker. He did well and founded a line of small shopkeepers in Bethnal Green. Greenwood's great grandfather, George, was a Smith & Bellows Maker. He and his wife both died in 1838 / 9 leaving two young boys, Major and James. They were cared for by relatives and both benefitted from the Will of a great uncle, James George, one of the Undertaker's sons. James George, who seems to have been some sort of property speculator, made a good deal of money which he left to his numerous relatives when he died in 1837. Although the portions of the two boys were initially quite modest, they were increased by the earlier deaths of other beneficiaries. With the money in hand, the boys' trustees apprenticed them to the medical profession. Greenwood's grandfather, Major the First, had six surviving sons. Four of them became doctors and two became lawyers. Greenwood's father and grandfather both practised medicine in the Dalston area of East London.

1.2. Early Years

"My family group," wrote Greenwood, is "interesting as an example of cockneyism. Both my grandfathers, my father and four uncles were general practitioners of medicine. All first worked in East London. I do not think my father and his father ever slept for more than thirty consecutive nights more than twenty miles away from St. Paul's Cathedral. I myself have never slept more than forty consecutive nights further away."

Greenwood was born at 2.30 pm on August 9th, 1880, at 18 Queen's Road, Dalston, where his father was sharing his father's medical practice, Greenwood Senior living at number twenty six.

He was christened Major, after his father and grandfather, a name which gave rise to irritations later in life.

According to a *Life History Album* his father kept, Greenwood was born prematurely, but he grew quickly and scarcely ever ailed. He was lucky to have survived at all. The other two children born to his parents died in infancy from a tubercular infection, which ultimately carried away his mother.

At five, his father noted: “His intellect is rather precocious. Learns rapidly and notices with great quickness, and remembers things said in his presence.”

After a year or two at a dame’s school in Hackney, run by a Miss Wright, in September 1889 he was sent to Merchant Taylors’ School where his father and several uncles had preceded him. For the period it was a very good school; the teaching was effective but unimaginative. Not a breath of modern air troubled the Classical side.

The pattern of education was simple. The general run of boys left school at sixteen or seventeen to go into business. Those who stayed aimed to go either to the Universities, which meant Oxford and Cambridge, or into medicine, which usually meant London University.

Greenwood wanted to study history and go to Oxford or Cambridge; his father was determined that he should study medicine and go to London.

His school career was erratic. Although he took about seven prizes, he lacked concentration. He was for ever wanting to study something that was not then his business; he took strong likes and dislikes for particular masters, and being at loggerheads with his father over the choice of a career was ever on the lookout for a way of escape.

Figure 1: Major Greenwood age 10 years with his mother, Annie Greenwood (née Burchell, 1858 – 1904) (provided by Roger Major Greenwood).

As a schoolboy he was a little, slight chap, perpetually amused by what went on around him, and by the comic side of pomposity. He translated his amusement into a flow of caricatures during which he deluged himself and his surroundings with ink. He was a leading spirit in what his form master called the Rowdy Gang.

He took little interest in athletics. His single recorded achievement was second place in a sack race in 1892!

Recollecting this period, Greenwood wrote; "Parents in narrow circumstances. As the only surviving child I was much in the company of older people, my parents and their friends. That probably encouraged priggishness, and had another long time effect; *viz*. that my intimate friends have mostly been men several years older than myself."

Neither of his parents was much of a home-maker. They were too busily pre-occupied with other things. Greenwood's father was an only moderately successful general practitioner in a crowded East End area rapidly running down in prosperity, and his heart was on other things. He was a cold man, socially untactful, not much given to small talk and bed-side chatter, self opinionated and a literary snob. But he was a laborious worker, widely read and a good scholar. As well as numerous minor medical appointments he became something of an expert on medical law, qualified as a barrister and secured the position of Deputy Coroner for North East London. He was full of divers interests ranging from the writing of novels (one of which was published though whether at his expense or the publisher's is not revealed), the composition of poems, the rendering of Chaucer's *Romaunt of the Rose* into modern English, historical articles on London, and long, exploratory cycle rides in distant parts of the kingdom to the urging of sanitary reforms and the improvement in the status of doctors backed by innumerable letters to the medical press. For all this he was a reserved, emotionally frigid man with some quirk in his character that kept back from the success that his intense industry ought to have merited. As a father he was man to be respected rather than loved.

Greenwood's mother was a little woman with dark piercing eyes, short hair, kind hearted but cynical, with a bitter tongue and the teller of tall stories, She was the daughter of old Dr Burchell to whom Greenwood's grandfather had been apprenticed. She had a first class brain, was very well educated, original minded, performed beautifully on the piano and spoke French with great fluency. She was well liked and Greenwood certainly loved her, but like his father, she was no home-maker. She led a rather lonely life and was

infected with a tubercular illness that carried her off when she was only 46. She was so "prickly" that it was difficult for new acquaintances to become intimate with her.

Greenwood's "home" had something of lodgings about it. He went there to sleep and do his homework, but seems to have escaped it as often as possible to his grandparents' house, where his grandmother made a great fuss of him. Indeed, his mother showed remarkably little concern whether he turned up for meals or not.

He left School in 1896, passed the university matriculation examination, and then attended Birkbeck College for eighteen months to study for the 1st MB. He was so idle over his proper work, and so industrious about other things, that to his father's indignation, he only passed part of the 1st MB.

One of Greenwood's preoccupations in the winter of '96 was Latin. It was an early instance of escapism, and for the next ten or fifteen years he was always trying to escape from situations. A first rate knowledge of the classics was the hallmark of the "Universities" – Oxford and Cambridge, but not London. He wanted to go to the "Universities"; his father decreed that he should study medicine at London. Whether he really thought of Latin as a kind of substitute for the "Universities" he has not recorded, but the love of it – and a relish for its peculiar distinction never left him. At 18 he was typing out "exercises" on his father's machine, and making spirited renderings of the Roman poets into English.

> Oh wither rush ye? Guilty band:
> Your new sheathed blades why seize again?
> Has not enough – on sea and land –
> Of Roman blood been spilt in Vain?
>
> (Plautus *Trinummis* (The Three Coins))

At 38 he was taking the Latin classics in their original away for holiday reading. At 58, in an age, when the classics had become unfashionable, his facility for quoting Latin tags

seemed to mark him out as a survival from an earlier age of learning. In his earlier struggles in the academic world, where the "Universities" were the accepted background, he made lavish use of Latin quotes as a substitute for the classical background he had never had.

In 1898 he was entered at the London Hospital and won an entrance scholarship in "arts", really elementary mathematics and Latin. According to his own account, for the first year he was extremely idle, and did no more than complete his first MB.

His entry coincided with a turning point in the affairs of the Hospital. Hitherto most of the teaching had been done by the hospital physicians and surgeons. However good these gentlemen may have been at their particular professions, they were not always good teachers.

A new system was introduced to the Medical School. Full time teachers were appointed to replace the somewhat casual activities of the hospital physicians and surgeons. One of the first of these appointments was Leonard Hill, who became lecturer in Physiology in 1895. Towards Hill, as he confessed afterwards, Greenwood developed an almost school girl passion.

Leonard Erskine Hill (1866 – 1952) came from a family of distinction. Many of his forebears had distinguished themselves in public and academic works; one of his great uncles had been Rowland Hill, the postal reformer, and his father was George Birkbeck Hill (1835 – 1903), the editor of Boswell's *Life of Johnson*. He had been appointed to the London Hospital teaching staffs in 1895, and when Greenwood first encountered him he was deeply involved in original research.

Greenwood confided to Hill that he disliked the prospect of becoming a General Practitioner, and was attracted to research work. Hill sized up the situation quickly. Since his father insisted, he advised Greenwood to get his medical qualification as quickly as possible, and not bother about degrees or prizes. Then, if he still had a mind to research,

to come back to him. Hill had a talent for spotting bright pupils and a sturdy goodness of heart to help them along. The interview had important results. Hill had befriended him, and Greenwood hitched his star to Hill's. The link was never broken, and until Greenwood was well on his way to success, Hill's helping hand was never far away.

Meanwhile a new influence entered Greenwood's life; Arthur William Bacot (1866 – 1922). In later years those who never knew their background wondered at Greenwood's fierce emotional regard for Bacot. He who was so emotionally reserved about most things, would allow his voice to falter and tears to start up in his eyes when the name of Bacot was mentioned in the years after his friend's death. And when Bacot was gone he would start a Memorial Evening to him, and week after week trace out the walks they had taken together in Epping Forest.

Bacot was a kindly misfit. Of Huguenot descent, as a child he had been "delicate" and his education neglected. At 16 he became a clerk in a city firm of tea workers and was still there when Greenwood met him. At 33 he was a bachelor who lived with his sister and aunt at Bow House, High Street, Lower Clapton. He hated clerking and spent his spare time reading widely and studying entomology.

About 1895 Bacot joined the North London Natural History Society. This had developed in association with the Science Club of the Grocer's School at Hackney. One of Greenwood's fellow students at the Hospital was Millais Culpin, an old Grocer's School boy and member of the Society. His parents had gone to Australia. He had no home in London, and went to stay with Bacot. In 1899 Culpin introduced Greenwood to Bacot, and they all became fast friends. Instead of going home in the evening, Greenwood used to slip round to Bacot's house, and there the three of them would work snugly together, Bacot at his entomology, the medical students at their books.

The Bacot establishment became a more real home to Greenwood than his parents' house. The Bacots possessed all the warm understanding qualities that his parents lacked.

Physically Bacot was not unlike Greenwood's father. In other respects he was an idealised reverse. In contrast to Greenwood senior's straight laced orthodoxy, his cold, forbidding nature and his self opinionated impatience, Bacot was easy going, warm hearted, and tolerant of most things. Greenwood senior was a conformist, a Lesson-reading church-goer, and a stickler for the accepted mode of behaviour in his social class. Bacot cared little for these things. He was an outsider, politically a radical. Compared with Greenwood senior he was a Bohemian, who wore odd clothes, had freakish taste in food, and scandalised the pious by collecting horse manure for his garden in a noisy old wheelbarrow on Sunday mornings. He was a fanatical hater of property owners, perhaps the only subject on which he allowed himself to be worked into a temper.

He was a left wing Fabian, and Greenwood, having never thought very much about political matters, quickly accepted his socialist views. It was an instance of emotional conversion, To the day of his death Greenwood remained a socialist, more in memory of Arthur Bacot, perhaps, than from real conviction.

Bacot's sister Alice was one of those jolly people who become the best loved universal aunt to all their acquaintances' children. She was an amateur artist and added to the family income by giving lessons in drawing and painting.

Bacot and Greenwood were in the same quandary. Neither of them liked the path chance seemed to have laid down for them. Bacot hated clerking, and Greenwood hated the prospect of becoming a doctor. But there was a difference. Bacot knew what he wanted to do, Greenwood didn't. He lived in a state of doubt and perplexity. Then chance intervened, and suddenly the doubt and perplexity were cleared away.

1.3. Sickness and Research

Early in 1900 Greenwood began to suffer from headaches. Two of the most distinguished specialists of the day were consulted, Hughlings Jackson and Victor Horsley. They diagnosed the ailment as having epileptic origins, and gave instructions for him to stop all

work for examinations for a year. As Jackson was an expert on epilepsy, and Horsley had made a special study of the localised functions of the brain, their diagnosis must have been extremely distressing. Yet nothing in Greenwood's previous medical history, nor anything afterwards, gives support to their views. After a period of rest he recovered and his health remained extraordinarily good. Looking back on the incident Greenwood liked to believe that his trouble had been psychoneurotic.

So as not to lose the year wholly from his medical studies, the experts allowed him to work in Hill's laboratory. Hill gave him a minor piece of research to occupy his time. This was an investigation into binaural effects. Hill was methodising what was then known about the special senses. The finer mechanics of the inner ear were not fully understood. One of the problems was the way in which the two ears synchronised sounds received from different angles and different distances. At the time Hill and Greenwood believed that their investigations were original, but unknown to them Silvanus Thompson had recently investigated the same subject.

The time in Hill's laboratory passed very happily, and confirmed Greenwood in his wish to escape from general practice into research. What this research was to be and whether any opportunities would present themselves remained as obscure as ever. But there was one straw in the wind of his experience that moved in a particular direction. It was the phenomena connected with heredity. It cropped up in several ways. His father, for instance, had been interested in heredity. When Francis Galton, inspired to study hereditary traits, urged the collection of biometrical data, Greenwood senior, had purchased several of Galton's "Anthropometrical Registers" with a view to making such records about his children. Thus Greenwood himself, as a child, had been subjected to measurements of one kind or another, and later on his father had explained the theory behind the scheme. Then there was Bacot's preoccupation on the hereditary characteristics of moths. This was Bacot's special study. He bred colonies of moths and studied the transmission of wing markings. Then again the medical world, and this included his father, grandfather and most of his uncles, who were continually bickering about the predisposition of particular people to develop particular diseases. A hot point in

this debate concerned tuberculosis. That it could be transmitted from person to person nobody seriously doubted, but the abounding mystery was why some people developed it. Was this predisposition, if it existed, transmitted from parents to their children, or could it develop independently?

There was another subject too, about which he heard a good deal, and which indirectly had a bearing on the same thing. The efficiency of vaccination. Both his father and grandfather were Public Vaccinators. Vaccination against smallpox was compulsory, but there were still many people, including members of the profession, who doubted its wisdom or its efficacy. The anti-vaccinators argued, among other things, that vaccination spread the disease, or alternatively, that those who were vaccinated and did not catch the disease would never have caught it anyway. It was perhaps a matter of predisposition. The only way to settle such disputes was by way of reference to the statistics of those who had or had not been vaccinated, and those who did or did not catch the disease. But the pro-vaccinators always found that their opponents could fault such references. Surely, they cried in distress, it ought to be possible to measure the success or failure of vaccination in a way that nobody can dispute. In a pamphlet on vaccination he wrote in 1886, Greenwood Senior had observed, "The proper understanding of statistics is only possible to experts, and the value they possess is obviously dependant on the accuracy with which they have been compiled, as well as on the extent and character of the data on which they rest."

At the time there were no experts in this field of research but the subject was not altogether neglected. A certain Karl Pearson, Professor of Applied Mathematics at University College, London, was just beginning to take an interest in the matter. In 1900, during his period with Hill, Greenwood discovered the works of Pearson. Suddenly all his doubts were resolved, and he knew the kind of research he wished to do.

Recalling how he had first read Pearson's *Grammar of Science* and been thrilled by its vista of measuring living things, Greenwood wrote, "In 1901 I went back to ordinary work, and then the enthusiasm excited by the *Grammar of Science* caused me to spend a

good deal of time extracting records of weights of viscera from the Post Mortem Room. I knew very little about biometry and in fear and trembling wrote to Karl Pearson, who invited me to come and see him in 1902. From that interview until now I have been a biometrician."

To quote Lancelot Hogben, Greenwood emerged from that interview "a convert to the new cult of biometry, a self dedicated pupil of the great man and anointed evangelist of the gospel of numbers." (Hogben L. Major Greenwood 1880-1949. *Obituary Notices of Fellows of the Royal Society* 1950; **7**:138-154).

2. The London Hospital, Marriage, and the Opsonic Index (1900 – 1910)

2.1. Karl Pearson

In 1901 Pearson and the biometricians stood for a new, exciting and controversial science. Biometry was a by-product of Darwinism. Francis Galton (1822 – 1911) started it as Anthropometry, the measurement of Man, with a view to tracing to exact laws of natural selection and inheritance. "Until the phenomena of any branch of knowledge have been submitted to measurement and number," he believed, "it cannot assume the status and dignity of a science."

Pearson (1857 – 1936) was the pioneer of the mathematical measuring processes. He not only did his best to infuse the spirit of mathematics into all biological sciences, but succeeded in erecting the very processes of measurement of all living things. It was the foundation course of modern mathematical statistics.

At his very simplest, Pearson showed that the old fashioned methods of simple counting were insufficient when applied as measurement to living organisms that were subject to the chances and changes of environment. Without making allowances for chance and change, it was dangerous to draw general deductions from biological data. Pearson was primarily searching for the laws of evolution, but his methods and pronouncements frequently brought him into conflict with established "expert opinion".

By the year 1901 he had assembled a biometric laboratory at University College, where he ran post-graduate courses in biometry, and conducted a great deal of ingenious research work. His teaching was beginning to show results. Here and there were people beginning to use his methods, and one of his assistants, George Udny Yule, who had recently left him, was lecturing on the subject on his own.

Greenwood’s first contact with the Pearsonian canon was the *Grammar of Science*. This was a massive work first published in 1892. It was intended to present to its readers in simple language the fundamental concepts of contemporary science. Pearson showed that the scientific method was essentially descriptive according to the sense perceptions, and that the scientific workers aim should be to find out HOW phenomena occurred, not WHY. Since the original publication date Pearson had become interested in evolutionary theory, and the second edition of 1900, which Greenwood read with so much enthusiasm, contained two additional and very long chapters on this subject.

There was something exhilarating about the book. It had a new slant on science. Pearson was a free thinker, a reformer and a socialist. He stressed the social value of science and made researchers into agents for the social good. He had a way of attacking old authorities that appealed to young men who longed to throw stones at old authorities. He gave the impression that mankind was on the edge of the most tremendous discoveries, and that the scientific worker could open up new realms of human happiness.

From the *Grammar of Science* Greenwood passed to other writings of Pearson’s and thrilled in sympathy with some of his opinions. One of these strikes a familiar note in Greenwood’s life-long aversion to “experts”:

“Religion once tyrannised the world, Science has followed Religion, but instead of setting up a republic of thought, has instituted a worse tyranny in its place, the oligarchy of scientific specialists, who expect mankind at large to accept on the ground of authority whatever they choose to proclaim as truth.”

However much he may have been influenced by the *Grammar of Science*, the work of Pearson's that directly activated Greenwood into doing something about biometry was the collection of essays entitled, *The Chances of Death and other Studies in Evolution*. This was a very odd collection of essays, some of which had previously been published in the *Fortnightly Review*. They included such items as, *Socialism and Natural Selection*, *Politics and Science*, *Reaction*, *Women and Labour*, *Variation in Man and Woman*.

The essay on *Variation in Man and Woman* has such a bearing on Greenwood's biometrical career that it is worth considering at some length.

The purpose of the essay was quite simple, and illustrates Pearson's biometrical methods. Havelock Ellis in his book *Man and Woman* had confidently asserted that:

(1) There was much greater variability in the male than the female type.
(2) That this variability had been one of the effective causes in determining the drift of civilisation.
(3) That this variability had had the widest social and practical consequences.

"The object of this essay," says Pearson in his introductory remarks, "is to lay the axe to the root of this pseudo-scientific superstition." And lay the axe he did, with all the biometrical precision he could muster. His method was simply to compare various measurable male and female data, such as bone lengths, and show that there was no evidence of greater variability in the male than the female range of measurements. After 119 pages of discussion and biometrical tables, he considered Ellis's assertions quite unproven and concluded with a remark characteristic of Pearson's brand of wit. Regretting that many writers on evolution had copied Ellis's assertions as facts, he commented, "The "sequacity" exhibited by the multitude of semi-scientific writers on evolution is possibly a sign of the very small capacity for intellectual variation possessed by the literary male."

Now the data at Pearson's disposal for his male and female comparisons were far from being as extensive or reliable as he would have wished, and there was a constant longing for more. Skulls were hardly the things one could collect in hundreds, much less livers and kidneys and other perishable data. The lay reader, in fact, looking over this work half a century later, is a little surprised to find so much mathematical precision applied to data of such questionable origin. But it was the best Pearson could get, and he was not a man to be daunted by difficulties. Thus we find him using the measurable bones of Ancient Egyptians, Romans, pre-historic Britons, and 17th century Londoners excavated from a cemetery at Whitechapel.

One of his sections dealt with the weights of male and female organs, hearts, livers, kidneys and so forth. For these he had been obliged to rely on post mortem records published as early as 1838, 1843 and 1846.

It was at this point that Greenwood saw his opportunity. He had at his disposal the records of the London Hospital Post Mortem Room. Indeed, the great Osler, whose *Principles and Practise of Medicine* he was then studying, continually urged the use of hospital records for research purposes. He decided to do some biometrical research himself. He began investigating the weights of viscera in the Hospital records. He also began to view Bacot's moth breeding experiments in a new light. If Pearson had been able to use biometrical methods for comparing the variability of men and women, could not similar methods be applied to measuring the variability in the wing markings of Bacot's moths?

By March 18th 1902 Greenwood had become so engrossed in these matters that "in fear and trembling", as he says, he wrote to Pearson, explained what he was doing and asked if the work could be of any scientific interest.

Pearson replied at once and invited him to call. He thought it possible that Greenwood's work on moths might be useful for *Biometrika* if he had not yet promised it elsewhere. *Biometrika* was a learned journal founded by Pearson in 1901 as a result of the Royal

Society showing reluctance to publish some of his papers in its *Philosophical Transactions.*

Greenwood agreed to call on Pearson at his rooms at University College at 4 pm, on the following Monday. The moment of conversion had arrived.

Pearson was a tall man, with a powerfully chiselled face, pugnacious jaw, high forehead and receding, backswept hair. He had something of the fanatic in his make up. He was pungent in controversy, and as he grew older, became intolerant and overbearing. He refused to suffer fools at all. His energy and working capacity were prodigious. He took great pains with his pupils, and inspired in many of them a personal devotion that survived the quarrels that his stormy temperament was apt to engender. In his biometric battles, especially when debunking “experts”, he made many enemies, but few of those who had most reason to dislike him, ever failed to respect him. He was the “terrible KP”, the ridiculer of the pontifications of the pundits, the layman who dared to attack medical opinion. He had just fallen foul of another *enfant terrible* of criticism, George Bernard Shaw.

What the Great Man said to Greenwood has not been recorded, but the sum was interest and encouragement. Greenwood left with him his notes on Bacot’s moths, and embarked on the laborious transcription of the viscera records.

At the end of March 1902 Pearson wrote that although there was much interest in the moth data, he was doubtful how it could be worked into mathematical form. For measuring hereditary characteristics one needed a large number of pairs of parents whereas Bacot’s data was based on a large number of offspring from few parents. It was, moreover, important that the offspring should have been born and reared in the same environment.

Thus advised and encouraged Greenwood redoubled his biometric efforts and hinted to his father of a desire to study under Pearson. His father, however, viewed these

biometrical excursions with misgivings and banned any possibility of studying under Pearson until he had passed his medical finals. Nevertheless progress was made with the viscera data, and the results, heavily revised by the Master, were eventually published in *Biometrika* in 1904. The moth data appears to have been beyond biometric treatment.

In 1904 Greenwood qualified MRCS, LRCP, and when he heard the results, treated himself to a slap-up meal at the "Rotunda". "Then", he recorded, "my father allowed me to study under KP provided I helped him in his practice. It was a very strenuous year. The demands of the practice were not heavy, still there they were."

In July 1904 he sounded Pearson on the prospects of biometrical methods being applied to medicine and Public Health, and wondered if he could take up part time study under him. On July 17th Pearson replied "I certainly think you might achieve a very great deal by giving the afternoons for some months to biometry, and there is a deal to be done in medicine and also in public health in the matter. Some day we shall doubtless have a Registrar General who knows something of statistics …"

Pearson agreed that Greenwood could join his study group in the autumn. As it was holiday time, Greenwood, full of zeal, wondered if he should go across to Germany and learn the language as the Germans were contributing much towards science. Pearson, however, was not interested in the contributions of the Germans, none of whom, he said, were doing much worthwhile biometric work. A knowledge of German would certainly be useful, and a seminar under Schwalbe at Strasbourg would teach him a lot. Pearson was remarkably insular in his biometric interests and could rarely be persuaded to pay much attention to the work of foreigners.

2.2. Study Under Pearson

For the next few months Greenwood helped his father and his medical relatives with their general practices. But he had no interest in clinical medicine, or in people. His bedside manner was terrible. He lacked the flow of soothing chatter so essential to make his

patients and himself feel at ease. He had no idea how to tackle the ordinary East Ender. His uncle's dispenser overheard the following exchange:

Patient: "Oh, doctor, shall I rub camphorated oil on my baby's chest?"

Greenwood: "If you want to do so, certainly do it. To me it has no value – except perhaps a pleasant odour."

Patient: "Well, anyway, Dr Arthur Greenwood told me it was good."

"General practice," said his aunt, "simply was not his line – the line when you give the patient something to do to make him feel you are interested."

As the date for starting his part time study with Pearson drew near, Greenwood was filled with worrying doubts. His father was still hostile in principle. Greenwood had a living to earn. Was it really wise to side track the main issue of general practice with fanciful notions of scientific research. Could one really earn a living by research? On September 21st 1904 Greenwood addressed his ambitions and doubts to Pearson.

Pearson's reply was long and painstaking. He agreed with many of Greenwood's views, and said that generally speaking, it was impracticable to take up pure research unless one had private means. On the other hand, there were people whose sheer determination overcame all obstacles. Not knowing Greenwood's capabilities, he was unable to advise him directly, least of all to recommend that he threw up medicine and devoted himself full time to biometry. On the other hand, if Greenwood studied under him for a time, he would give him the best grounding he could, after which the future would depend on Greenwood's own ability and determination. So far there were no biometrical posts and it might be a long time before any were established. Whatever his capabilities, a career in biometry would certainly be a slow and hazardous undertaking. He ended, "I wish I could give you better and clearer advice, but the answer must lie ultimately with yourself, for it

is bound up with the strength of will and power of endurance, which even an intimate friend could hardly estimate."

In October Greenwood started his training under Pearson and his "conversion" was complete.

As biometric techniques are based on mathematics, it is worth questioning how good a mathematician was Greenwood. Referring to these early days, he once wrote, "My mainly self taught mathematics were below the level of a pass BSc, but, with the help of a young Cambridge man who was KP's assistant – he boarded in my father's house – I did make progress." The Cambridge man was J Blakeman of Trinity.

Although Greenwood never claimed to be a mathematician, he had a distinct "flair" for the subject. Dr Leon Isserlis, who was a trained mathematician and whose help Greenwood sought, and with whom he sometimes collaborated in later years, said he liked mathematics, particularly algebra, for its own sake, and only used it for applications to statistics in later life. "For instance," says Isserlis, referring to the early years, "he read Netto's *Substitution Theory* – a book too difficult for 6th form schoolboys – and read MacMahon's *Combinatory Analysis* in later years, long before the Fisher school of Mathematical Statisticians appreciated its importance to their subject. In later years, when he wanted my cooperation in putting some statistical problem into mathematical form, he always knew the right questions to ask, and could follow the solution with ease … His statistical sense usually saw almost intuitively what the answer should be, and mathematical analysis usually showed he was right."

One of the first techniques that Greenwood learned from Pearson was the coefficient of correlation. In layman's terms it was a mathematical way of testing whether in a series of observations particular factors were related. It had been devised by a French mathematician, August Bravais, in the 1840s. Galton had been the first to apply it to biometrical observations but Pearson had greatly improved its application. He had done so with matters of evolution in mind. Greenwood was quick to see the value of its

application to medical problems. The medical world was full of self styled "experts" whose inferences varied according to the way in which they interpreted their data. Here, Greenwood thought, was an unbiased, scientific way of testing these inferences. The Bravais-Pearson coefficient was to become an extremely useful tool.

2.3. Demonstrator in Physiology

For the greater part of a year Greenwood enthusiastically part-time studied under Pearson, and drudged reluctantly at his father's medical practice. What, he wondered, would be the outcome of all this. Affairs at home were becoming strained. At the end of 1904, after a long period of what amounted to therapeutic banishment to Herne Bay, his mother died. Meanwhile his father had become uncommonly intimate with his dispenser, Emily Maude Pearle, whom he subsequently married (1906). Then, as so often happened in Greenwood's career, when his affairs seemed darkest, a way of escape was offered. At the end of 1905 Leonard Hill had a vacancy on his staff at the London Hospital for a Demonstrator in Physiology. In the face of some opposition, he offered it to Greenwood, who accepted with eagerness. Now at last he was independent of his father, working with someone who was sympathetic to his ambitions, and free to extend his studies under Pearson.

"I was working in divers' sickness at the time," Hill recorded, "and he joined me in the experiments …. We did a lot on dogs and then went into the (pressure) chamber ourselves. I was also editing a second volume of *Recent Advances in Physiology* and asked him to deal with the Special Senses, which he did very well."

Sir Alun Rowlands recalled hearing Greenwood lecture on this subject, and said he lectured very well indeed. From the notes he made, Greenwood published in 1910 his *Physiology of the Special Senses.* It had considerable success, and although long out of print and fifty years old, is still a useful summary of what is known of this subject, and to judge from such library copies as we have seen, is still occasionally consulted.

“By recognising the ability of a student with nothing behind him to show his worth and appointing him my assistant,” said Hill, “I may claim to have started Greenwood on his career”.

Hill had been led to a study of “Divers Cramps” – now known as Caisson Disease or “the bends” – through a general investigation of the physiology of the circulatory system. In 1905 he extended his researches into all the physiological effects of high pressure on the animal organism. The general causes and prevention of the Cramps had been established as early as 1877 by the French physiologist, Paul Bert. They were sure that the human body could withstand up to 7 or 8 atmospheres pressure without serious inconvenience: that under pressure the blood absorbed nitrogen and that rapid decompression caused this to be released in bubbles which caused pain and could cause death. The release of nitrogen could be prevented by slowing the rate of decompression to about 20 minutes for every atmosphere withstood. Although the subject had been more recently investigated there was insufficient detailed data, and very few precise measurements. Hill’s purpose was to remedy this deficiency. He would study the physiological aspects; Greenwood, with his newly acquired biometric skills, would look after the measurements.

Siebe Gorman, who manufactured diving gear and were directly interested in Hill’s results, allowed him to use one of their workshops and provided him with a compression chamber. This was a cylindrical metal tank, large enough for a man to lie in at full length. Access was through a manhole. Inside there were a mattress, electric light, bell and telephone. There was a glass peep hole, usually kept closed by means of a metal shutter. Pipes lead air into the tank under pressure, other pipes allowed for its gradual release.

In the winter of 1905 Hill and Greenwood got to work. Hitherto the highest recorded pressure to which anyone had been exposed was about 88½ lbs per square inch. This was on the occasion of a diver descending to a depth of 204 feet. He had, however, died on his return to the surface.

Hill and Greenwood took it in turns to lie in the chamber and record their sensations as the air pressure was raised. Greenwood, fearing that Hill's greater age and heavier physique would tell against him, insisted on experiencing the higher pressure alone. In November he achieved the record pressure of 92 lbs (about 7 atmospheres) which was reached in 54 minutes. He felt little discomfort, and after decompression over a period of 2 hours and 17 minutes, emerged from the tank unscathed. A pressure of 92 lbs (per square inch) was equal to a depth of about 210 feet. The occasion was commemorated with a handsome pair of sea (*word omitted*) inscribed "In memory of 92 lbs" which for many years occupied a place on the mantle shelf in his dining room.

The experiments led to others. One night his son, sleeping in an adjoining room heard strange noises coming from his father's room. Puzzled and worried he cautiously opened an intervening door and beheld a very strange scene. His mother was asleep. Beside her lay Greenwood with a clip on his nose and a tube running from his mouth to an enormous canvas-like bag on the floor. The noise was Greenwood's breathe passing through a valve on its way into the bag. Greenwood was trying to determine the output of his lungs during a night in bed. Next morning his son helped him deflate the bag through a gas meter.

Meanwhile, with Bacot's assistance, Greenwood tried some experiments of his own, such as the effects of pressure and decompression on larvae and caterpillars. The significance was that these creatures did not possess a circulatory system in which breathed air could be absorbed, and should therefore be free from the bubble effects of rapid decompression. Experiments did in fact show that they were immune. Caterpillars of the goat moth withstood pressures up to 27 atmospheres without ill effects, survived rapid decompression, and pupated in the normal way. Greenwood's work on this was published in the *Journal of Physiology* and the *Transactions of the Entomological Society* in 1906.

As the experiments continued Hill allowed Greenwood to publish accounts of them in the *British Medical Journal,* and use the material as reports to the British Medical Association, which gave him a Research Scholarship for the years 1905/6 and 1906/7. In

1908 he was invited by the Royal College of Surgeons to give an account of these experiments in their Arris and Gale Lectures.

These collaborations brought him very close to Leonard Hill. In March 1907 he left his father's house and took lodgings at number 2, Meadow Road, Loughton, Essex, which brought him within half a mile of Hill's residence, Osborne House, on the London Road. Here he was a frequent caller, most Sunday mornings and often on week day evenings. He had his supper earlier than the Hills and used to arrive just as they were starting theirs. He never ate anything, but sat in a corner chatting learnedly with his "Chief", an interruption which the Hill children heartily disliked. But they grew accustomed to seeing him about the place, with their father in the garden, bathing with him on Sunday mornings in a pond in Epping Forest, and talking endlessly during the long summer evenings. They called him "Wiry". It arose from a comment about his size, when their father added, "Yes, he may be small, but he's wiry, and will probably outlive us all." One of these children, Austin Bradford Hill, ultimately became Greenwood's academic successor.

Sir Philip Mansor-Bahr, roughly a contemporary there, records his impressions of Greenwood and Hill and the London Hospital.

"The impression he conveyed was one of a little slim stooping figure, with a fine-chiselled intelligent face, accentuated by a sharp-pointed nose made more conspicuous by a pair of horn-rimmed spectacles. He would enter the Club dining room closely following his burly and somewhat ponderous Chief, Leonard Hill, walking closely to heel like some faithful dog. The luncheon table became notable for there would foregather William Bulloch, the famous bacteriologist, and Henry Head, the equally famous neurologist. There followed a brilliant exchange of wit, interspersed with serious controversy, as the three combined to bait Head with all manner of questions, interlarded with distorted information regarding his own researches which he swallowed with much merriment.

Although physiology and biometry occupied his serious hours, Greenwood still found time for a great deal of rather random reading, mostly in the direction of history and literature. At the instigation of Bacot and Culpin, he joined the North London Natural History Society, went on cycling expeditions with them, and occasionally read them papers. For a time he flirted with Fabianism encouraged by the views of Pearson and Bacot.

2.4. Opsonic Index Controversy

In 1908 Greenwood, began an investigation which brought him into conflict with one of the most controversial characters in the English medical world, and resulted in his appointment as the first non-government medical statistician in the country.

As his disciple and torch bearer Greenwood had been supplying Pearson with biometric data from the London Hospital Records. Pearson was just then investigating the statistics of tuberculosis, and a hospital full of patients who could describe their families and backgrounds in relation to the ailments from which they were suffering was obviously a splendid source of biometrical data. In November 1907 Greenwood suggested to the Hospital House Committee that a Statistical Department be established.

Although Pearsonian methods were far from being generally appreciated by the medical world – after all Pearson was not even a medical man – they did have some powerful supporters in the Hospital. Leonard Hill was one. Another was William Bulloch (1868 – 1941) the bacteriologist. The House Committee viewed Greenwood’s suggestion with sympathy, and within a few months a Statistical Committee was in fact established. The Hospital authorities went even further. Soon after a Statistical Department was set up with Greenwood as Director and Dr JDC White as his assistant. By October 1908 Greenwood was offering to conduct courses in Biometry. Meanwhile a major event in the development of biological statistics was brewing up. This was the Opsonic Index controversy.

The Opsonic Index was a development by Sir Almroth Wright (1861 – 1947), Bacteriologist at St Mary's Hospital, Paddington, of the ideas and discoveries of Metchnikoff, Koch and others. It had been discovered that the white blood corpuscles, the phagocytes, had the power of absorbing and destroying disease bacteria. This power of destruction varied, and Wright was consumed with a desire to manipulate it by means of vaccines. The quality or condition that enabled the phagocytes to pursue their work of destruction Wright called "Opsonin" from the Greek word meaning "to prepare victuals". Bernard Shaw, who guyed Wright in a friendly way in his *Doctor's Dilemma*, called the process "buttering" the microbes so that the white corpuscles could eat them.

Wright's idea was first to determine the normal capacity of the white corpuscles (phagocytes) in healthy blood to destroy disease bacteria. This was the Opsonic Index. In the treatment of disease he proposed to find out the capacity of the patient's phagocytes to destroy bacteria. If it fell below the Index normal, then he hoped to be able to stimulate it by means of inoculations. Wright's Opsonic Index was the result of much detailed work. In order to find out in quantitative terms the destructive capacity of healthy phagocytes, he had made a large number of tests. For these he took smears of blood on microscope slides, infected them with bacteria of various kinds, and after an incubation period, counted the number of bacteria that had been absorbed. He published his results and the Opsonic Index became internationally famous.

In the period 1907 / 8, however, various criticisms had been levelled at what Wright considered healthy or normal and what he considered unhealthy or abnormal. Wright had set fairly narrow limits for his state of normality. His critics argued that the destructive power of healthy phagocytes might vary much more or much less that the limits suggested by Wright.

This was exactly the kind of thing for biometric examination. The phagocytes were living things. They were subject to chance and change. They existed within precisely the kind of environmental circumstances for which Pearson's methods of measurement were designed. Moreover, there was a special reason for submitting the Opsonic Index to

biometric tests. The Index was "News" in the medical world, and Wright was not only a distinguished controversialist, but he was also a proclaimed opponent of Pearson.

Wright was a rumbustuous and provocative genius with a fine flow of language and a dangerous disregard for caution. Although his writings do contain occasional admissions that biometry might have its uses, he was generally recognised as the leader of the Anti-Biometricians. He regarded the Pearsonian intrusion into medical affairs as impertinent nonsense.

As Wright was a man of distinction in the medical world, he was also a great stumbling block to the progress of biometry in medicine. He was an advocate of what he called the "Experiential Method" in science. This amounted to little more than experienced guesswork, which was the kind of thing biometry was intended to replace. He had, moreover, several times come into sharp conflict with Pearson, notably at high levels in 1904 over the interpretation of the statistical reports on anti-typhoid inoculation of troops fighting in the Boer War.

Who started Greenwood on his Opsonic investigations is not recorded, but it was probably William Bulloch at the London Hospital. Bulloch was himself critical of Wright's Index, and had already done some work on it himself. When Greenwood suggested an Opsonic Investigation to Pearson, the Great Man jumped at the opportunity and gave his full support. Acting in the name of the London Hospital Statistical Department, Greenwood therefore applied to Wright for data and explained his purpose, which was simply to apply biometric tests to the values Wright had shown in his Index. Wright courteously sent him records prepared by his assistant, Alexander Fleming, later to become renowned as the discoverer of penicillin. With the assistance of Dr White, Greenwood got down to work.

It is noticeable that at the start Greenwood's approach to Wright was extremely cautious. He aimed to avoid controversy. He intended simply to publish his results and let the scientific public draw its own conclusions. This was approved by Pearson, who disliked

slanging matches which were neither scientific nor constructive. Once emotional hostilities were aroused he pointed out, all hope of one side convincing the other was ended.

Conviction or conversion in science should only be attempted by means of irrefutable logic – in this case the logic of biometry. Unfortunately in this instance emotional hostilities *were* aroused, and though they took several years to develop, they resulted in Greenwood striking a mighty blow for biometry but seriously offending Pearson.

The more Greenwood and White investigated the Opsonic Index data the stronger grew their suspicion that Wright's method of assessing the destructive power of phagocytes was faulty. The counts varied too widely from sample to sample. Wright's definition of what was normal was too limited. Wright's "normal" might be abnormal in some people, and his "abnormal" might equally be normal. They consulted Pearson on the mathematical intricacies of the problem. He not only gave his advice but set his students working on the problems involved.

When they had submitted their Opsonic Index data to biometrical examination Greenwood and White proposed to publish their findings in Pearson's *Biometrika*. The intention leaked out ahead of time. In January 1908, at a meeting of the Medical Research Club, of which Greenwood had become a member and at which Wright was present, the subject of the Opsonic Index was raised. Greenwood criticised some of Wright's deductions from his experimental work. This lead to an acrimonious dispute between Wright and CJ Martin who was Director of the Lister Institute for Preventive Disease.

What also emerged from this dispute was the disagreeable likelihood that if Greenwood and White's views on the Opsonic Index were unfavourable, the entirety of Wright's supporters would be mobilised to crush them.

The investigation proceeded slowly.

His relationship with Pearson was now pretty close. Having the entrée to the medical Journals, he was glowingly reviewing Pearson's works in these publications. He was also giving lectures on Pearsonian subjects at the London Hospital, notably on heredity. The Master was pleased, but far too busy to attend them. Perhaps this was as well. The Pearsonian doctrines were not always received with proper reverence. In March Greenwood persuaded one of Pearson's personal friends, Edward Nettleship, to lecture on certain aspects of heredity. The disbelievers treated him to some rude criticism. Heredity, like Darwinism and vaccination, was a subject that in those days aroused fierce partisan feelings. Greenwood tried to soothe his guest speaker and hastily sent words of pacification to Pearson. A certain Mudge was the ringleader of the critics. His name repeatedly appears as an unbeliever.

"As a leader, so are his followers," replied the Master tersely, referring to the miscreant Mudge. "If the one mistakes truculency for logic, and rudeness for smart writing, what can you expect of the others?" The dispute was about Mendelism. "The main point, perhaps," he continues, "Will be to save the real good in Mendel from the evil produced by the ignorance of the Mendelians."

The acrimonious dispute at the Medical Research Club between Martin and Wright had important consequences for Greenwood, "Mainly owing to a controversy with Almroth Wright," Greenwood recorded in later years, "I caught the eye of CJ Martin who created a statistical post for me at the Lister Institute."

The Lister Institute for Preventive Medicine was a peculiarly English institution. Intended as an English version of the Pasteur Institute in Paris, by 1908 it had become a private medical research establishment, with the status of a school of the University of London. Its speciality was bacteriology, and by virtue of conditions attaching to a certain bequest of funds, investigations into drinking water. It was controlled by a Council representing various learned bodies such as the Royal Society, and its head was CJ Martin.

Martin's background was favourable towards Greenwood in the situation that had arisen in connection in the dispute with Wright.

Martin (1866 – 1955), like Greenwood, had started his career on a path not of his choosing. He had been born in Dalston Lane, Hackney, not far from Greenwood's own birthplace. Indeed, one of the roads leading off Dalston Lane was named Greenwood Road. At the age of fifteen Martin had left school to become a clerk in an Assurance Company where his father was an actuary. To further his career, he attended evening classes in mathematics. Suddenly he decided to become a doctor. He matriculated, joined St Thomas's Hospital, won scholarships, studied in Germany, became like Greenwood, a demonstrator in Physiology, and did a spell of teaching in Australia where he succeeded Almroth Wright in a teaching post. He returned to England and was appointed Director of the Lister Institute in 1903. At once he became involved in an investigation into Plague in India, and found himself loaded with a great mass of numerical data that required statistical analysis. Moreover, just as he had succeeded Wright in the Australian teaching post, so he had become Wright's successor in the Indian Plague investigation. Martin was of the opinion that Wright had mishandled his opportunities when investigating plague in India. He could hardly ignore the fact that most of Wright's "findings" on that occasion had subsequently been found to be incorrect. Indeed, Martin's most memorable discovery, the relationship between the rat "carrier" and the spread of Plague, Wright had airily dismissed as being unworthy of any consideration at all.

Martin, therefore, had a good deal in common with Greenwood; the same unorthodox beginnings, an interest in mathematics and statistical analysis, and a profound distrust of Almroth Wright. He wondered if he could use Greenwood to analyse the Indian Plague data. It was high time the Lister Institute considered appointing a permanent statistician. Confidential discussions took place: Hill and Bulloch were consulted, Greenwood himself was sounded. But such matters took time, Meanwhile Greenwood suddenly ventured into matrimony.

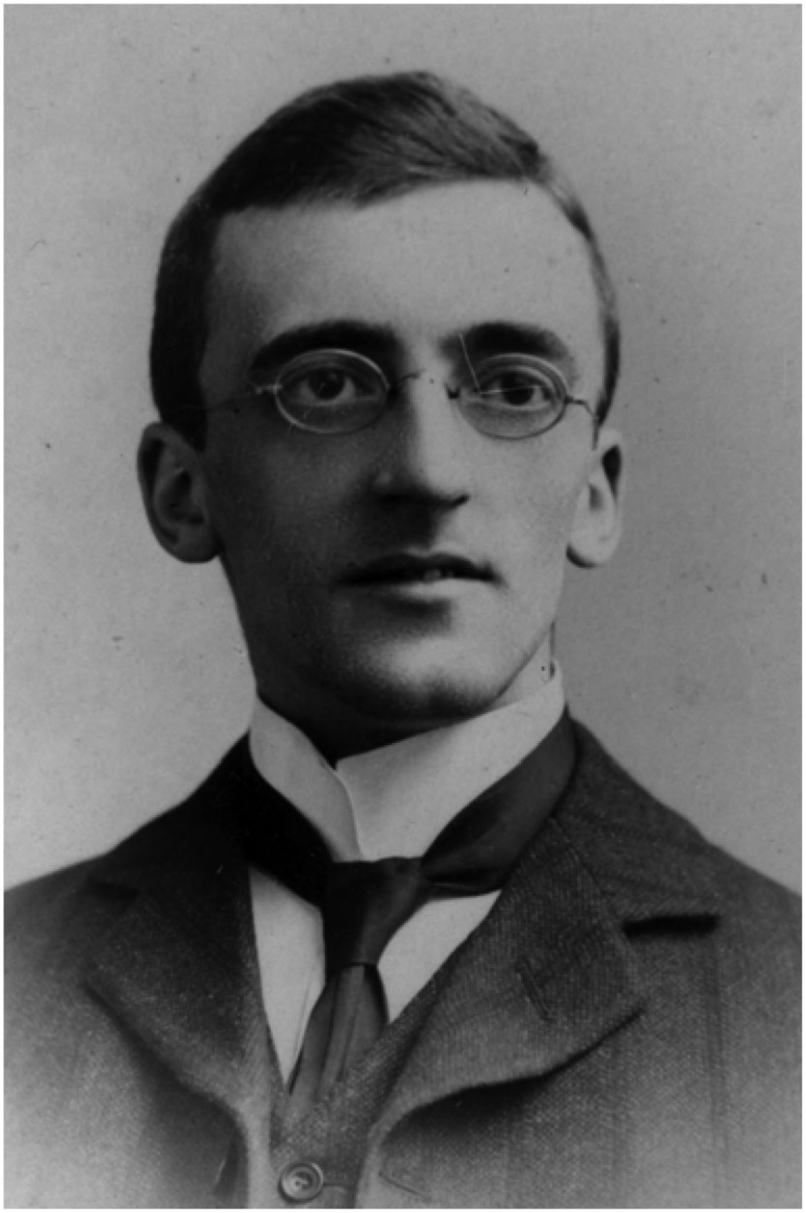

Figure 2: Major Greenwood between the ages of 20 and 30 years (1900 -1910) (provided by Roger Major Greenwood)

2.5. Marriage

Several things about Greenwood's marriage seemed rather odd to outside observers. Here he was, a member of a one hundred per cent Cockney family, extremely reserved towards the opposite sex, brought up in the Church of England with a traditional disregard for Roman Catholicism, yet he suddenly married a charming Roman Catholic girl in the heart of Germany. How did it happen?

“He married to learn German”, was one comment. Another was that he married a German “to change the family strain”. “Germans”, said an aunt in recollection, “were often strong, which the Greenwoods were not.”

Both comments contain an element of truth. His marriage was certainly one of the results of trying to learn the language, and the need for changing the family strain was not wholly overlooked. Matters of heredity and predisposition to disease had been a preoccupation for years. It was one of the main springs of Pearson’s multitudinous researches. Greenwood’s mother and infant brother and sister had died of illnesses thought to have hereditary significance. His great grandparents’ family had been almost wiped out by disease. And there was always that sinister recollection of the so called epileptic fit when he had started at the London Hospital.

As a boy and a young man, Greenwood’s relations with girls outside the family were few and discouraged by his father. The only active encouragement his father seems to have given him in this direction was a hope that he might marry Grace, the attractive niece of his new step-mother. As a boy and a young man he was one of those busy, fidgety people, full of enthusiasms and bursts of energy, ever industrious, delving into this and that, filled with curiosity, never dull.

He was one of the “rowdy gang” at school, and by most accounts given to “ragging”, and not innocent of what he called “brushes with the yokels”. His blind spots were social ones. He was a shy mixer; emotionally reserved; no ability for small talk either dumb or talking too much and talking too smartly. He was anything but a ladies man. His marriage came about in this way.

When he was twenty or twenty one he went to France to improve his knowledge of the language. According to himself, he knew the grammar well enough, but was deaf and dumb for the conversational part. In France he fell in with a young German school teacher named Heinrich Schonder, who was there for the same purpose as Greenwood.

Schonder suggested that Greenwood visited him in Germany. In 1904 Greenwood sounded Pearson on such a project, but as we have seen, Pearson's regard for German biometricians was not high. But Greenwood now had a wider purpose for learning German. He was now a Physiologist and some of the best Physiology was certainly being done by the Germans.

In 1906 Greenwood visited Schonder at his home in Sigmaringen, in Hohenzollern. Ever afterwards he reckoned it among the most beautiful places in the world. It has indeed, great charm. Schonder introduced him to his fiancée, Stephanie, and soon Stephanie introduced him to her friend Rosa Baur. Rosa's father, a widower, was employed in the Finance Department of the Sigmaringen Municipality. Pictures taken of Rosa at this time show her to have been extraordinarily pretty. It can hardly be wondered that Greenwood, amid the delights of holidaymaking in so romantic a spot, and his personal shyness to some extent masked by language difficulties, should have fallen in love.

It seems to have been love at first sight, for Greenwood was already contemplating marriage by the end of the same year. Yet there were certain facilitating circumstances. Both his and Rosa's domestic situations were rather similar. His mother had died a year or two back after a long illness. There had been an embarrassing intimacy between his father and his dispenser. Now his father had married his dispenser, and what had always been a rather strained household, became much worse. He had loved his mother, but he had usually been at loggerheads with his father. Now his mother was gone, and the lady who had taken her place was a former employee, a servant almost, and she ganged up with his father so that both of them seemed to be against him. Marriage was clearly a most satisfactory way of mending his domestic affairs.

On Rosa's side, too, there were complications. Her mother had been dead for several years, and her father's familiarity with the housekeeper was causing trouble, if not open scandal. Rosa too had good reason for wishing to leave home.

Back in the damps of East London, Greenwood decided to press the matter of marriage. He wrote to Schonder for advice. He quoted the snags, among which were language, and the fact that Rosa was a Roman Catholic, and asked if he should dare to propose to her (1906).

Schonder, who had since married Stephanie, considered the matter from every angle. His reply, couched in the quaint English he had learnt, ran to some seventeen hundred words. (January 1907). He began with the sound caution against believing things to be true because one wish them so. This fault was particularly dangerous in people reckoned to be clever. Nobody was more likely to be mistaken about woman than people who were otherwise clever but were unaccustomed to dealing with them. German Professors were notoriously unsound choosers of wives, and many were saddled with real “monsters”.

What Greenwood wanted, he counselled, was a wife primarily devoted to her own family, who would not be distracted by society books, relations, money or clothes. Such distractions were common in wives of the class to which Rosa and Greenwood belonged, but Schonder thought Rosa would be an exception. “She has something so plain, so homely, so sincere about her,” he wrote, “that I am sure she cannot but think and feel naturally in that most natural of all things, the bringing up of her children.”

Language would present no lasting difficulty, neither would the difference in nationality. As to adjusting herself to a new life in England, conditions perhaps favoured it. Rosa had no mother and her father was far from ideal. “She may love him,” said Schonder, obviously in doubt, “I don’t know anything about it. But she will love her husband much more for the fact that he takes her away from her home, which is not the ideal of a home at any rate.”

On the matter of religion, some kind of accommodation could be arranged, even though Rosa did wonder if Greenwood’s scientific attitude might make him sceptical of religion. Indeed, to Stephanie she had already expressed concern about his soul, which was surely a promising sign, and to Schonder she had wondered if he really believed in religion at

all, which was not so satisfactory. Schonder was in much the same position as Greenwood. He was a protestant, and his wife a Catholic. They had settled by agreeing to bring up the children in what Schonder called "the poetic faith".

As for money, Greenwood's supposition that he should wait until he had £250 a year was needless. £150 a year was probably enough on which to start married life - £50 for rent, the rest for living. As to furniture, Schonder said that in Germany the bride was expected to pay for practically everything not actually worn by the bridegroom. "Even his gold watch she mostly presents to him when they engage. She is supposed to possess clothes and such things for a year or two, and linen and so forth for thirty or forty years."

Having consulted the most learned of his colleagues on the problem, Schonder came to the conclusion that if Greenwood and Rosa were in love, then he should propose to her. Thus reassured, Greenwood did propose and was accepted. A most elaborate announcement of the engagement was then sent to all his relatives.

With Mrs. Hill's help, he rented a house in Lower Park Road, Loughton, and collected together some second hand furniture. Then, in July 1908 he returned to Germany. He made a pact with the Roman Catholic Church not to hamper his wife's religion, and to have his children brought up in that faith. On the 28th they were married at Kloster Beuron, a few miles up the Danube from Sigmaringen. He carefully observed his pact with the Catholic Church, but he never really forgave them for having forced him to make it.

There were no invitations to the English relatives to the wedding. They would never have come anyway. The whole adventure seemed to them slightly crazy. Greenwood went alone, travelling in the clothes he wore at the ceremony, including, so the story goes, a tall hat, which gave him endless trouble on the train.

He and Rosa returned to England early in August. On arrival in London they went round the town on top of an open bus. Then they went down to Loughton where Mrs. Hill had been airing the rooms ….

"Most hearty congratulations," wrote Pearson when he heard about the wedding. "It is quite the best state of affairs. I only wish I had begun it earlier." Then he plunged into a matter of mathematics … and so in a sense did Greenwood. Married or single, he had his biometry and physiology to occupy him. To poor Rosa, alone in a foreign country, with only a faulty knowledge of the language, the prospect was frightening. She was homesick, "far beyond tears", she confided later. Indeed her early doubts about her fate in England had caused her to hide among her underwear sufficient German money to get her back to Sigmaringen. She was homesick until her children were born, then she was happy.

3. The Lister Institute (1910 – 1914)

3.1. Appointment to the Lister Institute

By the spring of 1909 CJ Martin had persuaded the Governing Body of the Lister Institute of the importance of biometric methods to medicine. The Governing Body agreed in principle to the appointment of a biometrician – or a statistician – to their staff, and justified its decision by pronouncing "that the validity of conclusions drawn from many enquiries in experimental medicine, as well as those regarding the practical value of prophylactic and curative treatment, must ultimately rest upon the statistical analysis of the results, and that statistical treatment would aid in defining the relative importance of different means whereby disease is spread."

This was not only important to Greenwood; it was important to statistics as a whole. It amounted to official recognition of statistics as a necessary tool in medical research. The Lister Institute did not represent all the medical profession but it did represent a progressive section of it.

It is noticeable at this point that the term “statistics” more and more takes the place of “biometry”. This marks a new development. Pearson had developed biometry in the interests of evolutionary research. The pure Pearsonians continued to study evolution, and to toss out as bye products new and ingenious mathematical techniques. But there were other disciples of Pearson, like Greenwood, who saw that these techniques could be used for much wider investigations. They were the developers of mathematical statistics in the wider sense. Greenwood’s fame ultimately rested on his pioneering application of biometric techniques to medicine. The moment he broke free from Pearson’s tutelage, his interest in evolutionary theory vanished. “Biometry”, as a piece of nomenclature, became increasingly reserved for statistical studies into matters of heredity, and “statistics” to relate to the mathematical investigations of any kind of data.

In April 1909 Greenwood hinted to Pearson that he might be offered a statistical appointment. Pearson replied, “Of course I should say GO in for statistics, if the post is a good one wholly,” but seems to have been ignorant of the details.

By now Greenwood and White’s first investigation of the Opsonic Index had been published in *Biometrika*. It was purely objective, and drew attention to the difficulties associated with the method of counting phagocytes, bacteria and so forth. In February he had discussed the matter at a meeting of the Pathology Section of the Royal Society of Medicine. His remarks were published in the *British Medical Journal*, together with a favourable editorial comment.

By now he was a frequent book reviewer for the *British Medical Journal*, and did good service to Pearson and his supporters. Referring to a review he was preparing of one of Pearson’s recent publications, he wrote to the author (May 22, 1909), that after a first glance at the work in question, he was filled with “an enthusiasm which demonstrates that I am really not a proper person to review your publications as enthusiasm is no suitable frame of mind for a judge. Accept my heartiest congratulations. You are my Magnus Apollo.”

His industry was endless. He was busy collecting case histories of tuberculosis from hospital patients, and already had records of nearly five hundred.

In June 1909 CJ Martin tentatively suggested a statistical job at the Lister Institute. Greenwood agreed subject to certain guarantees and conditions.

What his "guarantees" and "conditions" were we are not told, but freedom of action within his own sphere was almost certainly one of them. It is also probable that he suggested that G Udny Yule (1871 – 1951) be associated with the job. Yule had been Pearson's assistant at University College and had taken avidly to mathematical statistics. He was a trained mathematician, and had made several fundamental contributions to mathematical statistics. Like Greenwood he had started as a disciple of the pure Pearsonian canon, but had gradually diverged from the Master's path. He had no real interest in evolutionary theory, and like Greenwood proposed to use Pearson's techniques for other purposes. In 1899 he had left Pearson and joined the examining body of the City and Guilds Institute. In 1902 he secured the Newmarch Lectureship in Statistics at University College. Greenwood first met him shortly before 1907 and got on well with him. He was a kind of junior KP, a trained mathematician on whom he could rely for technical help. He was a gentle person, with whom one could discuss a point without fear of emotional explosions. With Pearson, Greenwood must always remain a pupil; with Yule he could become a colleague.

As we shall see, Greenwood and Yule together formed a formidable combination, and as Greenwood drew nearer to Yule, he drew further away from Pearson.

Towards the end of 1909 the Governing Body of the Lister Institute agreed to Greenwood's "conditions", and he was officially appointed as the Institute's Statistician, with Yule taking the post of honorary adviser. At the same time he was elected a Fellow of the Royal Statistical Society, Yule and Sir Arthur Newsholme, the Chief Medical Officer for Local Government, standing as his sponsors.

So at the age of twenty nine Greenwood became the first non-government medical statistician in the country.

3.2. Indian Plague

The Lister Institute, completed in 1898, was and still is, a handsome building at the river end of the Chelsea Bridge Road. Here in the autumn of 1909 Greenwood set about establishing a Statistical Department. His first major assignment was an investigation into the incidence of Plague in India, based on statistical data prepared by the Indian Plague Commission.

His aim was to answer three questions:

1. How does the disease enter a given country or district?
2. Having effected an entrance how does it maintain itself there?
3. What circumstances determine the transformation from endemic to epidemic prevalence and conversely?

He was to spend a good deal of his life attempting to find the answers to these kind of questions, and on the plague enquiry he laid the foundations of his reputation as an epidemiologist.

The aspect of epidemics that continued to fascinate and perplex him, was their periodicity, the strange cycles whereby a disease would be present (endemic) but not active for long periods, and then, for no apparent reason, spring into furious and contagious activity.

Why, for instance, did plague disappear from England at the end of the 17th century and why was it inactive for long periods in India? (As a matter of interest there was a minor outbreak of plague in East Anglia in 1906. Small groups of deaths had occurred that had at first been diagnosed as due to a virulent form of pneumonia. Plague was not recognised

until 1910. Plague infected rats persisted in the area until at least 1918. Why, since the rats were disseminating the disease had no serious epidemic occurred?).

Since it was now established that rats carried the fleas that carried the plague bacteria, Martin began looking for someone to study the life cycle of fleas. As there was no qualified entomologist ready to hand Greenwood suggested that Arthur Bacot might be useful in this field. He introduced him to Martin and they immediately took to each other. They had much in common. Bacot's study of entomology had begun in the kitchen of his lodgings at Bow; Martin's scientific studies had begun in a shed at the end of his Parents' garden at Hackney. Martin had begun life in an Assurance Office and hated it. Bacot was still in an Accounting Office and hated it no less. Martin longed to offer him a means of escape, but there were difficulties. Although Bacot had studied entomology for twenty years and was a first class microscopist, he was still an amateur with no professional standing. The Lister Institute was largely supported by public funds and it was inconceivable that the Plague Committee would agree to the appointment of an amateur. The best that Martin could arrange was that Bacot should take up the study of fleas in his spare time, and that the Committee would pay his expenses and give him an honorarium. It was a kindly gesture that paid off handsomely as we shall see.

3.3. Opsonic Index Enquiry Continues

Meanwhile Greenwood and White were pressing on with their enquiry into the Opsonic Index. Their first investigation had been based on limited data. Now they obtained data relating to the number of bacteria absorbed by some 20,000 white corpuscles. White, who worked in the Inoculation Department at the London Hospital, prepared the bacteriological material and did the counting. Greenwood's job was to look after the mathematical side, to find out how the absorption power of the phagocytes varied from sample to sample, and relate this variation to the whole range of material used. The actual counting was complete by December 1909, but Greenwood was at loggerheads with Pearson over the mathematical handling, and progress was slow.

Greenwood's appointment to the Lister Institute had strengthened his position in relation to the Master and where their opinions differed, he was now inclined to be rebellious. However, after a great deal of discussion the Paper was ready for publication, and finally appeared in *Biometrika* in November 1910. This might have been the end of Greenwood's interest in Opsonic matters. The paper was strictly objective. It contained nothing deliberately controversial and there was no reference to Almroth Wright. Pearson had seen to this. The Paper was greeted with silence – or so it seemed. But Wright had read it and was determined not to let it pass unscathed.

3.4. Predisposition and Alcoholism

Greenwood's relations with Pearson had long since extended to the social side. In May 1910 he was suggesting a cycling expedition round the Essex churches, but Pearson was far too busy to join him. In June he invited Greenwood and his wife to an "at home", and in July, in a mood of biometrical enthusiasm he was wishing he had a grant of £4,000 a year to establish a great Statistical Laboratory, and could ask Greenwood and his other biometric disciples to join him there.

Pearson was just then involved in two major controversies, whether predisposition or infection was the dominant factor in tuberculosis, and whether chronic alcoholism in parents affected their offspring in a hereditary sense. Both, but especially the later, were of public concern and at times brought the disciples of biometrical studies into the national press. Pearson was the focus of controversy but all the time Greenwood acted as his lieutenant.

Pearson's interest in both matters lay in their hereditary implications. He was, basically, searching for Darwin's evolutionary processes, how one species evolved into another. Could children really *inherit* tuberculosis from their parents as they inherited other characteristics? Or did they merely catch it by way of infection. He was inclined to believe that the disease could be transmitted through heredity and published some evidence to support this view but recognising that the disease was also undoubtedly infectious was angered by a government statement that tubercular stock could safely

marry provided they got a good supply of fresh air. This he refuted and earned the enmity of Arthur Newsholme, the Principal Medical Officer of the Local Government Board, who thereafter became an opponent of the biometrical school of thought.

The alcoholism controversy was much more violent and became a national affair.

To most people of intelligence it seemed only reasonable that excessive alcoholism in parents should have some deleterious effect on the mentality and physique of children born to them after they had succumbed to this state. To Pearson this belief had a special significance. If it were true, it would seem that alcohol could in some way interfere with the natural laws of heredity. He and Ethel M Elderton conducted a biometrical investigation into the problem based on data on alcoholics in Manchester and Edinburgh. They published their findings in 1910, which showed no significant relationship between the intelligence, physique or health of children and their alcoholic parents. In short, they found no evidence that alcohol affected inherited qualities.

This enraged the advocates of temperance who raised a cry of protest. Foremost among them was Sir Victor Horsley, an eminent surgeon, who had been heading a campaign against alcohol since the beginning of the century. Horsley, it will be recalled, was one of the "experts" called in to diagnose Greenwood's quasi-epileptic fits in 1899. He was a lusty, bigoted opponent, who referred to all who touched alcohol, no matter how abstemiously, as alcoholics. In one of his wittier moments he gave John Bull and Father Christmas as typical examples of fatty degeneration due to excessive drink. He led the "popular" side against Pearson, who seems to have been astonished at the furore he had caused.

Pearson as a controversialist was superb but among the medical profession he suffered the damning handicap of not being a medical man. All through their early years the biometricians had suffered from this handicap, and it fell to Greenwood, who *was* a member, to at least provide a key that would unlock his profession's disbelief.

3.5. Scientific Papers

Greenwood was now well launched into the way of reading papers before the learned societies. He started as a passionate preacher of the biometric doctrine. Many of his early papers were associated with the current Pearsonian controversies, but he gradually introduced wider aspects of biometric application. On February 21st 1911 he read his first of many papers before the Royal Statistical Society. This was a Study of Hospital Mortality Rates from 1751 to 1901. He had extracted his data from the London Hospital records with Dr RH Candy. It was one of the efforts of the London Hospital Statistics Department, begun before he moved to the Lister Institute. "Before long," Candy recalled many years later, "Greenwood went to the Lister Institute and it was there that I used to go for all the latter part of the work on pneumonia in which I did the donkey work, whilst Greenwood supplied the brain power. He was extremely kind to me in all sorts of ways. He was a remarkable man …" Candy joined the Army Medical Service, went to India and subsequently retired as a Major General.

Greenwood joined the Council of the Royal Statistical Society the following year and remained on it almost continually until his death. He became Honorary Secretary in 1919 and only retired from this position when he was elected President in 1934.

In April 1911 he took up cudgels for Pearson before the Epidemiological Section of the Royal Society of Medicine, arguing, with the support of Bulloch, that predisposition was an important factor in the transmission of tuberculosis. Nobody, it seems, could be found to controvert this view.

3.6. Charles Creighton

Greenwood's attendance at the Epidemiological Section brought him in touch with the learned Charles Creighton. He was introduced to Creighton by William Butler, a Glasgow trained physician then in the Public Health Service in London. Creighton, who was born in Scotland in 1847, had produced between 1891 – 4 a monumental *History of Epidemics in Great Britain*. Greenwood, already predisposed towards historical subjects, immediately fell under the spell of Creighton's curious learning. Although Creighton had

contributed enormously to the study of epidemics, he appeared to his contemporaries as something of a crank. He disbelieved in the efficacy of vaccination, and was known as the anti-vaccinator. He had even written a book disparaging Jenner, and referred to the new science of bacteriology as the "microbic theory". His learning was immense and his conversation fascinating. Greenwood said of him: "There cannot have been many better literary anecdotists than Creighton: he seemed to have read everything and expounded theories of authorship and explanations of mysteries in theological literature with as much zest and confidence as epidemiological doctrines. He knew who wrote *Revelations*, who the Beast was, and was quite sure that the four beasts were the prophets Isaiah, Jeremiah, Daniel and Ezekiel. It would have taken a very well read man to stand a chance with him in a literary tussle, but he did not bully young people. He had a sense of fun, but his table talk suggested a certain aloofness from, even contempt, for current medical thought."

It is difficult to tell how far Creighton influenced him. Perhaps their interests merely happened to coincide, and Creighton's possession of them confirmed Greenwood of their propriety. Certain it is that Greenwood's opinion of Creighton quoted above might equally be applied to himself. He had the same odd literary taste, the same passion for mixing his subjects epidemiological, statistical, theological, historical, the same aloofness from contemporary medical thought.

3.7. Friction with Pearson

It will be recalled that when Greenwood secured his position with the Lister Institute, GU Yule had agreed to act as a kind of honorary adviser to the Institute on matters of statistical technique. Consequently as the months went by, Greenwood, who was keenly conscious of his lack of mathematical training, leaned more towards Yule for advice and less towards Pearson. Pearson was aware of this, and it pained him for there was a rift between him and Yule.

When Yule's *Introduction to the Theory of Statistics* was published in the summer of 1911, Greenwood wrote to Pearson, "I don't swear by the whole of Yule's book but I

think with submission, that it will do much more good than harm, if the average Philistine can only be got to read it."

But this was just what Pearson could not admit, for among other deviations from the Pearsonian canon, Yule had actually sought to better Pearson's use of the chi-squared test. This was one of Pearson's most cherished innovations. It purported to show whether a factor observed in data was normal or the effect of chance or some other cause. It was particularly galling to have it called into question by a former pupil and assistant. He replied, "I am afraid I can't agree about Yule's book. It will do a great deal of harm, because people will use methods which are quite fallacious, because they are easy, and this is bound to lead to a catastrophe."

The rift between Yule and Pearson is not without interest because it finally engulfed Greenwood as well. Pearson's genius was bold and hasty; Yule, perhaps an inferior mathematician with less imagination, was cautious and critical. When they had collaborated at University College they had been the best of friends and spent several holidays together. When Yule developed his own line of statistics and ventured to criticise some of Pearson's methods, the latter was understandably annoyed. He could never endure to be criticised in the field of science he had done so much to develop, and "never", Yule asserted, "admitted a bad blunder". Yule's criticism of KP's use of the chi-squared test called forth years of controversy, and culminated in a 152 page long defence by Pearson and his assistant Heron (*Biometrics* 9, 1913). This MG Kendall commented was remarkable for having missed the point over more pages than perhaps any other memoir in statistical history.

There were personal reasons as well as differences of scientific opinion to drive the men apart. Soon after Yule had left Pearson to join the City and Guilds Institute, he secured the Newmarch Lectureship at University College (1902 – 9), and conducted a series of courses on statistics which Pearson construed as being in opposition to him. Yule's lectures were free, and constituted such serious competition that Pearson lost students,

and to get them back had to provide much fuller instruction and more practical work than he had intended.

The final break came in 1905 when Yule rather carelessly asked KP to present a paper he had written some years earlier to the Royal Society, forgetting that it included a criticism of Pearson's methods. The incident ended their friendship for ever.

By now Greenwood and Yule had become fast friends, and their thirty eight year long correspondence had begun. They no longer confined themselves to statistical matters, and both naturally shy, had begun to share each others confidences. With Pearson, Greenwood attempted to steer a middle course, and a vein of humbug creeps into his letters. While exchanging ribaldries with Yule about the "Carlovingians", he continued to address Pearson with elaborate reverence. Eventually, when he was obliged to declare himself on Yule's side, collaboration with Pearson became impossible.

3.8. Advancement

Meanwhile in the summer of 1911, largely it would seem at the instance of Martin, Greenwood was appointed second in command of the administration of the Lister Institute. By now, thanks to the demands of the Indian Plague Investigation, he had established a fully equipped statistical department, and told Pearson that he had "probably more statistical data than ever existed before". It was now time to apply statistical investigation to diseases nearer home, and he proposed to do so on cancer. With this in mind he sounded Pearson on securing the services of David Heron, one of Pearson's assistants at University College, who had already begun an investigation on this disease. It brought a sharp rebuff and their correspondence ceased for six months.

3.9. Arthur Bacot at the Lister Institute

In December 1911 a sudden happiness, as he put it, entered Greenwood's life at the Lister Institute. The Trustees decided to establish an Entomological Research Unit and largely due to his influence Arthur Bacot was appointed to take charge. Bacot's report on the

bionomics of rat fleas had at last admitted him into the charmed circle of research workers.

Perhaps his most significant contribution to the Plague Investigation was his demonstration of the mechanics by which fleas transmitted plague bacilli into their human hosts. At the risk of over-simplifying a complex matter the puzzle had been this; when plague infected fleas bit human beings they sucked blood *out*. How then did they manage to pass their own plague infected blood *into* their hosts? Bacot demonstrated with a great deal of microscopic proof that in fleas infected with plague bacilli the foreparts of their stomachs often became blocked by the growth of these bacilli. Fleas so affected could not properly draw fresh blood into their stomachs, and in attempting to do so, regurgitated and thus drove infected blood from their own bodies into the bodies of their host.

Bacot got on famously with the staff of the Institute and his easy conversational ways soon made him everyone's friend. By contrast Greenwood lacked his social graces. According to a member of the Institute who remembered him from the early days, he hardly seemed to fit into the cosy atmosphere of the place. He was a great talker at the tea-time get-togethers but he lacked Bacot's ability to avoid wounding other people's self-esteem. If he saw a weakness in anybody's defences he was inclined to take a jab at it, He seemed to have a chip on his shoulder and gave the impression that he rather despised the scientific workers who had prosperous family backgrounds as though only those who were poor and struggling were genuine researchers.

"Bacot", said a contemporary, "was marvellous fun, bald headed but bearded, rag-tag clothes and very odd meals … But Greenwood, well he had a phenomenal memory but he had an inferiority complex and that is fatal in a clever man … Perhaps he was bullied at school. He was certainly sensitive about his height. But he grew mellower as he grew older and I expect changed when he realised that he had "arrived".

To anticipate a little, the Bacot-Greenwood relationship grew closer. A year or two later Bacot and his sister moved to a house in Loughton where he set up his entomological laboratory in a wooden shed under some hazel trees in the garden. Greenwood took a larger house nearby, and they spent much of their spare time together. They began a ritual that was to last for many years. Every Sunday morning before breakfast, when the weather was fine, they cycled into Epping Forest and bathed in the Wake Valley pond. After breakfast in all weathers, when the bells were ringing for church, they met again and walked with their dogs in the forest until lunch-time.

3.10. Battling for the Faith

As Pearson's biometrical apostle Greenwood was untiring. He lectured, he spoke up at meetings, he reviewed books and wrote to the press whenever opportunities presented themselves for propagating the Faith. But untiring apostles sometimes become an embarrassment. They have a tendency to change the message and supplant their Master in the public eye. Unconsciously at first Greenwood was doing just this.

Pearson had developed the Faith for purposes of evolutionary theory. Greenwood adapted it for application to matters of public health. He was no Evolutionist, but nor was Pearson a medical man. Pearson's narrower cause was of interest only to the few. Greenwood's applications could possibly affect the health of many. As he pushed his version of the Faith his reputation expanded, whereas that of the Master's became as it were petrified, touched perhaps with a hint of crankiness.

In 1912 they appeared to be battling together, shoulder to shoulder. Heretics, unbelievers and rank pagans abounded. It was extraordinary how medical men, even scientists, disregarded the most elementary mathematical aids to their investigations. A medical degree, it seemed, qualified a man to make any pronouncements he liked on mathematical matters provided the subject was medical.

The chief opponents were Sir Victor Horsley and Sir Almroth Wright who complained effectively of the interference by mathematicians in the affairs of medical men. Their

reputation in their own fields of study was so high that editors were over-awed by them and were inclined to censor Greenwood's comments on their views on biometry. Even Pearson became uneasy about his disciple's boldness in defence of the Faith.

Then suddenly Sir Almroth Wright struck back. He launched an attack on biometricians in general and Greenwood in particular. In responding to it Greenwood lost the tutelage of Pearson and discovered his own strength.

3.11. Quarrel with Wright

In the autumn of 1912 Sir Almroth Wright published a bacteriological treatise bearing the odd title, *The Technique of the Teat*. After explaining his ideas on the Opsonic Index, Wright devoted a special section to attacking the biometricians. It was provokingly entitled, *A Consideration of the Contention of the Mathematical Statistician that he has Authority to Pronounce upon the Number of Leucocytes which require to be counted in the Opsonic Film*. This was a direct reply to Greenwood and White's Memoir on the subject in *Biometrika* (1910). Wright referred scathingly to armchair statisticians and the deplorable effects that had been produced by handing over the adjudication of medical results to lay mathematicians. The only name mentioned, and that but once, was "Mr. Major Greenwood". It would have weakened Wright's argument to have admitted that Greenwood was a medical man himself.

On October 15th Pearson suggested to Greenwood a reply in *Biometrika*. "Are you prepared to write it – quite a short note – or would you like a joint one?"

On October 17th Greenwood said it would be an honour to co-operate in a reply to Wright. He preferred not to publish anything under his single name because a) he had already replied to Wright's general criticism at the Medical Research Club, and to do so again might be attributed to wounded vanity, and b) as medical opinion was largely a matter of Authority, there was little point in pitting himself against Wright's great reputation.

"I am sure," he continued, "it is both for my own happiness and my usefulness to publish only research work, however crude, and if people say it is rubbish let it go at that."

He was, in fact, extremely hurt. "As to whether Wright's statements are due to muddle-headedness' or dishonesty," he continued "I think they are due to neither, but the product of pure contempt. Wright thinks I am a fool and treats a fool according to his folly – you will note that he carefully abstains from any reference to your paper; his assumption is perhaps not entirely devoid of justification although I naturally do not accept it …"

For the moment Greenwood nursed his wrath. Worse was to follow. A few weeks later Wright published what Pearson and Greenwood called the *Rand Report*. In 1911 Wright had been invited to South Africa to advise the Witwatersrand Native Labour Association on the possibility of reducing native labour losses in the mines through pneumonia. Even Wright's admiring biographer and colleague, Dr Leonard Colebrook admits that it had been a hasty visit, productive of nothing new. Wright did, however, encourage large scale and somewhat indiscriminate inoculation, which may or may not have been beneficial. His Report was confused and inconclusive. Possibly sensing that it was wide open to adverse criticism, he included a long, complicated, and largely irrelevant attack on mathematical statisticians. This, according to Colebrook, was Wright's last word on the place of statisticians in medical affairs. Large parts of the Report were published in the medical press.

This time Greenwood was stung to the quick. So long as Wright confined himself to sneering at the Opsonic criticisms, he could keep silent. "But," he wrote to Pearson on November 12th, "if his wider statements be just, any person whose income is derived from holding a post as medical statistician is really either a fool or a knave, because he is taking money for nothing. So far as I know, I am the only person in England not in Government employ who is paid to deal with definitely medical statistics. Therefore Wright's cap can fit no-one but me. In acknowledging the receipt of Wright's paper, I have told him that I will reply to his charges."

"With your permission (subject of course to your approval of what I have written when you see it) I should like the lists to be set up in *Biometrika*."

Pearson was at once cautious. His general practice, as Greenwood well knew, was to employ *Biometrika* only for the publication of new information, and he was reluctant to use it as a medium for controversy.

Greenwood knew equally well, he did from time to time make devastating exceptions. He considered that the present occasion justified an exception, and sent Pearson a rough draft of his reply. Pearson read the draft and did not like it. He suggested the *Lancet* as a better medium, where whatever Greenwood chose to write would reach a wider medical public.

A note of tension entered their correspondence. While agreeing that Wright was "an enemy not merely of biometry but of all science whatsoever" (Greenwood) and that he was "fighting for something more substantial than Truth" (Pearson), they could not agree on how the heretic should be handled. Pearson was for a dignified scientific refutation, Greenwood, who was suffering from a bout of inferiority complexes, and could not forget the great esteem with which Wright was generally regarded, preferred flattery followed by rapier thrusts. Pearson objected to this. On December 17th he wrote him a stern letter making his views abundantly clear, and signing himself "Yours sincerely" instead of the customary "Yours very sincerely". Pearson's epistolary style was full of danger signals of this kind. When on the best terms he wrote "My dear Greenwood". Any cooling off, and the "my" was dropped.

Having expressed his views, Pearson again recommended the *Lancet*, or if they would not take the article, *The Quarterly Journal of Medicine*. If Greenwood insisted on *Biometrika* – and Pearson's tone was forbidding, he would consider the matter again.

On December 19th Greenwood sent Pearson what was intended to be a soothing letter. "Wright is the great idol of the market place just now and a clear demonstration of his clay feet would be a great help to the cause of which you are the leader and I but a

humble foot soldier *very much* in need of your tutelage *if* you please" (Pearson, in his letter of the 17th had remarked coldly that Greenwood was now far from his tutelage being of any profit). "The joke about Wright is that the last time he went out to investigate the causes of disease in the grand manner was the Indian Plague Commission of 1901, the report of which is practically his own work. Martin was telling me the other day that *practically every one* of the findings of that Commission is now known to be wrong. Among other things they dismissed the probability of flea transmission as "hardly worthy of serious mention"."

But it was no use. Pearson remained stiff and aloof. He did not like Greenwood's proposed reply to Wright, and preferred neither to publish it nor be associated with its contents.

On January 18th 1913 Greenwood's reply to Wright was published in the *Lancet*. It was entitled, *On Methods of Research Available in the Study of Medical Problems, with special reference to Sir Almroth Wright's recent utterances*. It was a lengthy document, which ran to some 23 small octavo pages in the offprint, and deserves to be read by anyone interested in the early days of medical statistics. It begins humbly, admitting Wright's great work in his special sphere of study, regrets his mistaken views on biometrical methods, and employs the words of Cromwell, "I beseech you, in the bowels of Christ, think it possible you may be mistaken"

He then outlined his and White's investigation into the Opsonic Index, examined Wright's analysis of Scientific Methods, pulled Wright's "Experiential Method" about, and then outlined "The Method by which a Therapeutic Problem ought to be investigated." On the surface the production seems quite courteous, but underneath are some very sharp digs that must have annoyed Wright intensely. Wright's "Experiential method", for instance, lay wide open for ridicule, in that it could only be used by prophets since nobody had any experience at the beginning of a piece of original research. He concluded with the view that statisticians could afford to ignore the opinions of people like Wright, who, never having taken the trouble to familiarise themselves with the aim

of the statistician, simply did not know what they were taking about. As if to balance Wright's fondness for using medical dog-Latin, one or two Latin tags were thrown in for good measure.

All in all it was an admirable statement of the new techniques and was quite unanswerable on any scientific basis. It received a good – if guarded – press and no-one seriously attempted to rebut it. It more or less marks the end of open medical hostility to the new statistical ideas. It also marked the end of Greenwood's apprenticeship to Pearson. Following its publication, Pearson sent Greenwood a stiff letter which has not survived. Its existence is referred to in a letter to Greenwood from Yule dated January 30th 1913. "I am very sorry for Pearson's letter; it really is too d….d stupid – especially to write as if appointing a consulting statistician cut you off from any necessity or desire for ever writing to anyone else. Damn the man; I've burnt the letter."

It can almost certainly be inferred from this that Greenwood, having endeavoured to continue his correspondence with Pearson, had been brushed aside with the remark that as Yule had been appointed consultant statistician to the Lister Institute, Greenwood had better address his mathematical queries to him in future.

3.12. George Udny Yule

By now Greenwood's friendship with Yule had progressed from mathematics to warmer social confidences. If Arthur Bacot was a kind of rustic, home-spun substitute father in Greenwood's imagination, Yule was an academic brother. They began a correspondence that lasted until Greenwood's death. Much of the Yule-Greenwood and Pearson-Greenwood correspondence survives. It is in marked contrast. For the biographer, except for a few letters, the Pearson correspondence is largely sterile. It is business stuff, the business being mathematics and the propagating of the Faith, the writers Master and Pupil, rarely emerging as human beings at all. The Yule-Greenwood correspondence is much more human. The writers wrote as equals, with Greenwood increasingly becoming more equal. Even so the reader is tempted to wonder, as he reads through hundreds of

pages, how it was possible for such old friends to write to each other weekly and in total say so little about their real lives.

Perhaps because they each had peculiar Christian names – the George in Yule's name seems to have become lost and he was usually referred to as Udny Yule – they addressed each other as "My dear Man" (Yule to Greenwood) and "My dear Chap" (Greenwood to Yule). But Greenwood frequently varied the form according to the current joke. There was "Dear Uncle Yule" when it became necessary to make him an adoptive "uncle" in relation to Rosa's two children. There was "Dear Rechnungsrat" when Yule was curious about the exact significance of this official title born by Greenwood's German father-in-law. There was "Lieber Herr Kollege" when Yule was about to set out for an Austrian holiday.

Pearson came in for a lot of mockery and they both delighted in making fun of his more ex-cathedra pronouncements. One suspects that their search for "truth" in respect of Pearson's work was less for the glory of science than the pleasure of proving the Master to be wrong.

When Pearson, who had become Galton Professor of Eugenics at University College, began organising his new Laboratory, Greenwood wrote to Yule on July 19th 1913, "I am going to design a brand new uniform for all the members of the Galton Laboratory Staff. The full dress is as follows: *Hat*: shaped like a normal curve, with the figure .5 embroidered all over it. *Coat*: Frock coat made of Albinotic hair with buttons engraved YULE erased by a bar sinister. *Trousers*: of brown paper supplied by the publishers of *Biometrika*, with a fourfold correlation pattern. *Baton*: one rectangular brick inscribed, "It is idle to deny that I am right."

On April 22nd 1913 Greenwood commented on the current scandal; "What do you think of Lord Alfred Douglas? I think this blooming country is socialistic in the wrong place. Whether a man is or is not a b….r concerns at most two people. Why not leave these

points to private enterprise. If one is nervous about one's sons one can provide them with leather seated breeches and braces padlocked to the trouser buttons."

Meanwhile, with two growing sons, he had come to the conclusion that his house in Lower Park Road, Loughton, was too small, He had thereupon rented a much larger detached house, *Hillcrest*, on top of Church Hill in the same village.

Inviting Yule to his new house for the Whitsun week-end, he comments; "We have got a room for you and I will leave a Bible and current number of *Biometrika* on the wash stand so that you can feel more Christ-like than ever." This was a reference to a remark by Yule, "I'm so proud of being credited by Karl Pearson with disciples – sounds so Christ-like".

In those peaceful days before the First World War, Greenwood and his friends did a lot of cycling in the eastern counties. Bacot's nature studying jaunts had started the thing off, but Greenwood made ancient village churches his special object. How far he appreciated them from an artistic point of view was never quite clear, but he developed a rather mechanical knack of identifying the particular architectural styles, and spent most of his time strolling round these buildings picking out fragments of "First pointed", "Perp" and "Decorated."

Among his friends he had the reputation for being a hard rider, and several of them complained that he practically killed them with his long sustained exertions.

His letters to Yule are full of arrangements for week end cycling tours, most of them in Essex.

At Whitsun 1913 they went to Thaxted, Bishops Stortford, and Danbury. In the summer Yule went to Austria, taking with him a complicated piece of algebraical work which Greenwood warned him might be mistaken for a cipher message relative to the Austrian

fortifications. Greenwood's wife and children went down to Sandown in the Isle of Wight, but he stayed at home a "grass bachelor" working on his statistical affairs.

It was during this peaceful period (July 25th) that in a letter to Yule he made a remark that subsequently gained epigrammatical currency. An associate, Brownlee, had consulted him about a possible adverse criticism he proposed to make on some of Pearson's recent work. "I have contented myself with warning Brownlee," wrote Greenwood, "that before he kicks Karl's arse, he had better make sure that the said arse is where he thinks it is."

Early in August he opened a discussion on epidemic diseases at an International Medical Congress held in London, and then set off on a cycling tour with Arthur Bacot to Keswick via Peterborough, Lincoln, York, Ripon and Jervaux Abbey.

3.13. Swine Fever

Early in autumn 1913 Pearson rather haughtily passed over to Greenwood an investigation into Swine Fever. He had, he said, neither the time nor the staff to do it himself. Greenwood was suspicious though he heard from round about sources that Pearson believed himself to be ill. The Board of Agriculture had recently prescribed isolation and destruction of pigs suffering from fever. The pig breeders were reluctant to destroy their infected stock and wondered if the fever could be countered by the use of inoculation practised on the continent. A statistician was required to investigate the efficacy of inoculation. In September Greenwood received a visit from HR Beeton the President of the Pig Breeder's Association. Beeton, a pioneer of public electricity supply, was then in wealthy and energetic retirement. He was a man of wide interests and in his earlier years men like Edgworth, Shaw, Fowell and Wicksteed used to meet at his house for discussions on economic problems.

Greenwood accepted the investigation, worked hard on it, but through lack of data, his findings were inconclusive. Nevertheless he struck up a friendship with Beeton that lasted until the latter's death in 1934.

3.14. Bawdiness and the Birth Rate Committee

In November 1913 he got involved in an investigation into birth rate statistics. Among other things those who calculated birth rates were curious to know roughly how many births were prevented by the use of contraceptives. On November 8th Greenwood wrote to Yule: "Yesterday I sat among the great and holy and we examined a son of Belial who supplied the public with soluble pessaries. He reckons that on the assumption of two pokes a week, he enables 40,000 married couples per annum to avoid increasing the birth rate. Herr Rechnungsrat, this is good business. Multiply 40,000 by 104 and we reach four million pessaries. They are vended at a minimum rate of 1/9d per dozen. Suppose the devil supplies the retailer at a bob, and that his cost of production is a tanner (quinine is *very* cheap nowadays), then he pouches over £8,000. He doesn't advertise he says.

Let us firmly dissociate ourselves from correlation and such like baubles and start a new firm. Yule and Greenwood, Cambridge and Loughton; … Sole manufacturers of the "Eugenist" soluble pessary. 1 dozen forwarded in plain envelope with full directions and copies proving the importance of family restrictions by Karl Pearson and other eminent scientists, on receipt of PO for 2/- … I enclose a specimen ad. Please have it set up by the CUP in small pica and distribute to all married residents …"

The advertisement ran as follows:

"Have you any children? Do you know what the great scientist Karl Pearson says? He says "The whole Future Generation is produced by less than half the existing generation!" Is this fair to you? Why should you overwork like this? Why do you stand it? Do you know what Sir James Crichton Browne and other eminent doctors say? They say that the rapid multiplication of the unfit is one of the most alarming signs of the times! Hadn't you better consult experts who have spent their lives studying this problem? Why not do it now? Send a stamped and addressed envelope, together with a PO for one shilling to cover expenses, to Messrs. Yule, Greenwood and Co., c/o Master of St. John's College, Cambridge."

Yule commented later that he had had to hide this letter lest his servant should see it and ask him for advice!

3.15. Threat to the Lister Institute

Suddenly the pleasant tempo at the Lister Institute was shattered. Turning up there on January 1st 1914 Greenwood found that the whole future of the place was in the melting pot. "It seems," he wrote to Yule on the 3rd, "that Martin – the Director – has persuaded himself that the whole show ought to be handed over to the nation, building, income, endowment and all to form the nucleus of a Kaiserliches Gesundheiteampt … Iveagh – the chief benefactor – has been interviewed and appears to agree and the matter is to be discussed by the Governing Body".

What had happened was this. Under the National Insurance Act of 1911 there was a provision for the setting up of a new department for medical research. As the Lister Institute was already engaged on the kind of work envisaged it seemed sensible for it to be used as the nucleus for the new establishment. The Governing Body had been approached by the National Insurance Commissioners and had appreciated that many benefits could derive from Government support. They believed that it would be in the best interest of medical science to hand the establishment over to the State. Nor was Lord Iveagh unwilling. Although he may have regretted that his large endowments would pass into government hands he seems to have regarded the Institute as something of a failure. "What Iveagh wanted," Greenwood commented later, "was quick returns, a cure for cancer and the rest."

Greenwood was thoroughly shaken by the proposal and poured out his alarm to Yule. "How all my so-called Socialism scabbed-off when Martin asked me my views. I loathe this idea and told him so …. I can see this National Institute the damned nightmare of a megalomaniac: departments and sub-departments, reports, officials, salaries, holiday rules, KCBs, every damned thing except an immortal soul …. None of your damned hole in the corner two rooms …. But a DEPARTMENT with a real lord high statistician and a staff who shall survey the whole problem of national disease and pronounce thereon ….

God damn and blast and confound the whole crew. My ideal of science is the definition of the Catholic Church in the 19th Article of religion, a "congregation of faithful men", that is, all people honestly doing their best to reach the truth and willing to credit their fellows with the same intention …."

This was emotional and rather mixed-up stuff but it had been aroused by the fact that Greenwood had read the proposed schedule of management. There was to be a Statistical Department with an organisation and programme "which might have been drawn up, perhaps was drawn-up," by Pearson himself." Greenwood's panic was caused by the thought that Pearson would be invited to become the "lord high statistician", would decline and his nominee be appointed. That Pearson would nominate him seemed most improbable, and he would lose his present position of control.

He therefore determined to intrigue against the appointment of a Pearson nominee in the event of the major scheme going ahead. He asked Yule if he would enter the lists for the position of "lord high statistician". Yule was sympathetic. He agreed that to turn the Lister Institute into a government department would double the cost and halve the output of good work, but he declined. Then he confided something about which Greenwood – for all their intimacy – seems to have been totally unaware. He was not the bachelor Greenwood had supposed. He had once been married. His wife had left him, run him into debt and harassed him miserably through the courts. The worry had aged him and affected his memory. The bottom had dropped out of his world and he had lost any ambition he may once have had, He had grown fond of the life at Cambridge and had no wish to become a "lord high statistician". Greenwood was the obvious choice, he said, and would say so officially if he were asked.

Greenwood was shocked into humility by these revelations in contrast to his own worries and condolingly replied in phrases of Johnsonian cadence. None the less he gave the appearance of being at his wits end and rather than submit to being placed under a nominee of Pearson, spoke of throwing in his hand and abandoning research altogether. He wrote about buying a share in a medical practice. Yule hardly knew whether to take

him seriously, and having tried to discourage him from buying a medical practice, offered to help him with a loan if he were really serious.

So the matter rested in the early months of 1914 until the Governing Body of the Lister Institute would be called upon to make its decision later in the year. Meanwhile there was time for Greenwood to muster the opposition.

His peace of mind was further disturbed by the imminent departure of Bacot on a mission to Africa, where he was to take part in the work of the Yellow Fever Commission at Freetown in Sierra Leone.

Troubles shared with an intimate friend were troubles much diminished but soon there would be no Bacot.

In May he and Rosa took their bicycles and stayed with Yule at Cambridge. Later in the summer they proposed to spend their holidays in Germany. Bacot's suggestion made a year before when they stayed together at Keswick, that the country might be drifting into war with Germany still seemed quite unreal. At the end of June came news of the murders at Sarajevo, but they caused barely a ripple on the minds of the researchers. In July Bacot sailed for Africa, and as late as July 30th Yule was under the impression that the Greenwoods were still going to Germany. Pearson himself was actually in Germany, and just managed to get back to England on August 4th! The Greenwoods changed their minds and went to Southwold instead.

4. First World War, Army Service, and the Ministry of Munitions (1914 – 1918)

4.1. Outbreak of War

The first impact of the 1914 war was slow to be felt by the "outsiders". It was a job for the professionals, and the professionals at first meant to keep it that way. For Rosa Greenwood the war became an immediate tragedy and she was soon to experience the hurtful things that people would be saying about the Germans.

Meanwhile opposition was growing to the Governing Body's proposal to hand over the Lister Institute to the nation. The Chairman, Sir John Rose Bradford, had now been won over to the opposition and he resigned. A special meeting at which the proposals would be put to a final vote was called for Wednesday November 18th. Greenwood's anxieties mounted. On the one hand he was querying the Institute's Articles of Association in an attempt to discover interpretations favourable to the opposition; on the other he was contemplating joining the Army if the proposals were carried.

On the evening of the 17th Yule sent him a comforting letter. He didn't think that men like Greenwood, with wife and children dependent on them, ought to enlist except as a last line of defence or unless they felt they *must* to save their souls. And yet, if he did enlist, it might not be nearly as irksome as one supposed to be ordered about by men in some respects ones inferiors, provided they really knew their jobs. Yule had recently joined "a kind of Crock's Squad for MAs" to keep up his spirits. "I find drilling under a sergeant," he confided, "great fun, as he knows his job thoroughly and I do not, though I pity him, as he must find us a sorry incompetent lot."

When on November 18th the motion of surrender to the State was put, it was lost, 32 votes in favour, 39 Against, Greenwood's postcard of triumph to Yule has not survived, but to judge from Yule's reply it was a triumph alloyed with difficulties. The members of the Institute were now firmly divided in their views, and were in danger of losing some of their scientific purity. There was a feeling of anti-climax. Nothing was going to happen after all. And gradually there seeped into the vacuum the realisation that the country was engaged in a major war and that perhaps there were scientific horizons far beyond those dreamed of by the congregation of faithful researchers.

During this period Greenwood and his statistical assistants were engaged in studying the relationship between Cancer and Diabetes death rates, methods of Index Correlation, the changes in recorded mortality from cancer, and the somewhat esoteric problem of determining the size of families and the distribution of characteristics in order of birth

from samples taken through members of their sibships. Meanwhile some 85,000 of their fellow countrymen had suffered from the savageries of war. Even so, wounds could be inflicted nearer home, and looking back on the situation from half a century later, there is just a hint that from Greenwood's camp in the Lister Institute, the school of Karl Pearson was a much more apparent enemy than the German High Command.

There was constant bickering between them. When Greenwood delivered a paper to the Royal Society of Medicine on *Changes in Mortality from Cancer*, he cast some doubt on conclusions that had been reached by Pearson. Dr David Heron, Pearson's assistant, promptly attacked him on grounds where the "Karlovignians" – as Yule called them – knew it would be most painful – his lack of mathematical training. Pearson himself published a stern *Correction of a mis-statement by Mr. Major Greenwood, Junior*. There was spitefulness on both sides, but on the whole Pearson came out of it worst because as the greater man he had less call to be spiteful at all.

4.2. Army Service

The year 1915 opened with a zeppelin scare and an all round tightening of the strains of war. It was becoming evident that even the purest of scientific researchers would not be left much longer to pursue their own disinterested enquiries. Yet for a while there was a pause. Greenwood and Yule went on meeting at the Statistical Society and dining at the Savile Club where they both had a partiality for sweet sherry. Greenwood was still engaged on the investigation into swine fever for old Mr Beeton and occasionally spent the week end at his house at Checkendon near Reading. His venture into swine fever, however, was not viewed with universal approval by the Board of Agriculture, some of whose junior personnel were puzzled to understand how a knowledge of the mathematical handling of data could have any bearing on a subject which seemed to be wholly veterinary. One of them, in Yule's presence, having glanced at Greenwood's report, commented angrily, "Damned impudence, you know, for a layman to meddle with the subject."

Meanwhile the staff of the Lister Institute were drifting into War jobs. Greenwood became restive, and was further jolted into uneasiness by Yule's sudden acceptance of a job in the Contracts Department of the War Office. In July Greenwood reported a breakdown in his work, and began searching for an Army job.

It was not necessary to search very far. Practically next door to the Lister Institute lay the Duke of York's Headquarters. Attached to this establishment were the 1st and 2nd London Sanitary Companies, forming part of the Royal Army Medical Corps. The Official History of these Companies reports with pride that in the early days of the war the quality of volunteer recruits, was extremely good, sanitary inspectors, school teachers, architects, surveyors, plumbers and so forth. The voluntary application of a fully qualified medical man must have even further gratified them. By the end of July Greenwood had been accepted for a Commission in the 1st Sanitary Company.

His military career began with a course of instruction on *The Sanitation of War,* following by routine inspections of army drains and water supplies. Of his detailed affairs we have been able to find out little, except that he was away from home five nights a week, a good deal at Tidworth Pennings on Salisbury Plain, and learnt to ride a horse. He grew what Yule called an "interquartile" moustache. He was gazetted Captain on February 9th 1916.

The London Sanitary Companies grew in importance as the war continued and their personnel were used to build up Sanitary Companies that were eventually attached to each Divisional Headquarters. Their duties consisted mainly of camp hygiene, the disposal of sewage and camp refuse, and the purification of drinking water. Under conditions of trench warfare these matters presented enormous difficulties. They also provided scope for scientific research though Greenwood was not long enough with the Sanitary Companies to participate in much original work.

He seems to have enjoyed the open air life and was able to resume his home affairs at week ends. Home life, however, was beginning to present difficulties. Rosa was suffering

from minor anti-Germanism, and his step-mother was assuming an awkward attitude towards her. This underlined her foreign-ness and made her feel more lonely in the country of her adoption. There was trouble too over Arthur Bacot's sister Alice. He was still away in Africa, and she, living alone in their house at Loughton, had developed nervous troubles and nobody knew quite what to do. Fortunately a friend of Greenwood's from the London Hospital days, Millais Culpin, and his wife had moved into the district, and between them they made arrangements for her care.

During his week ends at home Greenwood took Rosa and Mrs. Culpin for walks *round* the forest, not through it, as both the ladies were wheeling prams. In the evenings he pottered around with statistical problems posed by Yule or left unfinished from the Lister Institute days. Occasionally he was troubled by soldiers on leave mistakenly applying to him as an Army Doctor for sickness certificates to enable them to extend their leave. Neither side in these transactions emerged entirely happy. Sometimes Yule came down for the week end, and usually in the summer months there was the ritual of early morning bathing in a pond in the forest, followed after breakfast by a mid-morning walk. Then back again on Monday to Army Sanitation. He was not, however, to be left much longer inspecting Army drains.

4.3. Ministry of Munitions

The munitions crisis of 1915 had resulted in the creation of the Ministry of Munitions with Lloyd George as Minister. In September of that year the Minister appointed a Committee to advise on questions affecting the health of munitions workers. Under the Chairmanship of Sir George Newman, the Committee included Greenwood's earlier patron, Leonard Hill, Sir Walter Fletcher (Secretary of the Medical Research Committee) and EL Collis (Medical Officer, Factory Department, Home Office). Scientific investigators were urgently needed. Hill immediately thought of Greenwood and approached Collis who readily agreed. As a result an application was put through to the War Office for the loan of Greenwood for investigational work. In February 1916 the War Office seconded Greenwood on a six monthly basis to the Ministry of Munitions.

Greenwood seems not to have been fully aware of what was going on, and to have been puzzled when the order came for his transfer to the staff of the Health of Munitions Workers' Committee. He also appears to have been annoyed. The fate he had feared at the Lister Institute seemed at last to have overtaken him. Both he and Yule suspected that his special statistical skills were to be employed in supporting some dubious political decisions. Otherwise, Yule commented, "they would never have dreamt of asking in a bloomin' expert."

These ominous forebodings were misplaced. As Collis recollected many years later, when they applied for Greenwood's services, the Committee hadn't the least idea how they could use them. When Greenwood arrived at the Committee's headquarters there was absolutely nothing for him to do. His spirits, already low, fell to zero. But there were compensations, to be living at home again, being in London, wearing civilian clothes and resuming his personal contacts. He and Yule discussed a lot of mathematics together. Yule was pondering over the chi-squared test and Greenwood was studying the works of the Reverend Thomas Bayes (c 1702 – 1796). Bayes' notions on matters of probability – gave them both a great deal of trouble. After a prolonged attempt at analysis, Yule, thoroughly exasperated, concluded some observations to Greenwood on the subject with the following verses;

Are you sick of the turbulent nations
Yet not really inclined for a laze?
Take samples of *n* observations
And then take the standpoint of Bayes.

Try his methods first this way and then t'other,
Try them poss – and impossible ways,
Don't try your invention to smother
But try ALL the methods of Bayes.

Your ideas once so clear will grow fuddled,

You'll get many results that amaze,
In the end you'll be thoroughly muddled
By that blasted old bugger called Bayes.

By Edgeworth I'm frequently bunkered,
And put in a hell of a haze,
But I reel to and from like a drunkard
Under blows from the Reverend Bayes.

L'Envoi

God bless for the sake of his *dear* Son,
This humble petitioner prays,
The King, Greenwood and even Karl Pearson,
But damn both the Kaiser and Bayes.

(FY Edgeworth (1845 – 1926) was an economist and statistician, President of the Royal Statistical Society 1912 – 14; interested in the theory of probability, and hence the association with Bayes).

4.4. Liquor Control

While Greenwood's new department in the Ministry of Munitions was organising itself to cope with the Welfare problems of the factories, he suddenly found himself invited to his first seat on an important Government Committee.

In May 1915 a Board of Liquor Control had been established under the Defence of the Realm Act to cut down what Lloyd George had called the "lure of alcohol" which was increasingly hampering the war effort. Its first duties were to reduce the number of places at which drink could be bought and restrict the hours during which it could be sold. Thereafter the Board issued a stream of regulations that caused a social revolution in the British drinking habits that endure to this day. The Board, however, was hampered by an

absence of exact information about the physiological effects of alcohol. Thus, for example, some “experts” believed that alcohol in beer was absorbed faster into the bloodstream than alcohol in spirits; other “experts” held the opposite view. There were equally divergent views on the qualities of drunkenness caused by beer and spirits. To those entrusted with the duty of making regulations for the control of drink and its side effects, it was important to know the truth. If, indeed, beer made a man drunk quicker than the equivalent amount of alcohol in spirits, then perhaps it might be as well to discourage excessive beer drinking by raising its price, or reducing its alcoholic content.

To overcome these uncertainties, in November 1916, the Board appointed an advisory committee to investigate the physiological effects of alcohol in its various forms.

The Chairman was Lord D’Abernon. The scientific members were Prof AR Cushny MD, FRS, Dr DH Dale FRS, Dr M Greenwood, Dr W McDougall FRS, Dr FW Mott FRS, Sir George Newman KCB, MD, Prof CS Sherrington MD, FRS, and Dr WC Sullivan.

This was very distinguished company, and it seems likely that Greenwood’s appointment was due to Sir George Newman for the four reasons that he was already on Newman’s staff, was the only statistician among them, had already been involved in alcohol investigations with Pearson in 1910 / 11, and had applied statistical techniques to physiological problems under Leonard Hill, who sat with Newman on the Ministry’s Committee.

The Advisory Committee’s first job was to draw up an account of contemporary knowledge with regard to the physiological action of alcohol, which was eventually published in 1918 under the title *Alcohol: Its action on the Human Organism*. Next it initiated a number of investigations into specific problems relating to alcohol, which were subsequently published by the Medical Research Council. One of its findings has passed into popular drinking law: that milk in the stomach lessens the likelihood of intoxication.

Greenwood's part in these researches was largely masked by the physiological fact finder, notably Dr E Mellanby, but he appears to have applied a vast amount of mathematics to the various problems.

In May 1917 the Welfare Branch of the Ministry of Munitions was enlarged and it was decided to establish a Medico-Statistical Section for the specialist handling of statistical data that arose from the various welfare investigations. EL Collis, now Professor of Preventive Medicine at Cardiff, had been appointed Director of the Welfare Branch, and he put Greenwood in charge of the new section, and asked him to organise a statistical laboratory. He got together a small staff, consisting notably of Miss Hilda Woods, Miss Cecily Thompson and Miss Wilcox, and awaited an in-pouring of data. It was not long in coming. Not only was there a munitions crisis, but it was beginning to be aggravated by a growing food shortage caused by enemy submarine action.

The first investigational material dealt with nutrition. Food rationing had already been introduced in 1916, but when the enemy began unrestricted submarine sinkings early in 1917 there was a food crisis. Rationing had to be extended, and the government virtually took control of all the food services. As with liquor control, the authorities were continually hampered by an absence of exact knowledge on the amount of food that was actually being consumed, and how much of this consumption was necessary. It was believed, with some justification, that customary food usage was extremely wasteful. Thus, for example, bread was often thrown away when it became stale, yet the amount of bread so discarded represented a considerable tonnage in shipping space for which men risked their lives.

The Ministry investigation was only concerned with Munition workers, but it had ample scope. Questionnaires were drawn up and sent to the managers of munitions hostels to find out what and how much the workers were actually eating. Greenwood's department then drew up an elaborate report on the diets consumed by the largest sample of industrial workers ever taken.

At first hostel and canteen managers were sceptical about the practical use that could be made of this information, but when it became available, they changed their minds, and the Ministry was able to effect on the one hand economies in total foodstuffs and on the other, an improvement in variety. The information further proved of great value to the Food Section of the Ministry when it was set up in 1918.

Work on dietaries then developed along more complex lines, and the significance of calories was introduced to the British public. Investigations were carried out to ascertain the amount of calories needed to perform particular physical work, with a view to discriminatory rationing according to physical need. One of the enquiries on which Greenwood was engaged was to determine the most calorifically economic speed of marching. It proved to be 88.38 yards a minute, almost three miles an hour, though the army seems to have stuck to its standard speed of 120 yards a minute.

4.5. Labour Wastage

The next major investigation dealt with absenteeism and labour wastage among some 40,000 women munition workers. If it never claimed to have discovered anything hitherto unknown to the industrial employer, it did, in a very clear way, demonstrate the enormous losses to production caused by casual wastage. The report was published by the Medical Research Committee, *Special Report No. 16*, in 1918, and was resurrected some twenty years later for guidance in another war. Since the pioneer days of 1917, Labour Wastage has become a standard matter of concern to all large scale employers, and in a sense their interest may be said to have stemmed from the revelations made by the Ministry of Munitions. Greenwood's report consisted of an impeccable introductory essay on the subject, followed by 80 pages of numerical tables, concluding with a *Note on Errors of Sampling* containing an impressive display of algebra which must have been incomprehensible to most of the members of the vetting committee. It also contained a graceful tribute to Karl Pearson, and there is some evidence that this did not go un-noticed.

Greenwood's Statistical Section had many calls on its time, and his and the names of his collaborators can be traced through numerous official publications on health of the period. With Dr AE Tebb it conducted an enquiry into the prevalence of tuberculosis among munition workers and a study of the influenza pandemic of 1918. Other enquiries concerned such matters as ventilation, lighting, seats for workers and general work organisation. It organised a medico-statistical department for the Royal Air Force and was frequently consulted by the Local Government Board.

The fact that his report dealt mainly with women underlines one of the primary purposes behind the creation of the Ministry's Welfare Branch. For the first time enormous numbers of women were being drafted into industrial jobs formerly performed by men. Fears were entertained that this would be damaging to their health. As events proved, this was not so, and as Greenwood remarked in the book he eventually wrote with Collis on the *Health of the Industrial Worker* (1921) the effect of industry on women was far less important than the effect of women on industry. In trying to protect women from the hazards of industry, the Government awoke more fully to the fact that men too needed to be protected, and much of the welfare legislation that followed the conclusion of the war stemmed from action originally taken in the interests of women employees.

Another investigation in which he was involved dealt with the frequency of industrial accidents. He seems to have been the first to draw attention to the fact that a majority of accidents were caused by a minority of workers, and that the distribution was by no means as random as the word "accident" generally connoted. This gave rise to a theory of accident-proneness, that some workers, perhaps on account of some psychological defect, were more prone to accidents than the average worker. Everyone recognised that some workers were more careless than others, but this proneness to accidents seemed to go further than this and to involve more complex factors. It was a problem, moreover, particularly fascinating to a statistician because in the analysis of the data it was necessary to recognise where the reported accidents exceeded the distribution of chance. Although his report on the subject was published in 1919 he toyed with the problem in a

mathematical way for several years and regarded it as one of his most useful contributions to the subject of industrial accidents.

5. The Ministry of Health (1919 – 1927)

5.1. Post War Uncertainties

When the war ended in November 1918 Greenwood expected that the activities of the various Committees with which he was involved would be diminished and spent several months anxiously prospecting for the future. He seems not to have realised the enormous possibilities for extension in peace time of the socially desirable work begun under the stimulus of war. His ambition was for a University post but there were few available and all poorly paid. In January 1919 he was contemplating a residential post at Cambridge, but the salary of £250 per year was too small. He did, however, agree to give a series of lectures there on Epidemiology which, as Yule pointed out, would at least give him an entrée to the University.

Yet all the while the scope for government employment was growing. The various Public Health Services were so ill-co-ordinated that the Government was now proposing to amalgamate them into a new Ministry of Health. Instead of seeing an opportunity here, Greenwood resolutely set his face against the new Ministry. He was, it seems, suffering from a kind of emotional immaturity, and experiencing again his feeling of revolt when the future of the Lister Institute had been threatened by Government intervention in 1914. His feelings of inferiority seem to have risen against him, and blinded him to the fact that for the past few years he had mixed on equal terms with scientific men of considerable influence, who had relied on him for the scientific presentation of their work. Instead of using his opportunities, he fell back on the belief that if he were unable to get a university post, he could always go back to the Lister Institute.

In March 1919 however, doubts began to grow that the Lister Institute could find a place for him. The future seemed very unsettled. He began to worry and suffer from bouts of insomnia. At last he concluded there was no alternative to the new Ministry and signified

his willingness to be found a place. It seems odd that he should have been reluctant when the auguries were so favourable. He was already known to and had worked with many of the men destined for high places in the new Ministry. Sir George Newman, formerly his Chief on the Old Health of Munitions Workers Committee had already been appointed Chief Medical Officer. Greenwood approached him in May and Sir George gave him an assurance of congenial employment.

In July he delivered his first course of lectures on Epidemiology at the Cambridge Medical School. Yule was away on holiday and Greenwood borrowed his rooms at St. John's College. The impending change of work unsettled him, and he was nervous and lonely, and the preposterous, windowless medical lecture theatre was hardly a comforting place in which to meet an unfamiliar audience. Depression set in. Yule had hoped that the quiet of his rooms would at least give him a rest. There was, he said, a good deal of restful literature on the shelves. "Can you stand Jane Austen?" he asked. "I believe you are unfortunately one of the people who can't." Perhaps it was this period that Greenwood began to cultivate a fondness for the gentle writer.

The Cambridge lectures were a success, though the nervous lecturer hardly thought so at the time. When they were over he took his family on holiday to Southwold in Suffolk. In August he was released from the Army and the Ministry of Munitions and returned temporarily to the Lister Institute pending a definite appointment with the Ministry of Health.

5.2. At the Ministry of Health

The first Minister of Health was Christopher Addison, a medical man who had become MP for Shoreditch and was an ardent supporter of Lloyd George. When Lloyd George became Prime Minister, he had appointed Addison to take his place as Minister of Munitions.

The work of the Ministry was divided into six sections. One of these was to deal with General Health and Epidemiology. Greenwood was appointed to this section as Medical

Officer in charge of statistical work, at a salary range of £700 - £1,200. His senior was Dr GS Buchanan (1896 – 1936) who had long been in the public health service and was to become a great promoter of international health.

Although Greenwood's new post seemed to provide enormous scope for his interests, he accepted it reluctantly, and continued to look about for alternative jobs. As late as July 1920 we find him contemplating a post with a New York business house about which tantalisingly little evidence has survived. His heart was set on pure research and the probability of largely routine statistical surveys with the Ministry was not alluring.

As it happened, initially at any rate, the new job made relatively little difference to his occupation or way of life. The new Ministry was in the throes of organisation and nobody had any special work for him to do. During this period he continued to work for the Medical Research Committee, on whose Statistical Committee he remained, much as he had done while employed by the Ministry of Munitions. For a time he continued to occupy the Committee's dingy rooms in Northumberland Street off the Strand.

Growing tired of the small sunless rooms he devised a way of escape. In 1914 Leonard Hill had been appointed Director of Applied Physiology at the National Institute of Medical Research at Hampstead, one of the Medical Research Committee's establishments. This was a fine airy building not far from the Heath. With Hill's connivance he arranged for the Statistical Committee to be provided with accommodation in Hill's department. It was an easy matter to convince his Chief, Sir George Newman, the Chief Medical Officer, that both the Ministry's and the Board's statistical work could better be carried out at Hampstead than in the Ministry's offices at Whitehall or the cramped little rooms in Northumberland Street. Thus began Greenwood's association with the Research Institute at Hampstead.

The first major statistical work for the Ministry was a survey of the influenza pandemic of 1918 / 19. This was possibly the largest and most deadly scourge that had been felt in Europe since the Black Death. The peak of deaths in England had occurred early in

November 1918, almost exactly coinciding with the Armistice, and although the pandemic killed more people than the Great War itself, and in a shorter time, the general relief at the ending of hostilities seems to have prevented it from sinking into the common memory.

According to the Ministry's First Report, covering the years 1919 / 20 Greenwood's efforts had made it possible to obtain statistical studies of health and sickness of an entirely new kind. Although he had been mainly engaged on the influenza studies he had begun re-organising the collection of statistical data relating to infectious disease, and had made a preliminary report on the epidemiology of tuberculosis.

In the Ministry's Second Annual Report we find the following statement: "From its foundation the Ministry has appreciated the importance of statistical science as an aid to sound medical administration and as an instrument of research. To Dr. Major Greenwood, the medical statistical officer, have been assigned the duties of a) critically examining the validity of inferences sought to be drawn from statistical returns reaching the medical staff, b) reporting to the Senior Medical Officer for Epidemiology and Medical Intelligence on mortality and morbidity returns with a view to obtaining early warning of local epidemics, and c) conducting statistical research."

Opinions had changed since Almroth Wright's onslaught on the biometrical busy-bodies before the war. "Mr" Major Greenwood was now an officially appointed watchdog to vet the inferences sought to be drawn from various data by the medical experts".

Although in 1920 he was mainly engaged on the influenza work, he did a laborious investigation into the relationship between pneumonia and bronchitis and meteorological conditions. He began an enquiry into the changes in the death rates in various parts of the country. All the while he found his services more and more in demand as the Ministry's machinery got into action and produced ever increasing quantities of statistical data. He became a kind of statistical maid-of-all-work, and among other things, found himself writing substantial parts of the Chief Medical Officer's Reports.

At this point it is worth recalling Greenwood's special admiration for William Farr (1807 – 1883) who had been appointed "compiler of abstracts" in the new General Register Office in 1839. Farr had a curious turn of imagination and Greenwood used to trace his hand in the odd and ingenious comments embodied in the Reports of the Registrar-General, Major George Graham, during Farr's period, and which he thought could only have been written by Farr. Anyone with a knowledge of the styles of Sir George Newman and Major Greenwood might find it an amusing occupation tracing the latter's hand in the former's reports.

5.3. Reconciliation with Pearson

In July 1920 Greenwood reopened correspondence with Pearson. At first it was formal and full of mathematics, with Pearson employing his more distant style of address: "Dear Dr. Greenwood …. I am, yours sincerely, Karl Pearson." In the middle of August Greenwood actually wrote an apology for their past misunderstandings, which Pearson accepted in the most generous terms, after which their correspondence became warm and friendly.

What prompted the reconciliation, and Pearson must have known this from the start, was Greenwood's wish to secure a University appointment. Ever since the end of the war Pearson had been in difficulties over the maintenance of his department at University College. Owing to the rise in the cost of living, or alternatively to the falling value of money, the University authorities had been unable to maintain Pearson's Eugenics Department in the state to which he thought he was entitled. Various appeals for more money had gone out and Greenwood, sensing an opportunity for resuming his connections with Pearson, offered to use his influence with Sir Walter Fletcher of the Medical Research Committee with a view to getting some kind of subsidy for Pearson's department, or getting the Committee to lend Pearson one of its research workers.

Pearson was cautious about this suggestion, and during the correspondence he let slip an interesting piece of information. The London County Council had been persuaded into

supporting Pearson's department by paying for the appointment of a Medical Statistician. He added, moreover, that a highly competent appointee was in mind.

This was exactly the kind of job Greenwood was anxious to secure, and his apology to Pearson seems to have dated from the time when he first got wind of it. Hesitant of applying for the job directly, he angled for more information which Pearson was evasive in giving. Greenwood tried a flank approach. As Statistician to the Lister Institute, which had been recognised as a teaching school of the University of London since 1905, he had occasionally given lectures for the University which gave him the status of Honorary Reader in Statistics there. No longer a member of the Institute, he sounded Pearson on the possibility of having his Readership made into a personal appointment. This was done in November.

Meanwhile Greenwood and Yule were consumed with curiosity to know more about Pearson's plans and the identity of the proposed appointee. Yule wondered if Pearson intended to train up the appointee to take his place as Galton Professor of Eugenics when he retired, and when Greenwood seriously asked Yule if *he* would take the Chair if it were offered, Yule replied, "I am not a eugenist, and I am not in the least keenly interested in eugenics; and it really would be damned dishonest to think of the post." Greenwood's question rather suggests that he had vaguely thought of himself in the Chair, but Yule's answer was equally true if applied to himself. Later in the summer Pearson asked him outright if he wished to apply for the post of Medical Statistician. They had an intimate discussion about this at Pearson's "Old School House" at Coldharbour, near Dorking, as a result of which Greenwood withdrew any thoughts he might have had on candidature, Finally, Dr Percy Stocks was appointed to the position.

Whatever the outcome of the jockeying for places, the most important thing was a resumption of the old cordiality between Pearson and Greenwood, signified by Pearson's return in his letter to "My dear Greenwood".

5.4. First Chairmanship and Leon Isserlis

In 1920 the Medical Research Committee was incorporated as the Medical Research Council under a Committee of the Privy Council, with funds supplied by Parliament. It established an Industrial Fatigue Research Board with several research committees. As the Ministry of Health's Statistician Greenwood was invited to become Chairman of the Board's Industrial Health Statistics Committee. It was a particularly important committee in that it exercised some sort of supervision over the statistical conclusions drawn up by the other committees. The Chairmanship seems to have been offered to Pearson who declined it on account of pressure of work.

The other members were John Brownlee (Director of Statistics, Medical Research Council), EL Collis (Professor of Preventive Medicine, Cardiff University), A Henry (Deputy Government Actuary), G Udny Yule (University Lecturer on Statistics, Cambridge), Leon Isserlis (Statistician, Chamber of Shipping) and Miss EC Allen (Secretary). Within a short time Leonard Hill (Director of Applied Physiology, Medical Research Council) and A. S. Macnalty (Medical Officer, Ministry of Health) were added, and E Lewis Faning was made Secretary.

Greenwood's hand can be seen in the formation of this committee. Hill, Collis, Yule and Isserlis were old friends, Macnalty and Brownlee were former colleagues, the latter one of Pearson's associates, and E Lewis Faning was a distant relative of Greenwood. It was a committee utterly to Greenwood's liking and he remained its Chairman.

As Greenwood's personal friends, some account has already been given of Hill, Collis and Yule. Leon Isserlis was a newcomer.

Leon Isserlis, a year younger than Greenwood, was a Russian Jew. His father had died young, and in 1892, when the Russians were persecuting the Jews, his mother had brought him and the rest of the family over to England. They stayed with a cousin who had a chemist's shop in Whitechapel and lived over the shop. Isserlis went to the City of London School where he won a scholarship to Christ's College, Cambridge. He took his BA in 1903 and was 18th wrangler his year – a high distinction in mathematics now

differently classified. In 1904 he was appointed head of the mathematics department at the West Ham Technical Institute. He attended lectures by Karl Pearson, became acquainted with the new biometrical ideas and casually got to know Greenwood. He was so strong an admirer of Pearson that he named his second son after him, Karl Pearson Isserlis, though everyone called him Peter.

Like Pearson, Isserlis was a radical and while still at Cambridge had joined the Fabian Society. Shortly before the 1914 war he had moved his family to Loughton which was a convenient distance from West Ham. In those days he was poorly paid and used frequently to cycle to and from his work, an undertaking he sometimes found hazardous during the war years when policemen were suspicious of Russian Jews silently pedalling through the night. He rented a tiny cottage on Baldwin's Hill on high ground overlooking Epping Forest. Close by another cottage was occupied by Jacob Epstein, already the centre of artistic controversy.

During his forest walks Isserlis soon ran into Greenwood and Bacot and they became fast friends. Although so apparently disparate they had much in common quite apart from a love of dogs and the forest. They were all Fabians, there was a Pearsonian link between them and just as Bacot had longed to find a niche for himself, so did Isserlis. Statistics became a bond between Greenwood and Isserlis, and while Isserlis helped Greenwood with his mathematics, Greenwood fostered Isserlis's statistical interests.

Early in 1920 the Chamber of Shipping, an association of British ship-owners, decided to appoint a statistician and Isserlis was encouraged by his friends to apply for the position. About this the following story is told. Isserlis's chances were regarded as small. He had no personal influence in the face of other candidates who might well have and there was prejudice against a person of foreign origin, especially Russian. He collected what testimonials he could, sent them in with his application and hoped for the best. Among them was a glowing recommendation from Greenwood written on Ministry of Health notepaper. When the selection committee came to inspect the testimonials, so the story runs, it mistook the signature for that of H Greenwood, the Rt Hon Sir Hamar

Greenwood, a noted politician lately Under Secretary for Home Affairs and currently Secretary for Overseas Trade. The story sounds improbable but in the event Isserlis was appointed. Improbable or not, a few years later Greenwood was himself the subject of a misunderstanding. When Arthur Greenwood MP was appointed Parliamentary Secretary to the Ministry of Health in 1924, Greenwood's photograph appeared with the announcement in the press.

Isserlis, by now an MA and a DSc, became Vice Chairman of the Industrial Health Statistics Committee, and Greenwood explained that as he himself was not a *pukka* mathematician it was important that the committee should have the part time service of somebody who was.

Many years later Isserlis reciprocated Greenwood's help in the matter of his appointment to the Chamber of Shipping by supporting the appointment of his elder son to a position with the Chamber.

There is an odd little tailpiece to these recollections of Leon Isserlis in the early 1920s supported by no more than family gossip. From this it would appear that Isserlis's mathematical skills had not passed unobserved in Russia and that he had received an invitation from Lenin to return to Russia and work for the re-organisation of the new state.

5.5. *The Health of the Industrial Worker*

One of the by-products of Greenwood's industrial health researches during the final years of the war was his collaboration with Collis in the preparation of a large book entitled *The Health of the Industrial Worker.*

The book originated in a request from the publishers, J & A Churchill, to Collis – who was then Home Office Medical Inspector of Factories – for a book on Industrial Medicine. Collis said afterwards, "I think they had in mind Industrial Disease, but I said Industrial Health seemed to me a better thing. Then I asked Greenwood to collaborate as

he had been so active just then in the whole subject. Except for any statistical angle, which was his speciality, we did not take any part to either of us. While he certainly contributed to the historical side, my recollection is that I myself wrote most of that part. At every stage each saw the other's MSS, and altered or added as seemed advisable. It was just collaboration."

"Without being a best seller, the book just about covered the publisher's expenses; but by the time the first edition was sold out much time had passed and an entire rewrite would have been needed rather than a second edition. At any rate it was not contemplated, especially as we were neither of us any longer actively engaged upon Industrial Health problems…"

"I only know that we got some small contributions from sales during the next ten or twelve years. It was rather an expensive book (30/-) for those times for individuals to acquire. It can never have reached the book-stalls, only libraries".

The book was an admirable summary of Industrial Health in the light of what had been learnt from war time experiences and experiments in the munition factories. It was dedicated to the Minister of Health and carried an introduction by Sir George Newman. It covered the field of Industrial Health in extraordinary detail and deserved a far wider circulation than its high price permitted. Quite apart from its main purpose, the book is still a quarry for ideas, and touches on such themes as the relation between mortality rates in the coal mines and strike ballot results, the charms of alcohol, the differences between industrial and convivial drinking, the effect of women on industrial employment generally, the physiological capacity of women to work, and the dangers of administration based in "a priori" reasoning. As the authors said in their preface, they had at least made plain how great is the influence of employment upon general health.

5.6. Death of Arthur Bacot

On April 13th 1922 a telegram was brought to Greenwood at *Hillcrest*. It announced the death of Arthur Bacot. Bacot had been sent to Egypt at the end of January to investigate

the infectivity of the excreta of lice which had fed on human patients suffering from typhus. Bacot investigated the matter so thoroughly that he became infected himself. On March 24th from Cairo he wrote Greenwood his last letter. He was well and cheerful. On the 26th he developed a headache and became feverish. On April 12th he died. He was buried in the British Cemetery at Old Cairo.

Emotionally Greenwood suffered enormously as a result of Bacot's death. Not only had he lost his best loved friend, the man on whom he had lavished the affection he might have done on a parent but he blamed himself for his death. In many ways, as he said of himself, he was a conscience tormented person, and he could not escape the self inflicted argument that had he not "rescued" Bacot from the tea broker's city office, his friend would still be living. In his delight at having "saved" his friend, he had sent him to his death.

Although Time *must* heal Greenwood seems to have done his best to delay the healing process. For years afterwards whenever Bacot's name happened to come up in conversation, he used to give a pause for reverence. On one occasion, soon after Bacot's death, while walking in Monk Wood near Loughton, he stopped beneath a particular beech tree where the friends had often lingered with their dogs on Sunday mornings, and looking up into the branches murmured softly as though to a ghost, "He died trying to reduce human suffering. What more could he have done?"

5.7. International Health

By 1923 Greenwood was caught up in a mass of investigational work for the Ministry of Health and the Medical Research Council.

Apart from routine work, he sat upon numerous committees enquiring into such matters as quantitative problems of human nutrition, telegraphist's cramp, the physiological action of alcohol, cancer statistics and the propaganda necessary to stimulate the public into seeking early medical advice on this disease. His department was busy on such

matters as mental hospital diets and the measurement of the insane, tests for physical efficiency, secular trends in adult death rates and health conditions in rural areas.

Meanwhile another form of statistical activity was creeping up on him. Long ago he had realised the value of collating international sickness statistics when the means had not been possible. Now international sickness statistics had become a matter of world concern. The ravages of the war had left a mounting toll of disease and brought about circumstances likely to favour a rapid spread of infection. Whatever its causes, the world spread of influenza in 1918 had been a terrible warning. Now there were soldiers returning to their homelands from all over the globe; there were refugees openly or covertly, crossing the old frontiers; systems of government had been overthrown and national boundaries reconstituted. In Russia there was revolution; Poland was stricken with disease; Germany was ruined and starving; the northern villages of France were heaps of rubble; water systems had become polluted; drains had been destroyed, and in large areas of Europe the whole pre-war organisation of sanitation had broken down.

The League of Nations Health Organisation decided to set up a system of standardised international vital statistics so that it would be possible to detect at once where particular diseases were on the increase.

The first step was to prepare a series of handbooks for distribution to the principal European countries describing how these statistics should be compiled. Greenwood's immediate chief at the Ministry of Health was Sir George Buchanan who represented Britain at the League's discussions. He asked Greenwood to assist with the preparation of these booklets. In this work Greenwood was greatly assisted by Percy Granville Edge, the "Major Edge" who subsequently became one of his intimates.

5.8. Major Edge

At first glance Edge and Greenwood had very little in common and in later years people not knowing their background wondered how they came to be so close.

Although Edge says that they first met in 1916 for practical purposes they came together somewhat later. Edge had done very well in the Army. He had joined the Artists' Rifles in 1914 as a ranker. In 1918 he was a Major / Adjutant. In 1919 he left the Army with an OBE and a substantial gratuity which he seems to have spent on a world tour with his eldest son. Then he turned up at Loughton with his family and rented the *Meads*, an ancient house opposite Greenwood's, with a large garden and rambling sheds. Here Greenwood encountered him, jobless and wondering what to do. Chicken farming was then the fashion for former officers and the land next to Greenwood's house was already being used for this purpose unprofitably by another former officer.

Edge was a tall, thin, grey man, sharply spoken, and said to be a martinet at home where his several children were required to observe a strict rota in their use of the bathroom. His interests were horsey and he was a first class rider. But he was not a great reader and from Greenwood's standpoint was no conversationalist. At first appearance one would have regarded him as unpromising material as a friend of Greenwood. Yet, so odd are the ways of chance, that Greenwood caused the Army Major, almost inspite of himself, to become an academic Doctor, and colleagues wondering at the change, found it difficult to call the Major Dr Edge.

They were neighbours. They met on forest walks. Their dogs ran together and barked among the trees. Rosa invited Mrs. Edge to tea and in return both their children played in the Edge's larger garden. The friendship developed. But over-all the Major was short of funds. He was one of a large number of former officers unable to find a congenial civilian job.

Then suddenly an opportunity occurred.

The preparation of the international handbooks required a great deal of organisation. Edge was an adept at organisation. He was taken onto Greenwood's staff at Hampstead.

Edge's functions were to collect information about foreign vital statistics and collate the various official literature supplied by the governments concerned. When there were difficulties he and Greenwood travelled abroad to resolve them. In this way they visited most of the capitals of Europe.

When Greenwood organised foreign trips things tended to go far from smoothly. Railway carriages were crowded or dirty, customs men were officious, hotel rooms were dirty, food was bad and waiters were insolent. With Edge in charge irritations of this sort rarely occurred, or if they did never again in the same place. He complained instantly and to the highest authority. If railway carriages were dirty he had them cleaned; if crowded he requisitioned a whole compartment. Once, arriving late to find the train pulling out, he cried imperiously, "Stop that train", and it was stopped. Hotel managers were subservient, chamber maids kept busy, and if a waiter looked slightingly at a tip, Edge put it back in his pocket. By and by it seems Edge built up the importance of their missions into quasi-ambassadorial proportions, demanded red carpet treatment and usually got it.

Starting from this there grew up between them a kind of mutual protection relationship. Edge protected Greenwood from the rough, workaday officiousness of jacks-in-office, and Greenwood protected Edge from the aimlessness of uncertain employment after his release from the Army life he had loved. As the years went by this relationship mellowed but at the bottom it still remained protective.

5.9. Foreign Affairs

Greenwood got on well with his foreign collaborators. He spoke German well, French with laboured competence, and he had the knack of getting at the meaning of several other foreign languages with the aid of dictionaries. He even tried his hand at Hungarian when he and Rosa offered hospitality to a Hungarian boy during the hungry years for Middle Europe immediately after the war. He might have been successful, but no Hungarian- English dictionary could be procured and he had to make do with a thin little German-Hungarian version which made no mention of grammar. Unlike so many of his

English colleagues he was wholly sympathetic to the statistical work of foreigners, and already knew a good deal about it. He went abroad, he said, prepared to admire rather than to criticise, and as a result he got on well.

Within a few months he became the best known English medical statistician on the continent – in some quarters, the only one. He had dealings with Austria, Germany, Belgium, Holland, Portugal, the Baltic Countries, Italy, Spain, India and even Japan. He already had a contact in United States through Raymond Pearl, once a fellow student under Pearson. The experience and knowledge he gained from all this put him in a unique position, and if anyone in the United Kingdom wanted to know anything about foreign vital statistics, he was almost obliged to pay court to MG, as he was beginning to be called.

He was elected an Honorary Member of the American Statistical Society, and of the Indian National Academy of Science, a Member of the International Statistical Institute and was invited to become co-editor of the Italian statistical journal *Metron*. But what pleased him perhaps more than all this was his election in 1923 to the Dining Club of the Royal Statistical Society founded in 1839 and regarded in London at least, as the most select of all Statistical gatherings. It gave him patience to wait a little longer before attempting to enter the very Ark of Scientific gatherings, the Royal Society itself. As we shall see later on, he had already taken steps, but had withdrawn again.

5.10. The Hampstead Circle

As his name came to be more widely known in fields where medical statistics were of importance, so there began an increasing stream of visitors to his rooms at Hampstead. Here after consultation the visitor would usually be asked to join the "Tea Party". The Tea Parties were derived from Lister Institute days. Everybody in the department met for tea and talk in the middle of the afternoon. In his early days at the Lister Institute Greenwood had been one of the junior members and had tried to force his way with cynicism or barbed wit. Now he was the Chief to whom all eyes were turned for a lead. But he had mellowed a great deal and the lead he gave was a gentle one. He encouraged

general talk, especially among the juniors, and when called on for a lead, usually gave it away from subjects connected with work. He was now, sharply against anyone who attempted to play the cynic as he had once played it. His tea parties became a part of the social life of the National Institute, and friends from other departments used to join in for a chat. One never quite knew who was going to be there, and there was always a chance of meeting some more or less distinguished foreign visitor. Among the regulars were Greenwood's assistants and allies, Hilda Woods, Ethel Thompson, Major Edge, HE Soper, and John Brownlee who was head of the MRC Statistical Department. Even Greenwood's wife for a time joined the team. With the children at boarding school she did a stretch of part time work at the Institute, collaborating with the Misses Thompson and Woods in producing a Study of Heights and Weights of Patients in Mental Hospitals, which was subsequently published in *Biometrika* (1925:xvii:142).

5.11. Epidemiology of Mice

During this period Greenwood added to his various labours by collaborating with WWC Topley, then Professor of Bacteriology at Manchester University, and others in the investigation of epidemics artificially induced into herds of mice. The idea of attacking epidemiological problems by means of direct experiments had been suggested by Topley in 1919 and he had been accumulating experimental data on mouse populations ever since. He would introduce *Pasteurella* infection into a herd of mice and study the infection and death rates according to various conditions. He could, for instance, keep the population static, or he could introduce a given number of fresh mice daily, or at whatever periods seemed interesting. Results could be studied in terms of general mortality, length of time exposed to infection, age of mice and so on.

Greenwood's part in this was to advise on the kind of data required, and to work it up into usable mathematical form. The experiments lasted over a long period and Greenwood's first publication with Topley on the subject appeared in 1925. Whatever other conclusions they reached, the collaborators were again faced with the mysterious factor of periodicity. They found, for example, that the *Pasteurella* infection could remain fatal within a population of mice, over a period of 3 (and one quarter) years, which was longer

than an entire generation of mice, whose lives averaged 18 – 24 months. The rate of mortality came in waves, but there was really nothing definite to explain this wave-like distribution. It was the old problem that Greenwood had discussed with Bacot so many years ago; why, when the general conditions remain the same, should a disease that is always present, suddenly become more devastating?

In 1924 Greenwood was elected to the Royal College of Physicians and the Statistical Society awarded him its Guy Medal in silver. William Augustus Guy had been president of the Society in 1873 – 5.

This was one of the happiest periods of Greenwood's career. The times of struggle were over. Recognition had at last arrived.

At forty four he was the Chief, recognised both at home and abroad as a leading – perhaps *the* leading – authority on medical statistics.

There survives from these days a *Prayer for Daily use in Dr. Greenwood's Department*. A tattered typewritten copy was found in his pocket at the time of his death. The author is believed to have been the brother of one of his statistical collaborators, Miss EM Newbold.

It runs:

> "Our Brunsviga which art in Hampstead,
> Hallowed be the keyer
> Thy addition come, thy subtraction be done,
> On paper, as it is in the Log Tables,
> Give us this day our Daily Results,
> And forgive us our Probable Errors,
>
> As we forgive them that Probably err against us,

Lead us not into Miscalculations,
But deliver us from Karl Pearson;
For thine is the Square Root, the Cos and the Theta,
Work without end, Amen.

(The Brunsviga was a well known calculating machine).

6. The London School of Hygiene and Tropical Medicine (1927 – 1945)

6.1. Foundation

Soon after the establishment of the Ministry of Health, the Minister, Dr Addison, had appointed a committee under the chairmanship of the Earl of Athlon to make a survey of post graduate medical training facilitates in London, with special reference to Public Health, or State Medicine as it was then called. In 1921, by which time Sir Alfred Mond had become Minister, the committee reported on the inadequacies it had found, and suggested that an entirely new Institute of State Medicine should be established, to be associated with the University of London. This suggestion was strongly supported by the Rockefeller Foundation which offered a grant of £450,000 towards such a project. In 1923 agreement was reached between the Rockefeller Foundation, The Ministry of Health and the University. A charter of incorporation was procured in June 1924 and a temporary Board of Management appointed to set the scheme in motion. In its final version the scheme was to create a new School of Public Hygiene which would be amalgamated with the existing School of Tropical Medicine. This had been set up in 1899 at the Albert Docks. Both would come under a single Board of Management and would jointly become a teaching school of the University.

The Board bought a site on Gower Street from the Shakespeare Memorial Association (which had got into financial difficulties) and commissioned P Morley Horder to design, and F Baines to put up, a suitable building.

From the start Greenwood knew exactly what was going on but seems to have been sceptical about the whole project. He had at least two influential friends on the Board of Management, Sir George Newman and Sir Walter Fletcher. He was sounded as to a post in the new school, but showed little interest. When the Chair of Epidemiology and Vital Statistics was created and more or less offered to him, he at first turned it down and thought that Dr THC Stevenson would be more fitted. Yule was shocked by his attitude and urged him to reconsider his position. Greenwood had been grumbling about the amount of statistical "chamber-maid's work" he was obliged to do for the Ministry and Yule pointed out that inspite of his remarks about the "blasted School of Hygiene", as a Professor there he would at any rate have a lot more scope for his own personal interests.

The truth of the matter may have been that although he still had a hankering after an academic post, he was enjoying the "high life" and the international connections of his place in the Ministry of Health. He had just come back from a meeting in Rome, and was due to preside over a League of Nations Health Organisation meeting in Geneva, where he had undertaken to deliver in French a paper on Cancer. Indeed, his foreign connections were proving so successful that there was a prospect of him being offered a permanent post on the League's staff in Geneva. Although he would have hated leaving London, Geneva had many charms, and would have been quite near Rosa's home in Germany. His statistical reputation on the continent was high, and Yule mentions an incident that illustrates one aspect of this. He had a Czech visitor who wanted to see Greenwood. The Czech was on a Rockefeller Fellowship, and on his way over to England called on the Rockefeller secretary in Paris. He mentioned that he would also like to meet Karl Pearson. "Pearson … Pearson …." mused the secretary. "Oh, you needn't worry about arranging to see him. I think he's an assistant in Dr. Greenwood's office …."

The Geneva job came to nothing and early in 1926 Greenwood was formally invited to take the Chair of Epidemiology and Vital Statistics. This time he accepted the position, but with characteristic reservations. There had been a good deal of negotiation behind the scenes and he had succeeded in securing what seemed to him the best of both worlds, Academic and Civil Service. To quote the Chief Medical Officer's Report (1927 p 178)

“Having regard to the intimate relations between the investigatory work of the Divisions of Epidemiology and Medical Intelligence of the Ministry, that of the Medical Research Council and the projected lines of higher teaching and research in the new school, it was felt by the responsible officers of all three departments that a working arrangement between the Ministry, the Medical Research Council and the London School of Hygiene and Tropical Medicine should be made in order to safeguard the co-operation to which I have referred in previous reports. An appropriate scheme has received Treasury sanction, and, for the present, Professor Greenwood will continue to advise the Ministry upon medical questions of a statistical character.”

Greenwood retained his positions both in the Ministry of Health and on the Medical Research Council’s staff, and took in addition the Professorial Chair. His salary was divided among the three employing bodies, he kept his original staff, and would remain at Hampstead until the new School was built.

His position was one of considerable strength. He had his fingers in a great many statistical pies; he had access to official records, power to influence official action, and friends in high places. And above all, having three Masters he effectively had none, and was far from being restricted to the limitations of a purely academic post. He became, in fact, a kind of Dictator in the field of Medical Statistics.

His new appointment was to begin on October 1st 1927, but it was not until July 1929 that he was able to move into his rooms in the new school in Gower Street.

6.2. The Royal Society

In 1926 Greenwood was awarded the Weldon Medal by Oxford University. WFP Weldon (1860 – 1906) had been a pioneer with Pearson of biometry. The following year the Royal Society awarded him the Buchanan Medal in gold. This had been founded by Sir George Buchanan (1831 – 95) in 1894 for distinction in the social sciences. Sir George, a distinguished medical man and principal medical officer of the Local Government Board, was the father of Sir George Buchanan, Greenwood’s senior in the Ministry of Health.

When at the presentation ceremony Greenwood went up to the platform to collect the medal he was puzzled to receive two, a second one in silver. Returning to his place in the body of the hall, Sir Charles Sherrington who was sitting beside him, whispered in explanation, “one for yourself and one for uncle”, thus indicating that the recipients were expected to sell the golden version to a pawn broker.

Now it seemed the distinction Greenwood most coveted was drawing nearer. This was to become a Fellow of the Royal Society, the most exclusive scientific body in the country.

Membership was limited and vacancies only occurred on death. To *apply* for Fellowship was fatal. It was necessary to be *invited* by a canvass of existing Fellows to allow one’s nomination to go forward. Fellowship then depended on election. But they were rare and nominations usually lay for years. In 1921 Greenwood and Yule had both been sounded as possible candidates but there were difficulties. Whereas electoral support could usually be secured for candidates in the older branches of science such as medicine, astronomy and mathematics, it was much more difficult to muster for candidates in the less well established branches of science.

Greenwood and Yule both fell into the latter category. They were certainly not mathematicians as the Royal Society understood that science, and as statisticians their chances were narrow. Greenwood had eventually withdrawn his name lest the nomination of two statisticians at the same time should prejudice Yule’s chance. In 1922 Yule was elected. In February 1927 Leonard Hill, already a Fellow, and Yule got together with a view to pushing Greenwood’s candidature.

In the spring he was nominated and the following May (1928) he was elected to Fellowship. His election gave him enormous pleasure and did much to dispel any lingering feelings he still retained about his background and lack of scientific training. He was now numbered among the top scientists in the country. For the Royal Society and its associations he had a very special affection. It was exclusive, its traditions were historical and stately, and within it science and the humanities seemed to come together. By

embracing him, the Society seemed to confer on him some part of its own distinguished ancestry. It had, moreover, recognised Epidemiology as a new branch of science which he had done more perhaps than anyone else to advance. In the same year the University of London awarded its new professor the degree of Doctor of Science.

Greenwood's election to the Royal Society in 1928 coincided with a somewhat rash offer he made to his colleagues to tip the Derby winner. Although he wasn't in the least interested in horse racing as such, he was mildly intrigued by the pseudo-mathematical processes employed by punters and professional tipsters who based their judgment on Form. Some punters and tipsters without the least knowledge of the mathematics of chance seemed to make quite a lot of money out of their ventures. In a whimsical mood he suggested that a statistician provided with Form books might make a useful tipster. The possibility provided a good deal of merriment and tea-time banter. Someone suggested that he should tip the Derby winner. He took up the challenge and retired to study the Form Books. These being completely useless as far as he was concerned he decided to rely on pure chance. He chose a horse named *Felstead* because it was the name of one of his favourite villages in Essex. Bets were placed and Felstead duly won at 33 – 1. The odds were terrific. How much his colleagues who placed their bets on his forecast, made is not recorded, but the affair caused astonishment and a great deal of hilarity. Thereafter Greenwood was regarded as a useful source of information about potential winners. In October his department organised a dinner to celebrate his election to the Royal Society. The menu, drawn by Major Edge, depicted him as a jockey, surrounded by other wins, *Burning Thoughts* at 5 – 1, *Guards Parade* at 100 – 30 and *Priory Park* at 15 – 2. Whether or not these light-hearted forecasts troubled some of his learned colleague Fellows of the Statistical Society is not recorded, but some years later the Society published a paper demonstrating that on average the forecasts of professional tipsters were slightly *worse* than forecasts picked at random with a pin.

While Hill and Yule had been mustering electoral support for Greenwood at the Royal Society, he had been mustering support for the election of Frederick Marquis, the future

Earl of Woolton, to membership of the Statistical Society's Dining Club. His connection with Marquis came by way of the Culpins.

6.3. The Culpin Circle

Millais Culpin, it will be recalled from the early pages of this story, had been a fellow student of Greenwood's at the London Hospital, had lodged with the Bacots and introduced them to Greenwood. Having qualified in medicine Culpin took several jobs abroad ranging as far afield as Shanghai. On the outbreak of war in 1914 he returned to England and heard the battle of the Marne as he was coming up the English Channel. He joined the Royal Army Medical Corps and settled his family at Loughton. Here Mrs. Culpin, who had hospital experience, organised a Cleaner Milk Campaign when there was an outbreak of diphtheria and several children died. Although a surgeon by training Culpin's experience in Army Hospitals had roused his interest in shell shock and the newly emerging science of psychology. In 1919 he secured an MD from London University with a thesis entitled *The Psychoneuroses of War and Peace*. Thereafter he set up a practice in the West End of London specialising in matters of psychoneurosis.

About this time Major Edge moved away from Loughton and Culpin bought his house, *The Meads*, directly opposite Greenwood's house.

The Culpins were socially minded. They liked holding dinner and bridge parties and bringing people together. They even managed to rouse Greenwood's interest in bridge and for a time he suffered agonies wondering why he was such a bad player though the game seemed to have some affinity with mathematics.

Culpin's new found interest in psychology was going through the same sort of controversies as Pearson's biometry when Greenwood had joined the fray. Conservative members of the medical profession were opposed to its use in medical matters. Although its exponents claimed that it was an emerging science there was almost nothing about it which could be measured and tested. There were no hard and fast facts, no certainties; people reacted differently to different circumstances; it was all trial and error; hit or miss.

Culpin introduced Greenwood to his fellow workers. He was soon won over and gave what statistical assistance he could.

Science or not, the general ideas of psychology were in the nature of a new philosophy, a new way of appraising human behaviour. Hitherto Greenwood had been an unbending critic of actions and beliefs of which he did not approve. He tended to view things as either black or white and to be suspicious of the grey band in between. It was said of him that he liked the statistical approach to problems because it gave exact results and excluded the influence of emotions. But now, under the influence of the new psychology, his certainties began to waver. The grey band between black and white assumed a new importance and he began to realise that as a factor in human affairs emotion was quite as significant as common sense, logic or the wisdom of the leaders.

Once a stern critic of Roman Catholic ritual he now began to have doubts. If church ritual were absurd, what did it matter if it helped the faithful to pray more sincerely? He dipped into the Fathers of the Church for psychological teaching and investigated at some depth the opinions of Thomas Aquinas.

Among the people he met through the Culpins were May Smith and her brother-in-law, Frederick Marquis.

May Smith, a year older than Greenwood, had taken an Arts degree at Manchester and become a teacher in a Training College at Oxford. She had become interested in psychology and studied under Prof McDougall at the Oxford Psychological Laboratory. She was enlisted by the Medical Research Committee during the war as an investigator. She met Culpin early in the 1920s when he was asked to assist an investigation into Telegraphists' cramp on which she and Eric Farmer were then engaged. Culpin introduced her to Greenwood, and they remained close friends for life. When Greenwood took possession of his department in the new School of Hygiene building he secured accommodation there officially for Culpin who was holding a part-time Professorship of

Industrial Psychology, but actually for May Smith who was his collaborator. Thereafter May Smith became one of Greenwood's "family" at the School.

She was a jolly, commonsensical, slightly mannish spinster, widely read and competent to defend any side of an argument. Her recollections of Greenwood are worth recording. He was a mass of contradictions, she said. He would have made a good woman! If he had been anything but what he was, he might have made a good actor. He was extremely kind when approached for help. He had a complex about his height and was predisposed to be hostile to tall, handsome superiors like Sir David Munro (Director of Medical Services, RAF) with whom he had some dealings and who was called by his intimates the Arkangel. His rationalism was constantly at war with his emotions. He had a lack of spontaneous human warmth that frightened practically everybody.

This lack of warmth coupled with extraordinary kindliness was remarked by many people.

On one occasion at the Culpin's house when May Smith was present, Mrs. Culpin's small daughter Jo greeted Greenwood by throwing her arms round his neck and giving him the sort of kiss she usually reserved for her own father. He was utterly taken aback and covered with confusion. The Culpins, primed in psychology, determined to remedy this. The next time Greenwood and his wife called for coffee, Mrs. Culpin and May Smith kissed them both and in the ensuing confusion Greenwood's embarrassment evaporated. Because he was shy of Christian names, especially of his own, they re-christened him Jim, and Jim he remained to the Culpins and their friends.

This shyness over Christian names is very apparent in Greenwood's private writings. In his diaries, apart from members of his own family, barely a Christian name occurs. May Smith and Tony Hill have their surnames dropped. Edge, Culpin, Isserlis, Yule, intimates of many years, remained Edge, Culpin, Isserlis and Yule to the very end. Yet, curiously, when he had collaborated with Collis over the book on Industrial Health, Collis had called him Jonathan and signed himself David.

The Culpins had a great belief in the humanising effect of nick-names. In 1927 at their house Greenwood met the notorious Dr George Groddeck and his wife. Groddeck, the "Wild Analyst", was a German pioneer of psycho-analysis, an intimate of Freud himself and the centre of numerous controversies. Greenwood was astonished to find that the great man and his wife had been reduced to human proportions by the Culpins calling them Groddy and Froddy.

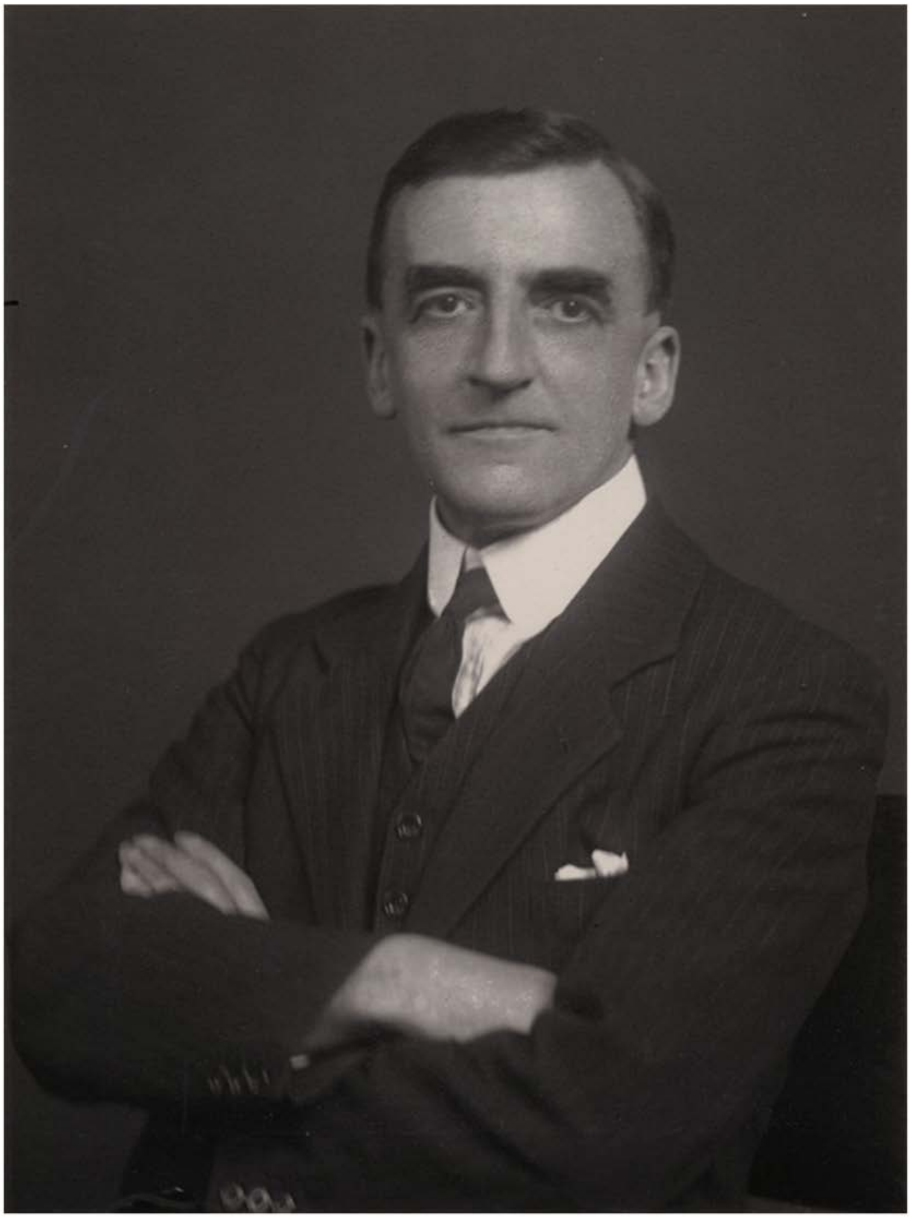

Figure 3: NPG x167957, Major Greenwood by Walter Stoneman,bromide print, 1931 (© National Portrait Gallery, London).

6.4. The Academic Plateau

In July 1929 the School of Hygiene and Tropical Medicine was formerly opened by the Prince of Wales. Greenwood and his staff took possession of a suite of rooms at the corner of the first floor overlooking Gower Street, and here, as the "Chief", he remained until the end of his active life. At the centre of the suite was a Common Room, lined with reference books. Here the "Tea Parties" were held as at Hampstead, lineal descendent of the social get-togethers at the Lister Institute.

Greenwood had now reached a kind of plateau in his progress. Hitherto he had been climbing in one way or another, by intent or accident. Now he was forty nine and had reached a point where he could remain comfortably for the rest of his working life, or progress further by attaching himself to a party, or a cause, or a person. He had ample opportunity for doing the latter, and in later years we find him writing speeches and preparing information on a personal basis for dignitaries as highly placed as Cabinet Ministers, but he seems to have preferred to stay where he was and remain as he hoped an influential "back room boy".

Having perhaps unconsciously made this decision his activities tended to become static and repetitious. His best work was done. Darwin had laboured for Evolution, Galton for Eugenics, Pearson for Biometry, and dare he aspire to the same distinguished company? Greenwood for Epidemiology. These studies had all now been recognised as sciences. Now it was time to encourage younger men to aspire to new ideas,

He fell into the University routine, preparing lectures, teaching, sitting on Boards and Committees and generally acting as adviser on all the subjects with which he was connected. He continued his work for the Statistical Committees of the Ministry of Health and the Medical Research Council, and dug in deeper with the affairs of the Royal Statistical Society and the College of Physicians. Life increasingly became a routine and there are hints in his writings that he was wasting too much of his time vetting other people's work and was losing his old zest for matters purely statistical. There was a tendency to a reversion to his youthful aspiration to study history.

At home, too, life had become something of a routine.

7. **"Hillcrest"**

7.1. His Home

Hillcrest was his home from 1913 until his death thirty six years later. It stood on top of Church Hill, Loughton, Essex, near Epping Forest with a fine view southwards towards London. It was one of three fairly large houses on three floors built about 1900 and called by contemporary estate agents "for the professional classes". It had three reception rooms, a butler's pantry, a large kitchen, a scullery, a coal shed and a servant's WC. downstairs; four bedrooms and the usual offices above this, and under the roof two Maid's bedrooms and three attics. At first Greenwood leased the house from the widow of the former occupant who had been a local doctor. Next door was a retired bank manager and beyond that the Rectory. Compared with a narrow terrace house in the Hackney Road where he had lived as a boy, *Hillcrest* was a veritable country mansion. In the early days, cycling home in the moonlight from the station, what a joy it was to walk slowly up Church Hill and see the moon reflected in the windows of his house.

Considering the smallness of his income when he moved into *Hillcrest* it says much for the low cost of living that he was able to maintain a housemaid, a cook and a part time gardener.

In the dining room was an unidentified oil portrait of a lady in a beehive hat holding a bunch of roses and dating possibly, from the early 19th century. It was thought to be one of his grandmother's relatives. He scorned to have it properly cleaned on the jesting grounds that it might then prove to be worthless. Close by was a hideously unflattering photograph of his mother, and over the side-board prints of Virchow and Helmholtz whose works he had once studied. The drawing room walls were embellished with oil paintings in heavy gilt frames done by Leonard Hill and a large framed engraving telling the story of a bell, a German legend, which came from Rosa's family. Up the staircase

hung a series of twelve engravings depicting the months of the year. In the bedrooms were more framed cathedrals, an engraving of Dr Samuel Parr, a gift from Yule, and coloured lithographs of Wellington's first encounter with the French (at a Military School in Brussels) and Nelson's departure from his mother, gifts at some time to his sons. There was a fine water colour copy of an old engraving of Shoreditch Church, possibly done by Greenwood's mother, and a few small water colours by Sydney Burchell, a relative of his grandmother.

His furniture was an inherited mixture, Victorian upholstery in the drawing room from his father, a Bechstein grand piano from his mother, his father's surgery desk, and odds and ends picked up at sales, notably that of the Hills when they left Loughton. There was a magnificent wardrobe in his bedroom, thought to have originated from his German parents in law, and a fine side-board and set of six chairs in Heppelwhite style, made by a local joiner, Chiswell, to an original Heppelwhite card table design, the gift of his father. The idea for this seems to have originated from WW Jacobs, the comic novelist who was an acquaintance and local resident at Loughton. He gave Greenwood an early 19th century tea caddy which was used as a cigarette box. The Heppelwhite chairs were somewhat shamed by a cheap dining table which was constantly covered by a thick, plushy tablecloth.

Greenwood and Rosa were never collectors of fine things, at first without the necessary means, and later, when they had them, old habits had died hard and they were not really interested. One of Greenwood's few concessions to personal luxury was the installation of an electric fire on the ceiling over the bath. As they grew older the furniture of their younger days began to wear out but they had no inclination to replace it. Their blackout curtains from the First World War survived to do similar service in the Second.

7.2. The Study

The most important room in the house as far as Greenwood was concerned was the Study. It was a small room, lined with overlarge bookcases. The wall space above them was decorated with framed pictures of English cathedrals, a photograph of Arthur Bacot

and another of his army colleagues in 1917. The desk under the window was a cheap knee-hole affair that had once been the property of a mid Victorian doctor in Bethnal Green and had been part of his Father's surgery furniture. The desk light was thoroughly inefficient and the telephone was an old fashioned bracket affair, fixed at such a height as to be uncomfortable and inconvenient. Indifferent to inefficiencies of this kind Greenwood never bothered to have them put right.

He spent a great deal of his time in the Study, poring over his papers, twirling the handle of his Brunsviga calculating machine, and typing endless letters and draft reports.

In the household the Study acquired the aura of a Headmaster's study in a school. It was a place for serious conversations. Greenwood in the dog's armchair was Greenwood in slippers, but Greenwood in the Study was the Headmaster at his most awesome. When he sent for his children there they went with flutterings of anxiety. When he held consultations with his wife there, great events were thought to be afoot. When the maid brought him tea there, her knock was especially subdued.

7.3. His Books

His collection of books was large and gradually expanded into the bedrooms and the attics. Yet it was an extraordinary raggle-taggle affair, reflecting his wide range of reading. He was no collector in the general sense of the word. His library was of the magpie variety, odds and ends picked up in a variety of casual and accidental ways. Able to get whatever he wanted from the many libraries to which he had access, he never bought expensive works, but preferred to pick up stray titles that caught his eye on bookstalls and in second hand lists. Mathematical and statistical works predominated, but they were heavily counterbalanced by a confusing mixture of Latin classics, biographies of bishops and headmasters, detective novels, school text books, historical works, poetry, Dickens, Scott, Gibbon, Lecky, Macauley, Census Reports, Medical Monographs and hundreds of unbound pamphlets. They were an index to his interests and many of them had personal associations. Thus, in a copy of Austin Dobson's *XVIIIth Century Studies* he

wrote "Purchased for 2/- on September 28th 1945, when coming to the School of Hygiene for the last time as Professor on the active list. MG.".

Towards the end of his life, after Rosa's death, when he became lonely and his interests began to flag, he turned towards his books and started to catalogue them as though listing old friends that were still with him. After 31 closely written pages he gave up. The end of his collection came as an anti-climax. Heaven knows how many books there were, several thousand at least, but of the three book dealers invited to tender for them, two, without seeing them, offered £20 for the right to pick what they wanted. The third, rather shrewder, sent a cheque for £250 and on the following day collected the lot. When asked many years later what he had done with them, he said he had made his profit on the sale of the runs of *Biometrics* and the Journals of Royal and the Statistical Societies. The rest, about 90% he still had in their original bundles gathering dust in his store-room.

7.4. Intruder

One night in the early 1920s, when everyone was a-bed, somebody blundered into the garden at *Hillcrest* like an incompetent thief, perhaps after Rosa's chickens. Shrill cries from the bedroom window drove the intruder away and next morning Greenwood bought a burglar alarm from the Civil Service Stores in the Strand. It consisted of a metal stake fitted with a runner which contained a large blank cartridge. The stake was driven into the ground, the runner raised to the top position and held there by a trigger. Attached to the trigger was a long, thin piece of cord which was stretched across the area requiring protection. On the trigger being pulled by the cord, the cartridge container dropped onto a pin and exploded with a terrific detonation.

The first time the thing was set up, in front of the house across the full width of the garden, the only intruder it caught – with devastating effect – was the postman. Learning from experience, the gadget was set up behind the house where its only effect was to terrify Rosa when she went to feed the chickens and forgot about the trip cord. The alarm was soon abandoned.

The chicken run at *Hillcrest* was an important feature. The chickens had been installed during the First World War and their descendants survived into the second when some of them had a harrowing experience. The broody hens had been banished to a somewhat dilapidated wooden shed where, during an air raid, an incendiary bomb fell through the roof and singed their tails. According to Greenwood they were so shocked by the experience that they began laying the very next day.

7.5. The Daily Round

On weekdays Greenwood was seldom at his best at breakfast, and the children at any rate felt the tension relax when he went out into the hall, laced up his boots sitting at the bottom step of the stairs, and set off for Town. In the old days he used to cycle to Loughton station and go up to Liverpool Street by train. It was one of the last suburban lines to defy modernisation. In the middle 1920s he grew tired of it and bought a car, a Morris Oxford, which he called *Dinah* (Yule bought a car about the same time and called it *Susie*. The two cars used occasionally to pass messages to each other through their owner's correspondence). Thereafter he went up to town by road, using a complicated route to avoid all the main thoroughfares. At Woodford he used to pick up Major Edge, and when Edge bought a car of his own, they used each other's cars in turn. The car established a weekly ritual. Every Saturday morning Greenwood and Edge used to clean it, with the dog barking around them. Greenwood wasn't a bad driver, and was never involved in any serious accident, but he was repeatedly scratching the wings on gate posts which vexed him exceedingly. When anybody else knocked into him his fury knew no bounds. On one occasion when his elder son was driving the car, they were stopped by the police for speeding. When the case came up before the magistrate Greenwood, as a witness, scandalised the court by refusing to say how old he was on the grounds that the question was irrelevant and impertinent.

Although the car was a far more convenient way of travelling up to town it provided endless scope for squabbles and personal sulkiness. Apart from scratches and bumps and mechanical failures and indignantly disputed repair charges, there was the reluctance of his sons to help with the cleaning, or the lateness of Edge, or the badness of the roads, or the lunatic driving of others. Neither Greenwood nor Edge were at their best in the early morning and a cross word from one would elicit a cross word from the other. There were periods when, as a result of some imagined slight, they travelled alone in their own cars. But the squabbles always blew over and the car went up and down year after year covering thousands of miles of the meaner back streets of the north London suburbs.

At the School Greenwood's life was on a lordly plane. He looked in on his colleagues, ran through his mail, and fulfilled the day's engagements like an elder statesman. He lectured, he gave advice, he sat on numerous Committees, he presided over the daily tea parties, and enjoyed himself immensely at intimate little gatherings of the Learned Societies of which he was a member. By now he numbered among his friends many men of distinction and it was pleasant to have snug little lunches with the great ones at the Atheneum, Claridges or in gracious mansions in Wimpole Street.

He came home at about six o'clock, was greeted wildly by the dog, and ceremonially unlaced his boots on the bottom step of the stairs. He had a glass of sherry and a roast supper and then, after general chatter of the day, retired to his study "to work". The nature of "the work" varied, but the name was generic and covered any intellectual process.

7.6. Dogs and the Sunday Ritual

If in matters of human love Greenwood was horribly shy, he loved dogs unashamedly. With them his emotions were quite unguarded. He kissed and caressed them as he would never have dared do with his children. He elevated doggy companionship to absurd height, and drew from them untenable human parallels. Many years later in a BBC broadcast he defended Euthanasia. He referred with a breaking voice to the pitiful scene of an old dog dying slowly of a painful disease. If it were right, he argued, to put the dog

out of its misery, and he maintained that it was right, then surely the same mercy ought to be extended to human beings.

He only kept one dog at a time, usually a fox-terrier. The first was *Ami*, the companion of forest walks with Arthur Bacot's *Jake*. *Ami* grew old and ill and too testy to be teased by the children. One night in 1921 Greenwood and Culpin "put him to sleep" with chloroform. The business went off badly and Greenwood was shaken for days. Then he did something that was completely out of character. Never a Do-it-yourself man, and ham-fisted at handy-crafts, he got a piece of wood, part of an old dart board, converted it into a marker for the dog's grave, and laboriously carved the name *Ami* into it. Yule's obituary comments on the occasion are worth quoting; "I hope he is in some pleasant paradise where rheumatics and diarrhoea have fled away and kindly angels will let him sleep on their laps until in a new found youth he will take to chasing the deer in the celestial groves, and the deer will chase him, and angels and dogs and deer will all thoroughly enjoy the game, and a panting little dog will lap up a bowl of ambrosia to refresh him after the run."

The next dog was *Bayes* called after the Reverend mathematician of that name, whose works Greenwood was then studying. *Bayes* was so timid that on one occasion he ran yelping away from the sound of his own wind. He was found dead in the road, killed by a motor, and was buried by the side of the brook that runs through Loughton.

Then came *Derby* who arrived on Derby Day in 1929, a short-tempered brute that flew into rages whenever the doorbell or telephone rang. In January 1935 Greenwood confided to his diary, "*Derby* bit Jo (Culpin) savagely. Lost my temper and beat him savagely. Feel very ill. My breath. Still feel ill. A first memento mori …." This was the way dogs affected him. *Derby* was "put to sleep" in August 1939 and Greenwood wept over his grave …. The last dog was a fox terrier from Harrods called *Toby* who was put away in 1948.

Dogs were an important part of the Sunday ritual. This had begun before the 1914 war, when Greenwood first moved to Loughton in the wake of Leonard Hill, and became hallowed by sentimental associations. Leonard Hill was the originator, when Greenwood was still an unwilling medical student. Hill used to bathe in the Wake Hill Valley Pond in Epping Forest every day of the year before breakfast. On one winter occasion when he came to break away the ice at the end of the diving board he found a drowned man. When Hill left the district Greenwood became the High Priest of the Sunday morning bathes.

Long before breakfast on summer Sunday mornings the acolytes would converge on Greenwood's house, dogs barking and cycle bells ringing. In the old days there had been Hill and Bacot and one or two others, but later came Culpin and after him a slightly reluctant Edge. Greetings were shouted from bedroom windows; a hasty cup of tea downed, and then, in old clothes, with scarves round their necks, the party would pedal up to the Wake Arms through the forest and then run smoothly down the new road to the Wake Hey Pond. Here a diving board had been set up by Hill and the water weed cleared away from the central part of the pond. The practice was to dive in, swim round in a circle and if possible get possession of a yellow water lily. The dogs were expected to participate, and if they hesitated they were pushed in from the diving board with shouts of merriment. Afterwards cigarettes were lit under the birch trees while the bathers dried themselves and tried to clean the mud from between their toes.

After breakfast in their own homes, some of the bathers would converge on Greenwood's house for a morning walk in the forest. This began just as the church bells stopped ringing. Monk Wood was the focal point of the walk, the most solemn and splendid part of the forest. Here the great beech trees stood up tall and straight, and there was a hush as though the breezes were unable to penetrate into these cathedral-like depths. Here on a fallen tree the walkers would light their cigarettes and shout to their dogs to stop chasing the deer, real or imaginary.

For Monk Wood Greenwood had a special reverence. Here at least he could unleash his carefully guarded emotions and admit that he was in the presence of beauty. As he grew

older and his friends of the past dropped away, so the beauty of Monk Wood was enhanced by association and he could almost weep before "the oak to which I make my vows". "I should wish to die quite suddenly in Monk Wood" he wrote in September 1935. "Took off my cap in homage" to Monk Wood (10th May 1936).

The forest in those days was almost deserted and the walkers knew practically everyone they met, if not by name, then at least by their dogs.

These forest walks were among the happiest moments of Greenwood's life. His friends might drop away but always his dog remained, that grew restive after breakfast on Sunday mornings, and barked loudly when the boots were produced, and who never grew tired of the old familiar round. His dog was always eager, always anxious and frisking, never tired. When at last Greenwood found himself walking alone the beauty of the woods brought tears to his eyes and he believed the end was near.

When he died there was found in his pocket book a faded typescript copy of Rudyard Kipling's poem, *The Power of a Dog*:

> There is sorrow enough in the natural way
> From men and women to fill our day;
> But when we are certain of sorrow in store,
> Why do we always arrange for more?
> Brothers and sisters I bid you beware
> Of giving your heart to a dog to tear.
>
> Buy a pup and your money will buy
> Love unflinching that cannot lie-
> Perfect passion and worship fed
> By a kick in the ribs or a pat on the head.
> Nevertheless it is hardly fair
> To risk your heart for a dog to tear.
>
> When the fourteen years that nature permits
> Are closing in asthma or tumors or fits
> And the vet's unspoken prescription runs
> To lethal chambers, or loaded guns.

Then you will find--its your own affair
But--you've given your heart to a dog to tear.

When the body that lived at your single will
When the whimper of welcome is stilled (how still!)
When the spirit that answered your every mood
Is gone--wherever it goes--for good,
You still discover how much you care
And will give your heart to a dog to tear.

We've sorrow enough in the natural way
When it comes to burying Christian clay.
Our loves are not given, but only lent,
At compound interest of cent per cent.
Though it is not always the case, I believe,
That the longer we've kept 'em the more do we grieve;
For when debts are payable, right or wrong,
A short time loan is as bad as a long--
So why in Heaven (before we are there)
Should we give our hearts to a dog to tear?

After the Sunday morning walk came a roast lunch preceded by sherry. If the Greenwoods were thrifty over their furnishings, they ate very well. It was a partiality, Greenwood said, inherited from his father. Greenwood senior, a great one for long cycle tours, used to stay at remote inns and insist, no matter how late the hour, on a roast chicken supper.

Apart from a partiality for German sausage, which he used to bring back from Schmidt’s, his tastes in food were not exacting, though as he grew older he rather fancied himself as a judge of wine. Reading his diaries of the middle thirties, it is extraordinary to notice how much old port seems to have survived. He had a hoard of 1908 port which he drank on special occasions and which lasted down to the 1940s. He kept a stock of wines, but was a very restrained drinker, and seldom touched spirits.

Rosa had two cats, a tabby and white one who developed a taste for sharing Sunday lunch if they had the opportunity. They used to settle themselves on each arm of her chair and wait patiently for anything that was offered.

After the Sunday lunch, when the children were younger, he read to them for about an hour – he was a first class reader and never seemed to tire and then withdrew to the Drawing Room for a nap on the sofa. Sleep was induced by means of a dull book.

Usually he retired to bed at about 11.30, read for a little by the light of a candle and nibbled plain chocolate which he kept in a drawer beside the bed. From time to time bits of broken chocolate would find their way into the bed and Rosa was most concerned that the housemaid should not misunderstand the brown stains they produced. On one occasion in the midst of a nightmare he seized the chocolate and scribbled with it on the wall. On another he cried out loudly, "God has a built a temple up to heaven …" His dog slept at the end of the bed or on a triangular sofa at one corner of the room.

7.7. Christmas

Ever since the children had been old enough to appreciate it, the Greenwood's Christmas had tended to follow the German pattern. Christmas Eve was the moment of high magic when the presents were given out. Stockings were only nominally used. The German method guaranteed more sleep for everyone concerned.

Christmas officially began with tea on Christmas Eve when the Cake first made its appearance. This was usually preceded by a walk in the forest if the weather were suitable to keep the children out of the house while things were being got ready. During these walks Greenwood would hint darkly that *if* there were any fairies about, it was only on Christmas Eve that they could be seen by mortals, and the children, openly expressing doubt, nevertheless kept a sharp lookout for them. The forest at that time of the year, and time of day, was usually grey and gloomy and well suited for the appearance of the supernatural, but the trouble, as everyone suspected, was the dog. At the sound of his barking and scampering feet, the fairies all fled away ….

By the time they got back, Uncle Ernest had arrived from Brighton in his car. Uncle Ernest was Greenwood's uncle on his mother's side, a large Victorian looking General

Practitioner, who combined an old fashioned manner with a surprising fondness for newfangled gadgets, and who could always be relied on for the most exciting Christmas presents.

Nobody was really hungry for tea and the Cake was scarcely nibbled. Then came a hush of expectation while Rosa went into the Drawing Room where the gifts were laid out on the sofa, and lit the candles on the tree. At the signal of a brass hand-bell, everybody went into the Drawing Room, presents were claimed, chocolates and fondants from the Civil Service Stores were opened and handed round, and the dog was bundled out of the room while the crackers were being pulled. The grown-ups sat in the big Victorian armchairs and dipped into the Christmas books.

About half past seven Pendred, the family doctor arrived, and soon everyone went in to supper of York Ham from the bone, Trifle and Stilton Cheese. Afterwards Rosa withdrew to make coffee in a deliberately leisurely way, while the men stayed behind with the Port. At this point the conversation between the three doctors usually became fascinatingly gruesome and the children lingered on to listen. Pendred would recount a curious case of gangrene he had just seen; Uncle Ernest would cap it with an extraordinary ulcer he was treating, while Greenwood, who had long since ceased having anything to do with practical medicine, would recall some parallel incident from his almost historic days at the London Hospital. So it went on until some point of discussion arose, like the way increasing age seemed in some people at least, to diminish the fear of death, and the children crept away to bed ….

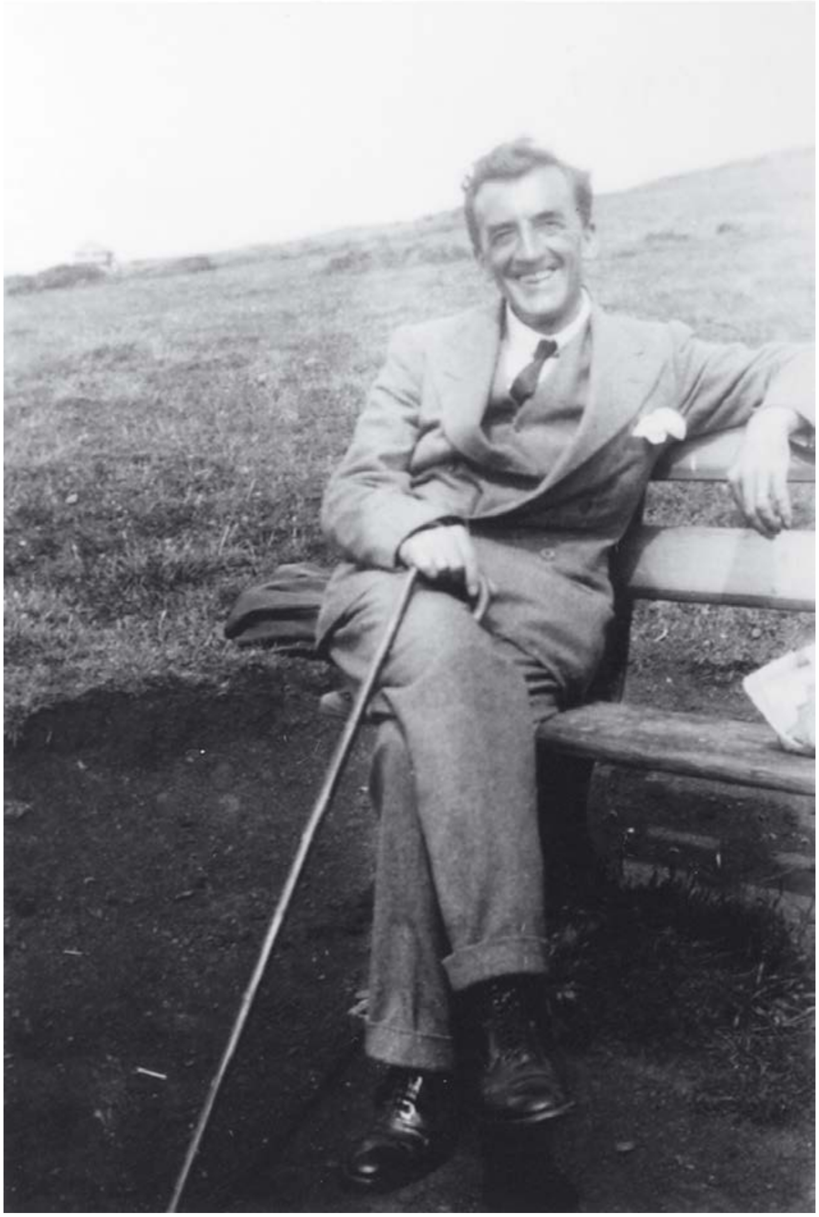

Figure 4: Major Greenwood, relaxed and smiling (reproduced from Farewell, Johnson and Gear, *Journal of the Royal Statistical Society, series* A (2012); 175 (3): 799 - 811).

8. Major Greenwood, The Man

8.1. Music

When Greenwood inherited his mother's Bechstein grand piano he tried for a time to pick out tunes from an old song book, and even sang *Down Among the Dead Men* to his own

one finger accompaniment. Pearson was astonished when he heard about this and commented, “All bad musicians ought to be crucified because of the torture they inflict. Ask Mrs. Greenwood”.

His musical interlude was mixed up with the mathematics of harmony, a Prout’s *Harmony* coming into his possession about this time.

He loved Gilbert & Sullivan Operas which he knew well, and from which he could recite long passages. He had a partiality, if he had to listen to singing, for the kind of deep base Russian songs favoured by Chaliapin. But he had no real feeling for music, possibly on account of his emotional shyness. He was never one to rave openly about anything except dogs, and to listen to music with a tight rein on the emotions must have been like wearing ear plugs.

His favourite musical anecdote related to Samuel Johnson, rolling about in boredom during a harpsichord recital. His hostess said to him in mitigation, “It is a very difficult piece,” to which he replied, “I would to God, madam, it were impossible.”

8.2. Appearance

Too many photographs survive to make any description of Greenwood’s appearance worthwhile, except that he was a little over 5 foot 4 inches, slim, small boned and with delicate hands. At times he was sensitive about his height and was hurt to discover that Culpin’s small daughter called him “The Little Professor”, her father being large and bulky. Curiously many of his most intimate friends were tall. In the early days little Greenwood used to follow big Leonard Hill into the dining room of the London Hospital. At the end it was tall Major Edge who followed him into the dining room of the School of Hygiene.

He had little real interest in clothes though the gay antiquity of his Doctor’s robes amused him. He was a traditionalist. Having found something that suited him he went on wearing it regardless of any change in fashion. In 1897 he started wearing brown “Trilby” hats

and continued the style and colour for the rest of his life whenever he felt that a hat was necessary, Usually he went bareheaded but shared Arthur Bacot's view that bank managers and the like regarded hatless men with suspicion. Latterly he took to wearing a beret.

For forest walks he wore knee breeches and stockings, and sometimes even leggings.

In the late 1920s he turned up at his sons' Public School at Chigwell wheeling a bicycle and wearing a Norfolk jacket, knee breeches and a little old fashioned cycling cap. He caused a minor sensation and was unfavourably compared with "Old Daddy Dawkins" the village eccentric.

8.3. Conversations

As a young man he had attended classes in elocution and never forgot them. He knew all the techniques and practised them. Once, while staying at a Guest House on holiday, he gave a recitation during a Saturday evening concert. While delivering a melodramatic monologue late comers paused outside the concert room.

"What's happening?" somebody asked, listening at the door.

One of the party peeped inside, "Some chap trying to burst a blood vessel," he reported on Greenwood's energetic performance.

Although he prepared his lectures extremely carefully, he spoke better *extempore*. With prepared notes his style was perhaps too precise and his delivery lacked the warmth and colloquialisms so necessary to put an audience at its ease.

As a conversationalist he was first rate and a good mimic when telling stories, but he had to be in the right mood and with the right company. His phenomenal memory and wide reading gave him ample to contribute on almost any subject that was slightly out of the ordinary. Indeed, he rather specialised in what somebody called "left-handed"

information. In criticism, even of his friends, he could be icily out-spoken, and sometimes coined phrases that were too clever to be forgotten easily. As a young man he was cynical and censorious, and his wit had a cutting edge that hurt, but as success came to him he mellowed and became genial. To many he appeared unapproachable, cold and too critical, but when approached he was kindness itself. Without ever having justified it, his family, particularly his children, when young, were a little afraid of him. The basis of this awe was possibly his apparent inability to come to terms with "silliness", a word of criticism that he used frequently.

8.4. Reading and Working

He read rapidly and with a great deal of skipping, and over the years he developed the knack of getting what information he wanted in a very short space of time. An analysis of his diaries for 1934 to 1935 shows the following books read:

	1934	1935
Scientific	17	23
General	46	33
Latin Classics	7	5
"Shockers"	44	20
	114	81

These were in addition to the scientific papers with which he dealt as his stock-in-trade. After 1935 he grew tired of "shockers" and the number for Latin classics increased, Virgil, Galen, St. Augustine, Ovid, Tacitus, Roman Law, Cicero, the list rolls solemnly on, lightened occasionally by Catullus, Appulcius, and the Decameron in English. Reading HG Wells' "Autobiography" he found he had much in common with the author, vain, excitable and full of inferiority complexes. Reading about a rich man he commented, "There is partly a touch of East End envy about reading about very rich people."

His subjects ranged over a wide field; Medicine, biometry, evolutionary processes, statistics, mathematics, Epidemics and the history of diseases, psychology, Industrial health, biography, particularly of statesmen and bishops, chess, architecture, some aspects of natural history, the Latin classics, philosophy, Aristotle, Aquinas, Plato and Hegel, religion (he knew a good deal about liturgy), history, law (stimulated by his father's interest in it and his son's profession), politics of Fabian – Bacot variety, poetry from Shakespeare to Bell's Classics ….

He said of himself that he was not an original thinker (though he thought his father was) and that he worked extremely slowly. He used statistical processes to force his thoughts along and only felt safe to proceed along any particular line of enquiry when he had an incontrovertible series of observations to support him. This, an unkind critic might observe, was the use of statistics to overcome an inferiority complex, and yet there could be some truth in such an observation. In controversy Pearson always relied on facts and never appealed to the emotions. Emotion, Pearson had decreed, had no place in Science. This was part of Pearson's intellectual prison-house. Almost to the end, but not quite, Greenwood followed the Master, unwilling perhaps to recognise that no matter how important Truth may be, Emotion usually carries the casting vote in the short run.

He composed most of his writings on a typewriter, having first begun to use a machine in 1897. His style developed in the writing of official reports. It was extremely clear and concise. When in his later years he wrote on subjects that became more literary, he adopted the leisurely style of his Victorian favourites, with an over-fondness for parentheses and words italicised or underlined. He became so adept at typing that the sheer speed of writing enabled him to send long letters to his friends, which, if recovered, would in themselves form a kind of autobiography. Yule was a typist too, and Yule's letters to him, which have survived, serve as index to the writer's life. On one occasion, when Greenwood was involved with statistics relative to Liquor Control, he tested the effect of alcohol on himself in the following way. He typed from a copy a Latin verse. Then he drank a glass of port. After a suitable time, he typed the next verse. Then he took another glass of port, and typed the third verse …. And so on. At the end, he counted the

number of mistakes in each verse and related them to the number of glasses of port consumed. Not perhaps wholly scientific, but at least a practical form of experimentation.

Critics have commented that Greenwood's literary style was extremely good and deserved a wider readership than it obtained in his lifetime. It did, but its literary placing was too narrow. His worked appeared almost exclusively in scientific publications, whereas much of it would have been appreciated by a non-scientific public in journals of wider appeal.

8.5. Experts and Amateurs

He had a hearty and long continued dislike of "experts" and "Leading authorities", especially when they made pompous *ex cathedra* pronouncements. He may have caught the germ from his father who was constantly deploring that legal coroners should be permitted to make medical decisions. Certainly he hated his father's "expert" advice that he should become a doctor, and when he did become a doctor, he soon joined Pearson in attacking the medical "experts". In the very last year of his life he commented in a letter to his younger son, "I should like to head a crusade for the extermination of "experts" and "leading authorities". I have never yet met a really first rate researcher who *liked* to be called an "authority".

Emotionally his attitude was probably grounded in a mixture of juvenile rebellion and the feelings of inferiority he felt when he first mingled with the scientific researchers at the Lister Institute. He rationalised it into something larger and gave it its most formal expression in his Linacre Lecture at the University of Cambridge (1943) when he spoke on *Authority in Medicine, Old and New*.

If he disliked people who claimed to be "experts" he had a partiality for amateurs, little men of no particular training, with a passion for investigating curious problems in obscure back rooms. During a highly technical discussion at the Royal Statistical Society he commented, "I belong to that diminishing class who are not experts or authorities on

anything whatever, but are merely inquisitive people" (*Journal of the Royal Statistical Society* 1949; 112(1); p 25). Arthur Bacot was his classic example, but he soon found plenty of others. He believed, with a good deal of evidence, that the enthusiastic amateur, because of his lack of formal training, was likely to produce more original work than the trained professional. One of his heroes was William Farr (1807 - 1883) who, with an absurdly inadequate training, had founded the science of Vital Statistics. Another was John Graunt (1620 – 1674) a draper, who was the first to make a statistical study of the Bills of Mortality.

He had a profound belief in dedicated curiosity and recalled with regret how scientific methods were ousting curiosity from the affairs of the Statistical Society. Once, in the old days, a preponderance of the Fellows had been non-mathematical gentlemen in all walks of life who shared a common curiosity. They counted things, made unscientific observations and frequently drew wrong conclusions from them. In doing all this they opened up a wide range of interesting enquiry. Later the Fellowship had largely changed. The laymen were vanishing before a tide of professional "experts", who counted nothing, and relying upon officially published statistical evidence, wrangled for hours over mathematical procedures. In their latter years Greenwood and Yule used to shake their heads over the science they had been instrumental in developing, and admitted that the new mathematical processes were quite beyond them.

There is some evidence that when Greenwood collected together staff for his various departments he deliberately avoided "experts", and preferred willing amateurs. Certainly few of his intimate collaborators and assistants had any formal mathematical training.

9. Notable Events (1931 – 1939)

9.1. Visit to the United States

In the autumn of 1931 he accepted an invitation from the Johns Hopkins University in Baltimore to deliver the 20th Herter Lectures in the first week of December at the School of Hygiene there, the elder sister of the London School of Hygiene. The invitation came

at an opportune time. He was mentally exhausted and suffering from bouts of depression. He needed a rest, and decided to take the slowest possible boat and enjoy the sea voyage.

Not over enthusiastic about the United States before he set out, his views were rapidly changed when he got there. The view of New York, coming up over the horizon he thought the grandest sight he had ever seen. He was greeted on the quay as a near celebrity and experienced the enormous hospitality of the Americans.

He stayed in Baltimore with a friend of long standing, but only seen at rare intervals. This was Raymond Pearl (1879 – 1941), First Professor of Biometry and Vital Statistics at the School, whose hand can almost certainly be traced in the occasion for Greenwood's visit. Pearl, of New Hampshire stock, was basically a biologist and had worked at many universities. He and Greenwood had studied biometry together under Karl Pearson in his session of 1905-6. Pearl returned to America and they had not met again until 1917 when Pearl came to England on war service. Thereafter, whenever Pearl came to Europe he and Greenwood met, and as Greenwood said in his obituary on Pearl they had spent many pleasant nights together in ancient inns in England and Germany, and sipped port together at the Royal Statistical Society Dining Club. Pearl was an immensely enthusiastic almost boyish, likeable man, whose particular life study was genetics and the process of living and ageing.

Greenwood chose as his subject for the three Herter lectures, *Epidemiology: Historical and Experimental.* They were in his best and most concise literary-scientific style, and while they certainly pleased his audience, must have set some of the members wondering at the extraordinary breadth of scholarship English academics brought to bear on their subject. To the practical American mind, Epidemiology was largely a matter of facts and figures and techniques confined within a fairly narrow scientific boundary, but somehow the lecturer from London had broken down the boundaries and merged science with the humanities at large. Instead of ignoring the Ancients, he spoke of Galen and Hippocrates with respect and indicated that their views still had a bearing on modern scientific problems.

Raymond Pearl acted as Greenwood's host and guide to the American scene and soon introduced him to HL Mencken, one of Baltimore's literary celebrities, whose philosophy ran in somewhat the same grooves as that of Bernard Shaw. He took him through the New England countryside and his guest was delighted by what he saw, One of Greenwood's oddest meetings was with a certain Mr. X, of Jewish / German descent who had once been a friend and neighbour at Loughton. In the years before the 1914 war Greenwood's wife had naturally sought out anyone in Loughton of German origin and a close friendship had developed with Mr. X's family.

Then came the war and when Mr. X applied to join the Local Defence Force he had been unnecessarily humiliated on account of his German origins by the Local Officer in Command, a General Practitioner who tended to specialise among the more prosperous local residents. After the war Mr. X emigrated to the United States. "What will you do there?" Greenwood had asked when X came round to say farewell. "Oh, well," said X, "I know about foreign exchange. I daresay I shall be able to get a job." He did. When Greenwood eventually tracked him down, Mr. X had turned into a banking tycoon, and whether or not he ranked in the millionaire class, instead of a rather shabby old farm house owner on the outskirts of Loughton, he now dwelt in the midst of a large estate, isolated from the workaday world by a secretariat that would have done credit to a cabinet minister.

It chanced that some years previously a young man only slightly known to Greenwood had asked for his letter of recommendation on emigrating to the States. Greenwood, knowing nobody in the commercial world, which was what the young man had intended to enter, gave him a letter of introduction to Mr. X, supposing that he at least would have some business contacts. When they met, Mr. X recalled the incident and said a little anxiously, "I made the chap head of our Western Branch. I hope that was all right?

Greenwood returned to England in the middle of December having thoroughly enjoyed his adventure and with a much higher regard for Americans than when he set out. He

received his fee for the lectures at a time when the American dollar was at a premium and benefitted considerably from the rate of exchange. He sent his wife and elder son on a lush cruise in the Mediterranean and then with a characteristic streak of conscious stricken generosity decided to use the balance of the money to subsidise the holidays of his staff who did not have the advantage of being paid to give lectures abroad during their normal working hours.

9.2. Academic Assistance Council

In May 1933 Greenwood became one of the Foundation Sponsors of the Academic Assistance Council. He agreed to become its Honorary Treasurer and retained this position until his death.

The Academic Assistance Council (later the Society for the Protection of Science and Learning) was largely the idea of Sir William, later Lord Beveridge, the Director of the London School of Economics, and a dynamo of social ideas.

The Nazis had recently risen to power in Germany and were beginning to dismiss from their posts, university teachers because of their Jewish origin or political beliefs. Beveridge conceived the notion of a Society to give help to such people who wished to leave Germany and continue their academic work elsewhere. He put the idea to the governing body of the London School of Economics and reminded it that in addition to helping scholars, here was an opportunity for recruiting first class talent from abroad. The scheme was enlarged and a provisional committee formed with Lord Rutherford as Chairman, Beveridge and Prof CS Gibson as Secretaries and Greenwood as Treasurer. The Royal Society willingly gave its support and in May a Manifesto was published in all the main newspapers explaining the objects of the Academic Assistance Council and inviting funds and support. The Manifesto was signed by forty three sponsors, all eminent men of learning. Their names ring a roll call of the period: William Beveridge, WH Bragg, Lord Backmaster, Lord Cecil, JS Haldane, F Gowland Hopkins, AE Housman, JM Keynes, Lord Lytton, Gilbert Murray, Lord Rayleigh, Lord Rutherford, CS Sherringham, JC Stamp, JJ Thompson, GM Trevelyan, … and Greenwood was among them.

How he first became involved is not clear, but he had three strong connections with the sponsors at large; the Royal Society, the Statistical Society and the professorial body. He and Beveridge were both Fellows of the Statistical Society and had been elected in the same year, 1909, while his position as Foreign Secretary to the Society gave him connections with foreign academic bodies, and his election as next President made him representative of his science as a whole. Moreover his antipathy to the Nazis and his sympathy for the Jews were well known.

The newly formed Council found it easier to secure sympathy than funds, and began its existence with a series of appeals and public lectures. Albert Einstein, who had already left Germany, began the lectures at the Albert Hall in October, where a vast number of people had attended. In the series of lectures that followed in various places, given by men ranging from Lord Rutherford to Sir Austen Chamberlain, Greenwood spoke on "Academic Freedom and the Nazi Revolution" in the Conway Hall on January 2nd 1934. "About 100 to 150 present", he wrote in his diary. "FG Gould of Loughton, in the Chair. Audience interested and a quite interesting discussion …. Gave a supper party (Culpin, Joe, Isserlis) at Schmidts. To bed at 12.30."

The subsequent affairs of the Council have been told by Lord Beveridge himself in his book, *A Defence of Free Learning* (OUP, 1959) in which he paid tribute to Greenwood's work. Several times, it appears, Greenwood had "modestly tried … not to be our Honorary Treasurer, arguing that we needed a big business man or figurehead, but had in fact served us in this laborious post almost from the beginning. No one could have done the work better."

In 1936 the Council was reformed as the "Society for the Protection of Science and Learning" and a trust fund established to support its activities. These became more extensive as political persecution spread over Europe, from the Spanish Civil War and the Dictatorship in Portugal, to the Nazi purges in Germany, the Fascist discrimination in Italy and the victimisations that went on in Russia. Then came the holocaust of the war,

and later the upheavals caused by the Russian occupation of the Baltic countries and Hungary. It is a tragic reflection on "progress" that the twentieth century persecutions of learning in Europe were as cruel and bigoted as those of the middle ages. The Society still continues its work.

9.3. Karl Pearson and the Guy Medal in Gold

In 1933, anticipating their centenary year, the Royal Statistical Society wished to award their Guy Medal in Gold to the most distinguished living statistician, Karl Pearson. This was a delicate matter. KP had always held himself aloof from the Society. "I do hope the old man won't be cantankerous," wrote Yule when he heard about it. But the old man was cantankerous.

6th December 1933

"My dear Professor," wrote Greenwood, as one of the Society's secretaries,

"The Royal Statistical Society from time to time awards medals called Guy medals (after WA Guy), a not very good but very enthusiastic medical statistician who was an officer of the Society for many years and left us a large bequest). The medal in silver is a more or less routine award but the medal in gold is only rarely awarded. In the 25 years I have been connected with the Society the recipients have been, I think, Edgeworth, Udny Yule, Stevenson and Flux. There is a very general desire on the Council, that in this centenary year you would honour us by accepting a medal, but it is naturally not desired to go through the various formalities if the suggestion were in any way unwelcome. It is usual to make the presentation at one of the meetings and it would probably be desired to choose the actual centenary meeting at which the Prince of Wales as Honorary President will take the Chair. That, however, is a matter of detail. What do you feel about it?

Very sincerely yours,

Major Greenwood."

Pearson declined in the most gracious and philosophical terms, but he can hardly have viewed the award of the gold medal to Yule, his junior in statistics and one time assistant, as much of a recommendation. Yule, in fact, had received the medal as long ago as 1911. His refusal, he said, was certainly not through any want of gratitude and he thanked the Society for the honour it wished to pay him. But when he had started his career in statistics about 1889 the Society had occupied a field that was most distasteful to him and he had accordingly stood aside. Since then the attitude of the Society had changed, but this change was due not to him, who had played no part in its affairs, but to its younger members. To them the honour should be paid. Medals, he said, were a great encouragement to young men. When old, one wanted no encouragement and went on because it had become a habit.

Greenwood replied on December 11th:

"My dear Professor,

I shall, of course, try to express your wishes. If it would be in any way objectionable to you as a matter of principle, that is an end of the business. I think, indeed I know, that to give you even a small pleasure is the primary motive. A secondary motive is no doubt that which – if my memory is not at fault – led the French Academy to put up a bust of Moliere in their meeting room with the inscription "Rien ne manqué a sa gloire, il manqué a la notre". In a way the RSS does represent "statistics" officially and it is a blot on its record that the greatest living statistician is not a member (a defect which we are unable to remedy by electing him an honorary fellow, because under the bye laws no person resident in Great Britain can be elected an honorary fellow). So, no doubt, that motive has some influence, but only a secondary influence.

What you say about "honours" is my faith, but I suppose there is something to be said on the other side. In a "deferential" country – as Walter Bagehot once called us – honours do practically always come to people who are not in the least likely, when they come, to be thereby stimulated to further efforts. But I suppose the imagination of some young people

is stimulated by the dream of *one day* becoming Lord Chancellor or even President of the Royal Society. I don't think a renunciation of "honours" by the rarest spirits of the age will really lead to the conferring of them upon young, worthy men. It will only mean that instead of wise, elderly men receiving some proportion, dull and vain elderly men will take the lot and generations will pass before the whole world sees what nonsense it is.

Ever yours,

Major Greenwood"

There is some evidence in the Yule correspondence that Greenwood had put out feelers to get a Government Honour awarded to Pearson on his retirement from the Chair of Eugenics at University College in 1933 and that when this failed, or more probably was scotched by KP himself, the possibility of the Guy Medal came to mind. KP remained the Great Commoner to the end.

9.4. Diarist

In 1934 Greenwood started to keep a diary which he continued on and off until his death. It was mainly a brief affair, listing his main appointments for the day, the things he did, people he saw and books he was reading. There were relatively few comments on people or things. Much of it was written last thing at night, almost as a chore, when he was tired and in no condition to make profound or amusing comments on anything. Humour never breaks in; unkind comment about others, rarely; self congratulation sometimes ("lectured, rather well"); self-pity, frequently towards the end ("depressed, gloomy", etc) …. Yet for all their scantiness these diaries are at times amusingly self-revealing.

> 7/1/34 "Haircut; vexed with myself for giving too small a tip; 2d instead of 3d."
> 6/7/34 "Dined with Isserlis at Schmidt's. Bill 7/2. Gave waiter a tip 2/- and was dreadfully annoyed afterwards by my absurd ostentation. 1/- would have been enough. I suppose I have a streak of miserliness because I am so annoyed by careless generosity."

Among the few subjects that animated him during his diary writings were Air Raids during the war, and snug little sessions of the Statistical Society Dining Club. He often listed the guests and noted down the wines he had at these and other parties. Volnay …. Port 1908 …. Madeira 1884 …. The Port absolute rank poison …. Drank more Burgundy than usual …. Latour 1920 …. Port 1878 …. Taylor's Port 1896 …. A miserable port but a good brandy. Of a dinner, No Wine, a little dull. For all this, he was a very spare drinker, and although he occasionally recorded headaches, no-one ever remembers seeing him even uninhibited through alcohol.

Food he seldom detailed, except during the war years when academic affairs were sometimes, to his dismay, discussed over sandwiches. He never recorded conversations, which shows he was no diarist at heart.

9.5. Nutrition Controversy

Early in January 1934 he got involved in a controversy in the *Times*. Unemployment stood at about 2 and one quarter million. The government was concerned about the ability of the unemployed to feed themselves adequately. The Ministry of Health, taking the opinion of the Medical Research Council estimated that a man required 3095 calories a day. The Medical Research Council's figure had been derived from a Committee finding over which Greenwood had been Chairman. This figure was given press publicity. On January 6th the *Times* published a comment by a committee of "experts" of the British Medical Association which argued that 3400 calories were the minimum requirement. Mention of "experts" made Greenwood see red. In a letter to the *Times* on the 8th he defended the MRC and attacked the BMA committee in terms that were perhaps rather more bitter than the occasion warranted. On the 9th the BMA "experts" replied. On the 10th Greenwood sharply rebutted them. On the 11th Lord Dawson of Penn intervened with a suggestion that both parties should meet at the Royal College of Physicians, of which he was President. At 11.30 on the same morning the Minister of Health (Dr E Hilton Young) and his First Secretary, Sir Arthur Robinson, sent for Greenwood. The interview lasted for three quarters of an hour. The Minister made it plain that he agreed with Lord Dawson's suggestion. Greenwood was obliged to fall in line, reluctantly, it seems,

because he regarded Dawson's suggestion as a "bleat for the angry experts". He suggested regional nutrition committees and offered to write a memorandum on the subject. The Minister agreed. Nevertheless he liked the Minister and it was pretty clear that the Minister liked Greenwood, as a personal friendship ensued. "Hilton Young flattered my vanity" Greenwood recorded in his diary for the day.

On January 11th he submitted to the First Secretary his *Memorandum on Nutrition Committees*. The Minister liked it, and Greenwood soon found himself caught up in Nutrition Committee work.

Although the affair gained him an influential friend it was on the whole, unsatisfactory publicity. His Committee's calorie requirement figure was based on a scientific minimum. The BMA "experts" were more generous. They believed that men and women required more than the bare minimum. The matter was given press publicity and in some quarters Greenwood was unfavourably criticised and accused of wanting to keep the unemployed at starvation level.

Repercussions followed: Feb 13th "Rude letter from (Sir George) Newman." Greenwood's superior at the Ministry of Health. "I telephoned an equally rude answer." Feb 14th; "Hysterical apology from GN".

9.6. Centenary of the Royal Statistical Society

Inspite of its somewhat nebulous status among the learned societies, the Royal Statistical Society was an extremely august body. Founded in March 1834, its patrons had usually been the reigning monarch and its honorary presidents the heir to the throne.

In 1933 plans had been outlined for the celebration of the Society's Centenary, and among them Greenwood had been nominated to become President. Although officially he did not take office until later in the year, he was heavily involved in the centenary arrangements which were due to begin on April 16th.

The plans were grandiose. University College had agreed to lend the Society its premises for the occasion. The Government had agreed to donate £700 towards expenses and other donations were received. The International Statistical Institute was invited to participate by holding its Annual Meeting in London. Lectures and meetings, an entertainment by the City Corporation, visits for the ladies, and seats for a ballet at the Sadlers Wells Opera House (*Nutcracker Suite*) had to be arranged. The Chancellor of the University, the Earl of Athlone, had agreed to inaugurate the meeting of the International Statistical Institute on the 16th April, and the Prince of Wales, as Honorary President, had agreed to take the Chair at the Centenary Meeting of the Statistical Society on the 17th. As one of the Society's Secretaries and President elect, much of the organisation fell on Greenwood, but there was another willing horse, Granville Edge, who became the Organising Secretary for the occasion.

In March Greenwood wrote the addresses for the Prince of Wales and the Chancellor. Both were recorded in the Society's Journal and were models of their kind. Thereafter, whenever his friends were in difficulty over speech making – and he lived among a very speech-making set – they approached him for help.

Like an actor with stage fright he was gloomy about the celebrations, but when the time came everything went off smoothly. Lord Meston, the retiring President, and the three secretaries met the Prince. He was only five minutes late, which surprised Greenwood who was under the impression that Royalty were always much later than this, though the Earl of Athlone had actually been ten minutes early for his part of the proceedings. After hand-shaking the Prince was led into the Great Hall of University College. Here he read his address in a clear voice and must have been astonished at his gracious erudition and the almost paternal interest his royal ancestors had taken in the affairs of the Society. Soon after he slipped away, but not it seems before Greenwood had passed a few words with him about dogs, the Prince having recently lost a favourite Corgi. Thereafter the Centenary Meeting continued, but "Lord, how dull", was Greenwood's Pepysian comment. Later in the week he contributed a paper in French to the International Institute's deliberations, but on the whole found their meetings rather heavy going.

9.7. President of the Society

In June 1934 he took up his office as President, and on November 20th delivered his Presidential Address on *University Education; its recent history and functions*. It was a subject more in keeping with the earlier traditions of the Society, a general enquiry helped along by a little counting. The hall was nearly full and he was well received. The retiring president and Udny Yule spoke charmingly in response. Afterwards there was a dinner for twenty six, but getting home was terrible. There was fog, the trains were cancelled and only by complicated shifts did he and Isserlis manage to get back to the latter's house at Wanstead where he spent the night. He wrote in his diary, "Very tired at the end of a, for me, memorable day."

He may have reflected with pleasure on the fact that his predecessors in the Presidential Chair had been three Marquises, five Earls, four Viscounts, fifteen Barons, three Baronets, nine Knights, and thirteen commoners, one of whom had been Mr Gladstone. The character of the presidents had changed with the character of the Society. Originally the Presidents had tended to be statesmen and the interests of the Society numerical observations on the affairs of the State likely to be of value to guiding policy. By the 1870s the papers submitted were becoming more technical and the Presidential choice increasingly fell on people who had distinguished themselves in statistical work. By the turn of the century a custom had grown up of alternating an "internal" with an "external" President. The "internal" President would be a statistician, the "external" one some distinguished non-statistician who could keep the Society in touch with the world outside. Greenwood's predecessor had been a highly placed Indian Civil Servant; his successor was an ex-minister of the Crown. As the "external" Presidents seldom knew much about the internal workings of the Society, they necessarily had to depend upon their "internal" predecessors.

9.8. Mice

Much of Greenwood's "home-work" during this period dealt with epidemiology among mice. WWC Topley, Professor of Bacteriology and Immunology had conducted a long

series of experiments with these creatures, infecting them with mild disorders and noting down the epidemiological results. He provided Greenwood with his data. Night after night, week after week, he struggled with the mouse data trying to find patterns of significance. But patterns, if there were any, continued to elude him. “Worked on mice”, ran his diary; “more mice”, “mice all day, puzzling creatures.” Long afterwards one of Topley’s daughters commented that her childhood had been blighted by these experiments. What eventually emerged in 1936 was a joint report by the collaborators, M. Greenwood, A Bradford Hill, WWC Topley and J Wilson (*Experimental Epidemiology*, Medical Research Council *Special Report Series* No. 209)

9.9. Epidemics and Crowd Diseases

In March 1935 his first full size book was published. He sub-titled it, *An Introduction to the Story of Epidemiology* and dedicated it to “The Tea Club” at the School, where some of its themes had been discussed.

The first 127 pages discussed general principles and methods; the remaining 252 dealt with special illustrations with regard to particular diseases. The book was favourably reviewed and within the limits of the subject interest and its relatively high price, it was well received. In a sense it was two books and reflected Greenwood’s dual personality, his mixture of literary and scientific interests. It combined historical and philosophical observations with text book illustrations. He might actually have made money out of the effort if he had written the two parts separately, but the two parts together were never destined to be a money-spinner. Science and literature combine only in the rarer sprits. The average student of epidemiology was as likely to lose patience over a page that quoted Thomas Aquinas, Shakespeare, Galen and Sir Thomas Browne, as a literary reader over a page packed with decimalised statistics and casual references to algebraic methods. The text-book part has since been re-written by men who were not literary minded, some of whom were perhaps the author’s pupils, and in the curious way Time has with books, *Epidemics and Crowd Diseases* has now become quite rare and may in future be prized not for the scientific ideas it propounded, but for its literary and

philosophical comments which were perhaps closer to the author’s heart than the scientific parts.

9.10. Fathers Lopez and Albert

His interest in psychology having been aroused by Millais Culpin and May Smith, Greenwood began dipping into the works of Galen, St. Augustine and Thomas Aquinas. He had long been convinced that the *ideas* of the ancients deserved *sympathic* re-examination, and deplored the popular assumption that because the deductions of the ancient writers were now proved to be wrong, all their work was therefore equally valueless. Because the writings of Galen as a physician were useless as a guide to treating a sore throat, this did not automatically mean that nothing Galen wrote had any bearing on modern medical practice. On psychology he thought the ancient writers might well be illuminating. With Galen he felt reasonably at home, but the theological turn of the Fathers puzzled him. He had already conceived the notion of Epidemics of the Mind. And religious faith seemed to have a bearing on this. To get some practical information about the teachings of the Roman Fathers he hit upon the notion of consulting the local Catholic Priest to whose flock his wife belonged. Hitherto he had maintained a haughty disdain of the Catholic Church. He had never forgiven it for humiliating him in Germany by forcing on him certain conditions before sanctioning his marriage. Now he was not so condemnatory. Perhaps, after all, in the light of old psychological experience, Roman Catholic ritual and dogma did possess some inner significance.

The local Catholic Church was a new foundation with Spanish connections. He invited Father Lopez to supper, and plied him with questions about the doctrines advanced by St. Augustine. The poor man was dumbfounded and confessed complete ignorance on the subject. Greenwood’s views on the Catholic hierarchy were reinforced, but soon Father Lopez vanished away and Father Albert took his place. Father Albert knew little more about St. Augustine than his predecessor, but he was an entertaining talker. Increasingly he was invited to supper. The 1908 port was opened for his benefit; bottles of wine were given at Christmas, and gradually it seems, Greenwood the Protestant gave Father Albert the priest a first class grounding in Catholic doctrine. No wonder Yule commented a little

later, “Like you, I have found increasing age leads to a steadily growing sense of religion.”

One thing continued to puzzle Greenwood. How was it possible for Father Albert to have lived for twenty five years in England and yet still speak the language so badly?

9.11. The Senate

In March 1934 Sir Ernest Graham Little, MP for the University of London, and a member since 1906, persuaded Greenwood to have his name put forward for candidature to the University Senate. The proposal seems originally to have been inspired by Graham Little’s wife, who was very industrious in the way of these things, and may have been partly responsible for the enormous number of official appointments her husband acquired during his long life. Graham Little was at bottom a physician and dermatologist with a complicated overlay of politics and literary interests. Greenwood was summoned to the Graham Little house, and Lady Graham Little lectured him for the better part of an hour on University politics and the way the handle elections. Soon after he was tipped off that his candidature would be opposed. He was angry and nearly withdrew. In May, however, he was elected and drank some of the 1908 port in celebration. But he was soon disabused of the honour. The wordy tedium of Senate business bored him. By December he was writing in his diary, “The Senate is no bloody good” and a year later, “Fed up with University business.”

9.12. A Dream

February 10th, 1935; “Curious dream: That I fainted while lecturing and sank behind the desk without anybody coming to pick me up. The curious point is that I cannot identify the place ….”

9.13. Death of Karl Pearson

Greenwood started 1936 by investigating the changing nature of death rates. Meanwhile there was a series of more personal mortality. In January King George V died and the Honorary President of the Statistical Society ascended the throne. In February

Greenwood's uncle Ernest Burchell died, his mother's brother, a general practitioner at Brighton and a long accustomed Christmas guest. In March the Society's old rooms in the Adelphi were condemned to destruction, and almost in sympathy with the Society from which he had always stayed aloof in life, Karl Pearson died in April. On the 29th of that Month Greenwood commented wistfully in his diary, "My father's birthday. He would have been 82. I have at least succeeded to the extent that he would have been proud of me, *Eheu fugaces …*" a year ago on the same date he had commented, "I was often rude to him in the 20s but perhaps on the whole was not too bad a son." In May his father in law, Andreas Baur, died, and in November Alice Bacot, his last link with Arthur Bacot, passed away. In December, Edward VIII now Patron of the Society, abdicated. "Listened to the Archbishop of Canterbury's disgusting broadcast, "he wrote in his diary on the 13th. "Edward may have been a beast but only a cowardly knave insults the man who is down …."

The first he heard of Pearson's death was on April 27th at 6.10pm when the *Times* rang up and asked for an Obituary. He set to and phoned it through at 10pm. "*Ultimus Romanorum mortus est*" he commented. "Only one of my heroes still lives, Leonard Hill." The funeral was at Golders Green on the 30th. "Some music. Chambers read part of the Grammarians Funeral and a dull passage from George Eliot. A not very moving ceremony, but I daresay the old boy would have approved. Salut: Odd that his last letter should be printed in the *Times* today. He was a very great man." Greenwood eventually wrote Pearson's life in the *Dictionary of National Biography*. The old quarrels and cold wars were long forgotten, but Pearson was admired rather than loved; he was one of the most influential teachers of his time. Pearson's old angers, Yule reminded Greenwood, were only caused by intellectual things. Mistakes and accidents of ordinary life never bothered the Master.

9.14. The Medical Dictator

This was Greenwood's second sizeable book, a volume of seven biographical essays on Galen, John Friend, PM Latham, William Farr, PCA Louis, William Osler, and AW Bacot. Several of them were expansions of essays published earlier. Dictators were much

in the public mind just then, and the title was based on Galen's Dictatorship of Medicine that lasted a good deal longer than Hitler's boasted Thousand Year State. The essays illustrate all the merits and all the faults, if we may be permitted so to call them, of his earlier book. As literary essays they were excellent, and deserved a wider public than their presentation under a kind of medical umbrella was very likely to get for them. Reading them in conjunction with his earlier book one cannot escape the impression that the author's heart was in literature rather than science, and that if his father had permitted him to study history instead of medicine, his career would have been none the less successful.

9.15. Lord Kennet of the Dene

In June 1936 Greenwood relinquished the Presidential Chair of the Statistical Society and was succeeded by Edward Hilton Young, created Lord Kennet of the Dene on giving up his post of Minister of Health in 1935. After Kennet's Presidential Address in November, Greenwood took the Chair for the last time at the Society's Dining Club. He got home at midnight and wrote in his diary, "So that's another milestone passed. I like Kennet and think he likes me. May Smith says he should have been a cardinal. Well, if there's another smash (war?) he will be a minister again and I shall be his secretary."

Kennet never did become a Minister again but a desultory kind of intimacy developed between the two that lasted for some years until a difference of opinion drove them apart – or more likely a fancied slight caused Greenwood to withdraw.

Kennet, a year older than Greenwood, was the son of a baronet, educated at Eton and Cambridge and originally a barrister by training. He married Scott of the Antarctic's widow, drifted into politics as an MP and became involved in some extraordinary adventures in the 1914 war. Thereafter he was regarded by the Establishment as competent to fill almost any official post where difficulties were likely to occur. His appointments were many, important, and except to the English mind, often seemingly random. His particular expertise was finance, but he handled Iron Ore Enquiries, the Constitution of the University of London, Free Places at Schools, the Gas Federation, the

British Medical Association, the Royal Statistical Society, Indian Currency, the Poetry Society and Bird-watching with the same ease and competence that he had displayed as Financial Secretary to the Treasury.

Anyone with affairs as diverse as this must rely on others and there seems little doubt that at the start of their friendship Kennet found Greenwood "useful". "I like your programme for the House of Lords," he wrote in 1935, "but how can I provide the knowledge that it needs? You must coach me." During his two years as President of the Society he continually sought Greenwood's advice on Society affairs, and their friendship became a good deal closer. Occasionally when some big issue was pending, they went off to Kennet's house at Fritton on the Suffolk border to talk things over. 2nd January 1937; "We dined alone. His study book-lined, mostly books I knew and liked," wrote Greenwood. "Bed early. A small comfortable room." Next morning they talked while Kennet dusted his books. Then they walked through some woods. "Kennet saw a bird like a bull finch which he identified as a brambling and cried out in ecstasy, "You dear". After lunch we went a mile or two in the car, then walked over a marsh down to the Waveney and then to Burgh Castel. A noble sight under the cloud strewn moon … After dinner we sat talking, he still working, until 11.15."

19.16. Convocation

On May 11th 1937 Greenwood was elected Vice Chairman of University Convocation. "Normally the vice- chairmanship of convocation is a perfect sinecure," he wrote to Jo Culpin, Millais' daughter in May the following year. "This year it has made me, and several others, laugh more than any number of *Punch* could make us. The Chairman of Convocation is an aged man named Loney, completely ga-ga. He did not actually have Queen Victoria as a pupil, that is an exaggeration but well that sort of thing. His annual moments of triumph are on Presentation Day, when he walks in procession with a mace bearer and sits on the left hand of the Earl and repeats the performance at the Abbey. His doctor informed the old man that he must not do both so he decided to go to the Abbey and not to the Albert Hall. The Vice Chancellor then ruled that I must take his place at the Albert Hall. The old boy went into ecstasies of rage, swore it was a violation of the

statues, locked up the official robes (which are the property of the University) and refused to surrender the key.

The Vice Chancellor (also a choleric man) insisted on his ruling; keys were tried (in vain) on the official box and in the end I had the mace bearer (and the Earl) but was clad in my own doctor's robes. I need not say that there was a joy among the administrative staff …."

Greenwood was now 57 and a hint of weariness creeps into his diaries. The political situation in Germany was beginning to depress him. Nevertheless he and Rosa went to Germany for their holidays and he was glad to find that his German relatives shared his dislike of Hitler. They were back by the end of August.

19.17. Fit of Terror

August 28th: 1937 "While listening to wireless had a fit of panic terror always associated with the bird headed man of some picture I saw as a child of 7 or 8 on the way to Miss Wright's school. A really horrible sensation."

19.18. Overwork

By the end of 1937 he began to develop signs of minor heart trouble and determined to lay aside some of the work he had accumulated. This was indeed, quite formidable. Apart from his own teaching and research activities, he sat on or presided over a large number of official and non official committees connected with the University, the School, the Royal and Statistical Societies, the Academic Assistance Council, the Ministry of Health, the Medical Research Council, and the Royal College of Physicians. He was doing a good deal of semi-professional journalism for the Medical Press as well as reviewing books. Moreover he was widely consulted over a variety of matters ranging from helping lame dogs over stiles, and dogs that were not so lame, to getting advice to the future Lord Woolton on Labour Wastage and Industrial Training. He was lecturing on behalf of such divers bodies as the Board of Education, the Oxford and Cambridge Hospitals, the

Socialist Medical Association, and the London Natural History Society. Besides this he was conducting a massive correspondence. As if this were not enough he was reading a good deal, including the works of Tacitus in the original, and playing chess whenever he could find a partner.

19.19. Winston Churchill

On April 23rd 1938 he noted in his diary that he had received a letter from Winston Churchill who was his local Member of Parliament. As an intellectual radical Greenwood, had no use for the future Prime Minister. On January 31st 1935 he had commented in his diary, "Listened to Winston Churchill (on the radio) talking rubbish". With hindsight it is curious to recollect how many other people had agreed with him. But times were changing. Greenwood's connections with the Academic Assistance Council brought him news that convinced him that Hitler's excesses would have at some time to be stopped, by war if necessary. Later on Greenwood grudgingly admitted a growing respect for the man, grudging, perhaps, because it admitted that his earlier judgment might have been at fault. The letter referred to above was a sequel to Greenwood passing on to Churchill some information about the inadequacies of the local Territorial Army administration in the event of war, which he had received via his son who was a TA Officer. Churchill, like so many others, mistook Greenwood's Christian name and replied:

"Dear Major Greenwood,
I am very much obliged to you for your letter, and its most helpful suggestions. I wish I had some power to see that effect were given to it. I am much obliged to you for also sending your …. notes. This is most discreditable to the authorities. If I make any use of this information I shall be careful to conceal its origin. I continue to try to do my best, but you can see for yourself how futile the warnings in the past have been, and the humiliations are we have to put up with. There is worse to come in the future.

Yours, etc,

Winston Churchill"

Greenwood's information was by no means shelved. Together with notes from other sources Churchill managed to use it to stir up a first class Parliamentary row...

19.20. Bisset Hawkins Medal

In 1938 the Royal College of Physicians awarded him the Bisset Hawkins Medal, a splendid gold piece weighing nearly half a pound. According to the Trust this was to be bestowed on "some duly qualified medical practitioner, who is a British subject, and who has, during the preceding ten years done such work in advancing Sanitary Science, or in promoting Public Health, as, in the opinion of the College, deserves special recognition. The award was founded in 1896.

19.21. In Imitation of Alexander Pope

His involvement with the Academic Assistance Council not only made him increasingly aware of the growing persecution of German academics but occasionally brought some of them to his very door. From time to time he invited refugees to his house and there they stayed sometimes for weeks until they could find a lodgement elsewhere. In August 1938 he made up some verses on the theme of Germany in the metre of Pope to see how long it would take him. The following lines occupied a little more than two hours to compose. "I have none of my father's facility" he commented:

> Time *was,* in German towns by night and day,
> Ingenious youths were searching out the way,
> The paths which led through learning arid waste
> Not quickly traversed, calling not for haste;
> But patient labour, rooting briars out
> Which choken the way, entangling it in doubt.
> They laboured well and earned the Roman's praise
> Who sang of heroes that in far off days
> Did seek to free the soul of man from dread
> By reason only, reason making head

Against a phantom host of panic fears
Which plunged mankind in horror and in tears.

This is, in German towns by day and night,
And we may see another sadder sight.
Gelehrter now is but a word of shame,
The path they cleared, the briars choke again.

Once more a phantom fear bred out of hate
Has seized mankind and, master of men's fate
The braggarts crowned, the scholars thrust apart,
Enforced to eat his words and break his heart,
And learning, once the German's hope and pride,
By fools derided, now through knaves has died,

This poetic vein was revived in January the following year when Yule composed a statistical song in Latin for the Dining Club's Centenary dinner and asked Greenwood to render it into English. The first verse ran:

Happy Statisticians we,
Who love three things and only three,
Brains of steel to do our work,
Papers which we never shirk
(Unless the algebra's too great)
And Port of vintage 1908.

Not satisfied he revised it;

The statistician's joys are three,
Brains of steel to set him free
From sums, then meeting not too late,

Last, Port of vintage 1908.

("Brains of steel" – a calculating machine. Yule's Latin original ran "machinae mirabilis dictae computoria").

10. War Again (1938 – 1945)

10.1. War Scare

"4th September 1938: The international situation is horribly disconcerting. Impossible not to feel low spirited. 5th and 6th: Depressed: 12th; Heard Hitler's speech at 7.15. I understood it very badly …. 21st; I feel a cowardly relief that there is likely *not* to be a war and a deep sense of national shame. 22nd: Worked all morning on War Plan for my department. 24th: International situation worse. 27th: John (his elder son) called up. 28th: News of possibility of peace. 30th: War for the time averted. I have a feeling of cowardly relief …. Lunched with the Graham Little's who had a domestic celebration of peace ….. Academic Assistance Council at Royal Society. Odd to see the empty house of the RS. Pictures taken down from the walls …. 31st Dec: So ends a beastly year.

Nevertheless war seemed inevitable. In February 1939 he was co-opted onto a Ministry of Labour "Mathematics" Committee in connection with manpower and registration, under the Chairmanship of Charles Darwin, the great Charles' grandson. In March Cambridge University offered to house the School of Hygiene in the event of war making London untenable. At the time it was widely believed that within an hour or two of the outbreak of war, the city would be showered with bombs. Greenwood's depression returned and he and Rosa made each other worse, she having a notion that being German born she would be molested in the event of war. He seriously thought of migrating to the United States and sounded Raymond Pearl, his Baltimore friend. Pearl discouraged him, and his thoughts turned vaguely towards the mountains of Wales.

In the middle of all this his dog, Derby, became ill. While he was away on holiday in Dorset word reached him that Derby was worse and that it would be necessary to put him

to sleep. “I consented with a heavy heart” he wrote. The affair cast a blight over his holiday. On his return home,”Very depressed. Wept over Derby’s grave.”

10.2. Outbreak of War

As the international news grew worse his gloom deepened. August 24th: Very gloomy, think too much of the future; 25th: Very gloomy; bathed, went to town, perhaps for the last time; 26th: Still depressed; D’s guest has appendicitis.”

The last entry above had a tragic outcome that overshadowed the actual break with Germany. His son had been called up and his daughter in law had staying with her a young foreign girl. She became ill almost as soon as she arrived in England and it was discovered that she had contracted typhoid while travelling through France. The girl’s mother hastened over from Hungary and barely arrived before her daughter died. This was on September 2nd... War was declared while Greenwood was arranging the funeral, trying to soothe the heartbroken mother and organise some way of getting her back to her own country. According to those who were involved he excelled himself, and by pulling every wire he knew, just managed to get the mother onto the last plane bound for a neutral European country.

It was a shattering experience and yet it temporarily lifted him out of his gloom – to be of assistance to someone who had far greater reason for unhappiness.

What immediately followed the outbreak of war came as an anti-climax. There was no saturation bombing. There were scarcely even any warnings of possible air raids. Nothing violent happened at all. Instead there was a stream of official regulations. As far as Greenwood was concerned the daily round continued as before. He was called on to advise on statistics for the Civil Defence Technical Committee, and feeling a need for more personal involvement, he called on his Local Medical Officer of Health and offered his services if ever they should be required. The year ended with a “rabble” of Ministry of Information staff taking over a portion of the School.

10.3. Diary Extracts: 1940

Jan. 1st: A good day's work. Finished Memo for ARP.

Jan. 2nd: Up to School in own car. May (Smith), Teleky (Agnes E Teleky, one of the refugee academics from Europe), Munro (Sir David Munro, Chief Medical Officer, Ministry of Supply). Tony (Austin Bradford Hill, eventually Greenwood's successor) Pretty busy. Home 4.35. Letters to Graham Little and Mr Fowke (Hilda Woods, formerly on his staff at Hampstead had married a Mr Fowke).

Jan. 3rd: Some work. Drove Rosa to old people (Greenwood's Uncle Frank and Aunt Bessie. They lived in north London and Greenwood used to visit them once a fortnight). Back by 4.30. Worked on lecture and read Corado Gini (Professor Corado Gini, Italian statistician and acquaintance since the 1920s).

Jan. 4th: Pretty good day's work. Lecture.

Jan. 5th: Up to School by public transport. A slow business. Interviewed Fawcett, an external student. Talked to X about Teleky's work. Took her to lunch. Saw Dr Simpson. Home at 5.0. Sad letter from Woods.

Jan. 6th. Pretty good day's work. After lunch (it was a Saturday) fetched a parcel from village for Rosa (For some time his wife had been "poorly" and on doctor's orders was taking things easy). Felt rather ill coming back. NB: After drinking any wine and eating, not to walk uphill for at least 3 hrs (He was becoming increasingly conscious of his heart trouble. He now, for instance, took to walking upstairs very slowly).

Jan. 7th: Horrid fog. Cleaned car. Short walk. Deta and Roger (His daughter in law and her son) here. Baines (a neighbour at Loughton) came in evening.

Jan. 8th: Up to town by car. Took Edge and David Edge. Sent in Minute to CMO (Chief Medical Officer). Saw Mellanby (Secretary to Medical Research Council).

Jan. 9th: Bartlett's paper on Mathematical Statistics (Probably Maurice Stevenson Bartlett, later Professor of Statistics, University College). Mice (The mice data from Topley) Lecture notes. At 6 lectured to ARP. I think successfully.

Jan. 10th: Finished Bartlett's paper. Mice. Drove Rosa to old people. Cold but fine. Wonderful sunset. Lecture notes. Began Mises on Probability. Notes from Petty papers.

Jan. 11th. Up to School in Edge's car. Committee meeting of Appointments Board. Saw Tony Hill. Fry (The Local Medical Officer of Health for Loughton) came to supper.

Jan. 12th: Fair morning's work. Lectured 6-7. Well enough. A little tired. Played some chess (ie. from a book).

Jan. 13th: Some work. Drove Rosa to village.

Jan. 14th: Cleaned car. Little walk. Called on Deta. Roger is poorly.

Jan. 15th: Roger still ill. Drove up in own car. Fair amount of work. Rather gloomy. Still very cold.

So the daily round continued until in May the German Blitzkrieg fell on the Low Countries and France. He was plunged into gloom and misery. Always inclined to over-dramatise his emotions – to himself at any rate, thoughts of suicide kept entering his mind. 11th June: "The suicide fugue keeps coming on. Yet after tea and some successful algebra a change of mood …." 23rd June: "Very depressed except when the children (his daughter in law and grandson) and Nello (a neighbour) were here. Did a little algebra. Perhaps that may keep me alive for a month or two." He was now using Algebra as an escape route. 14th July: "Lugubrious speech by Winston Churchill. I daresay he is right. Please God my life will soon be over. I should wish to die naturally or by violence not self-inflicted."

As before his depression lifted. France and the Low Countries were occupied by the enemy, but otherwise little had changed except the psychological atmosphere. In July he was engaged on the statistics of bombing accuracy.

10.4. Air Raids

In September 1940 the enemy began to bomb London and some of the bombs fell near Loughton. In the second week of September the raids were so heavy that Greenwood and

Rosa were driven to sleeping in makeshift shelters downstairs. By early October the raids, sleeplessness and the general disruption of the daily round had brought his depression on again. 7th October: “I really no longer take any personal interest in the future. I see no reason why we should win or lose the war. I should suppose that a gradual destruction of civilisation in Europe and England is now entirely certain.” This observation shows how divorced he was from the common belief of his countryman. A small number of academics may have thought as he did, but the rank and file of his countrymen, rightly or wrongly, had no such forebodings. 10th Nov.: “I do honestly wish I was dead, but shrink like most people from being blown to pieces and think it disloyal to Rosa to kill myself.” How wrong can the experts' be! The people of beleaguered Britain were healthy as never before. On the 16th November some bombs actually fell on Loughton, demolished Lord Stanmore's house and killed five people. Greenwood put out feelers for a temporary lodgement in Cambridge but withdrew them when he realised how much Rosa disliked the idea. She hated bombs but she hated the idea of leaving home much more.

The bombing eased and Greenwood was distracted from his private miseries by being called on to assist the Local Medical Officer of Health. He began inspecting local Air Raid Shelters from the point of view of hygiene, visited local hospitals, held clinics, did inoculations and resumed signing death certificates after a lapse since 1904!

When the war was a year old he became resigned to the state of things and to distract himself from private worries worked hard at algebra, which he found difficult, and played chess with Vincent Nello, a Loughton neighbour. When the Russians joined the war against Germany he was not very optimistic of their success but contributed in print to the *Labour Monthly News* his praise of their scientific genius and his condemnation of German sterility in his own field of studies. “One of the dogmas devoutly believed by all Englishmen with no knowledge of Science (ie. practically all Englishmen who hold key positions in public life) that German science is far superior to any other science has never seriously been shaken, “he began. At the most one of our “leaders” might go so far as to admit, that since the coming of the Nazis, German science has deteriorated, but, were he

told that in some important branches of science that Germans *never were* superior to the Russians, a smile of magisterial ignorance would pass over his face."

10.5. Diary Extracts: 1942

Feb 1st 1942: (Sunday) Heavy snow. Did not clean car. Self starter out of order. Short walk. Nello for chess.

Feb 2nd: More snow. Difficult journey to town. Started in Green Lane (bus) but this halted at Forest Rd. Walthamstow. Got a lift to Tottenham and arrived at School an hour late for Board. Lunch with May. Home at 4.10.

Feb. 3rd: Shopped and visited Hospital with Toby (his dog). Some algebra. Swept snow. More algebra and arithmetic.

Feb. 4th: Up by Green Line. Some thaw but grew colder by nightfall. Some work. Discovered a gross error in my paper on War Losses. Annoyed with myself.

Feb. 5th: Hospital. Letters. Algebra.

Feb. 6th: Up by Green line. Flu deaths. Home by 2.11 Green Line. Walk. Algebra. No fire watching. Depressed.

Feb. 7th: Algebra. Hospital. Algebra. Not so depressed.

Feb. 8th: (Sunday) Walk with Toby. Called on Miss Waller (An elderly neighbour who lived alone in a house on the edge of the Forest). Nello, chess, tea.

Feb. 9th: Up by Green Line. Board. Lunch with May. Some work. Home at 5.15.

Feb. 10th: Hospital. Up by Green Line. Tuberculosis Committee. Back by 5.15 Green Line. Very crowded.

10.6. Diary Extracts: 1943

March 1st: Up in car. Board. Lectured to Poles (A course on Epidemiology for Polish Army Officers.). Tony called. Practical class. Home 5.10.

March 2nd: Up in Edge's car. Board. Lectured to Poles at 11.30 and again at 2.0 Left for home at 3, tired. Walked with Toby. Pleasant tea.

March 3rd: Up in Edge's car. Lectured to Poles.

March 4th: Up in Edge's car. Lectured to Poles. Meeting Cancer Research Fund. Gye, Dean, Beattie, McNalty (WE Gye, Director Imperial Cancer Research Fund. HR

Dean, Chairman of Council of Imperial Cancer Research Fund; probably John Beattie, Bernhard Baron Research Professor, Royal College of Surgeons; Sir AS MacNalty, member of Council of Imperial Cancer Research Fund).

March 5th: Up by bus. Flu deaths down to 92. Short lecture to Poles. Fire watching.

March 6th: Shopped and hospital. Walk with Toby. John came to supper and seemed well although he still has a cough.

March 7th: (Sunday) Walk with Toby. Miss Waller. Nello, chess & tea. An alert after midnight. Lovely spring day. Saw two little fawns on High Beech side of New Road.

March 8th: Up in Edge's car. Board. Woman from BBC Home 5.5.

March 9th: Up in Edge's car. Board. Home at 5. Irritable. A beautiful day. Finished article for BMJ.

March 10th: Up in own car. Bought a cake. Lectured to Poles. Practical class. Home at 5.5. More cheerful. Weather continues beautiful.

10.7. Fitzpatrick and Linacre Lectures

In February 1943 he had delivered the Fitzpatrick Lectures at the Royal College of Physicians; his subject, *Medical Statistics from Graunt to Farr*. It was not a subject of outstanding interest in the middle of a world war and apart from a few friends and college officials hardly anyone turned up. "A lecture." Far from it. In May he gave the Linacre Lecture at Cambridge where he was astonished to find an audience of nearly 300 people. His subject: *Authority in Medicine: Old and New*. "An invitation from the Master and Fellows of St. College" he began, "to prepare this lecture brought me satisfaction of a kind which a majority of the audience cannot experience. I have known and loved Cambridge in general and St. John's College in particular for many years, but my memories are largely of vacations …. My memories are of weekends and haunt a set of rooms in the Second Court of St. John's where, thirty years ago, a young weekend visitor eagerly listened to his host's account of Cambridge life and, if he did not actually break the 10th Commandment, was certainly more conscious of the advantages than the drawbacks of Collegiate life …."

Thirty years ago! Before the 1914 war, when he and Yule had cycled around East Anglia, and from the crowded uncertainties of the Lister Institute he had envied Yule's appointment and longed for a set of rooms himself in some ancient college court ….

The lecture was over; afterwards there was a Feast at St. John's and he sat between the Master and Yule and they drank Richburg 1923, a port of 1891, and ate soup, steak, asparagus and gooseberry pie, delectable fare in the fourth year of the war.

In September the acting Dean of the School, Colonel GS Parkinson, was released for war work, and reluctantly Greenwood allowed himself to be appointed in his place.

10.8. Diary Extracts 1944

In February the bombing of London started again. On the night of the 23rd the guns were noisier than usual and after a time Greenwood came downstairs. As he did so he heard a number of soft hisses. "Opened the front door," he wrote to his son, "upon a scene like a gigantic theatre staging the end of a Wagner Opera. The sky was alive with shell bursts and searchlights, and the general blazing with the incandescent glare of incendiary bombs. One of them was by the garage. I shouted to mama to come down, expecting the garage to go up in flames, and then smelt burning in the house. It seemed like a finale …. But there were plenty of soldiers who were billeted in the house next door and half a dozen of them rushed over with a stirrup pump. A bomb had come through the roof and was burning on the maid's bed. They soon extinguished it and dragged the smouldering mattress into the garden, where something like ten incendiaries had fallen. A house opposite had 29.

One bomb went through the roof of our oldest chicken house and was arrested by the concrete floor. It burned away within inches of the rotten woodwork and singed the tails of one or two roosting hens. Next day there were six eggs laid, double the average. British hens, you see, can take it."

But there were plenty of intervals when nothing happened to interrupt the old pattern of life;

April 1st: (Sunday) Shopped & hospital. Walk with Toby. Algebra. Report by Blacke; letter to Hutchinson. Quiet night.

April 2nd: Walk with Toby; Deta and Roger to lunch. Nello to chess & tea.

April 3rd: Fair after a quiet night. Up in own car. Algebra. Physiology. Home at 5. Car out of sorts. Some algebra.

April 4th: Up in own car. Board. Algebra. Home at 5.0. David Edge came with his father to say goodbye.

April 5th: Up by bus. Visited (*word omitted*) Dunham in afternoon. Senate meeting at 11.30. Spoke. Home at 5.30. George (younger son) and his fiancée arrived at 10.30. Quiet night.

April 6th: Rested. Walked with Toby. Shopped. Quiet night.

April 7th: Walked with Toby. Read. Rested. Quiet night.

April 8th: Shopped and hospital. Walked with Toby. Read some algebra with success. Cousins to tea. Quiet night.

April 9th: (Sunday) Good walk with Toby. Glorious sight of stars and moon. Quiet night.

April 10th: Walk with Toby. Quiet ….

In June he and Rosa made an excursion to Lancashire to attend the wedding of their younger son, and returned to face a fierce bombing raid on London.

On 23rd June a flying bomb exploded only 150 yards from his car as he was driving through Woodford. Night after night he and Rosa were reduced to sleeping in a Morrison shelter under the dining room table. The strain of this gradually told on Rosa's already weakened health. She had grown accustomed to hiding her fears but they were now beginning to undermine her will to continue.

10.9. Everybody's Political What's What

In summer 1944 Bernard Shaw published his *Everybody's Political What's What*. It contained a good deal of scathing comment on Greenwood's subjects under such titles as *The Collective Statistician*, and *Our attempts at Anthropometry*. Greenwood was not mentioned, but his hero was and much that he stood for, "Pearson, always smiling and charming, would not admit that anyone who was not a mathematician could claim any scientific authority whatsoever. I subscribed faithfully to his journal *Biometrika* without understanding any of its equations or more than, say, 5 per cent of its sentences. But I found that the biometricians, though their technical skill and subtlety seemed wonderful to me, were as credulous, as prejudiced, as thoughtless as to the facts they were measuring and the assumptions from which they started, as Isaac Newton himself. Even their counting was not to be depended on; for they added up facts and opinions indiscriminately, and cooked their calculations by "weighting" them with fancy figures which represented nothing but their personal guesses and tastes."

There was much more in this vein and an attack on the effectiveness of vaccination. The Editor of the *British Medical Journal* asked Greenwood to "pick out some points for criticism …. That would help non-medical as well as medical readers to get the matter in perspective." (*BMJ* Oct 28 1944:ii:570).

It was a difficult assignment to cross swords with one of the greatest wits of the age, whose opinion of the medical profession was perfectly well known since *The Doctor's Dilemma* of 1906 which continued to earn him royalties. Everyone knew that Shaw exaggerated his views and wrote for effect, to shock or amuse his readers into taking notice and it seems a little stuffy that the *BMJ* should have taken the matter so seriously. For Greenwood it was a repetition of the controversy with Sir Almroth Wright thirty years earlier. But times and circumstances had changed. The battle of the statisticians had been won and although Wright – who as a personal friend of Shaw – had at least known something of the medical background, Shaw knew nothing. Greenwood contented his editor with an examination of the vaccination issue and exchanged a few letters with Shaw via *The Times*. At this distance of time one is tempted to think that a better

rejoinder might have been to poke fun at the great man – though perhaps the *BMJ* was not the best place to do it.

10.10. The Guy Medal in Gold

On April 5th 1945 the Council of the Royal Statistical Society unanimously passed the following resolution:

"That a Guy Medal in Gold be awarded to Professor Major Greenwood FRS for his outstanding work in the field of vital statistics and epidemiology: for the many original contributions he has made to the Society's proceedings and for his valuable services to statistical science over many years."

10.11."Victory in Europe Day" Thoughts

On May 8th 1945, Greenwood typed on a single sheet of paper:

"Diaries were so scarce and I was so listless that I had not kept a diary this year; but the end of the European War is an opportunity to begin again. Rosa has stood the anxiety and cold well. I think we shall continue to live on the ground floor for some time (both were now finding the climbing of stairs exhausting), perhaps always, but she enjoys life.

There is an uproarious rejoicing in the village; it is like a Sunday morning, except for flags – my father's White Ensign floats from our attic, many holes in it, not due to my action, but moth – and a queue at the fishmongers.

I feel rather old, but fit to do a little intellectual work still. I have been given the title of Professor Emeritus and the dear old Statistical Society have given me their gold medal. They are kind people. I have contributed nothing to statistical methodology which will be remembered, except what Yule and I did on Accident Proneness and, just possibly, my paper on the measurement of infectiousness (*On the Statistical Measure of Infectiousness; Journal of Hygiene* 1931; XXXI:336). I had enough scientific imagination but not enough mathematical technique and innate ability ever to produce a first rate

piece of statistical algebra; still, I have helped younger and better educated people by interesting them in problems and so am not *wholly* unworthy of the honours I have received and think I have had more than my share. I suppose that if I were offered a knighthood – I see no reason why I should be – it would please me to make Rosa "my lady" and I should accept but there is certainly no "decoration" I covet at all. I should like to go on earning a little money by lecturing and presiding over the MRC Statistical Committee, for we are pretty hard-up with a *gross* income of about £1100 instead of £1700 - £1800. But that does not worry me a great deal (Perhaps because of his East End origins and his "built in" inferiority complex, he was a continual worrier about money. At times as he grew older he spoke as though he were on the edge of penury. In fact he rarely had less than £800 in cash in his current account, moderately substantial investments, owed nobody anything, and died worth rather more than £20,000 in days when the pound was worth a lot more than it is today).

It is going to be a hard world for old people and I clearly perceive that my knowledge and talents, such as they are, have no commercial value. I am a pretty good writer in a rather Victorian way, but a slow writer, so not an earner of money in periodical literature."

11. The Final Years (1945 – 1949)

11.1. Retirement

On September 28th 1945, with a good deal of sadness in his heart, he went up to the School for the last time as Professor on the active list. The farewells, however, were only nominal. He had every intention of continuing his associations there with the honorary title of Professor Emeritus. Then, with exaggerated anxiety about his finances, he secured a part-time consultancy job dealing with medical statistics for the Essex County Council at Chelmsford. After a few weeks the reality of retirement became quite enjoyable. It was rather pleasant going up to the School in a leisurely way, two or three days a week, chatting with his friends and consulting the store of reference books there. He was able to linger rather longer in his old haunts, at the libraries of the Royal Society and the College of Physicians, and the London Library, and he still gave little supper parties after

meetings at Schmidt's in Charlotte Street. At more solemn dining places there was no more 1908 port, but there were plenty of worthwhile substitutes.

He was still in demand as a lecturer, and was particularly pleased in November when he was called on to give a lecture on Social Medicine to the Cambridge University Medical Society. Times however, were changing, and Yule his former host there, was old and ailing and capable only of entertaining him to tea. Mid-week he used to motor across country to Chelmsford to give advice on the county medical statistics, and took the occasion to dine at quaint old inns he had known in his earlier cycling days with Bacot.

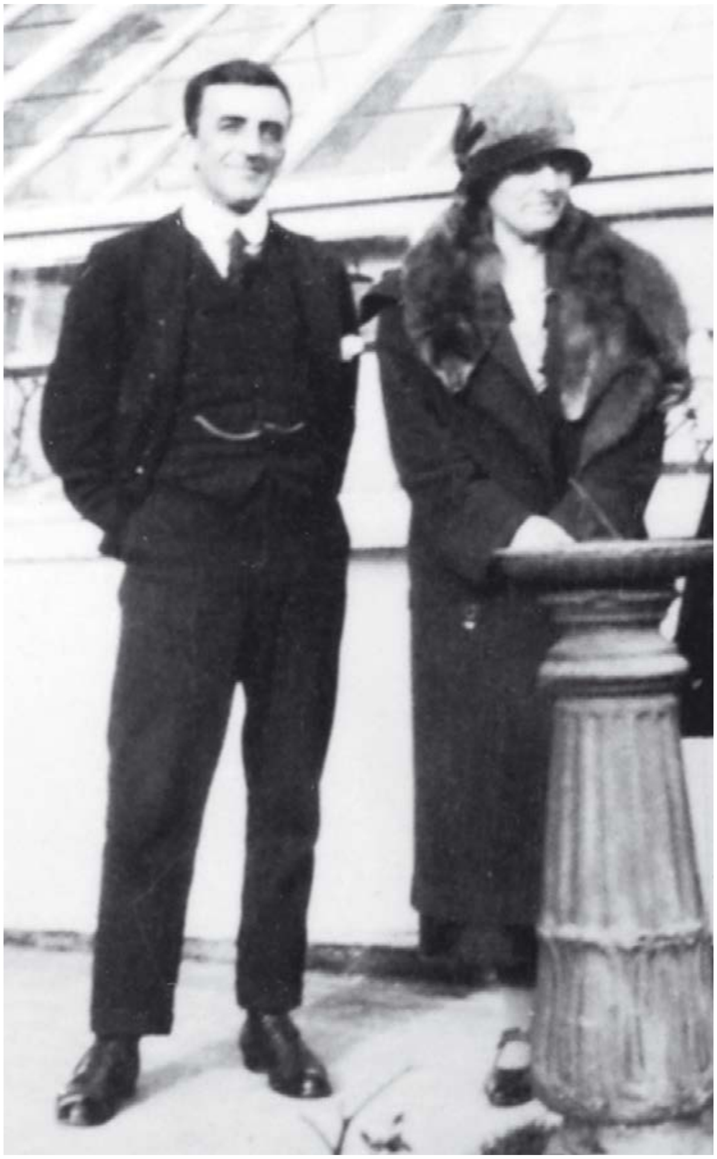

Figure 5: Major and Rosa Greenwood (edited from Farewell, Johnson and Gear, *Journal of the Royal Statistical Society, series* A (2012); 175 (3): 799 - 811)).

Then suddenly he received a blow from which he never completely recovered. On December 13th Rosa died in her sleep beside him. Her death completely unnerved him. For a time the dual parts of his nature were separated. Emotionally he wept for her as a wife and a mother and his grief was unbearable, and the only recourse open to those about him was to steer him into the old escape route of intellectual activity …. There were no diary entries, no written accounts of his feelings, no letters. For a time there was silence until something that seemed like normality returned.

11.2. Diary Extracts 1946

His last years were summed up in the poignant entry in his diary for June 13th 1946. Sitting alone in his study, listening to the silent house, he wrote: “Six months since she died. Oh, my dear, my dear. Why keep a diary?” Rosa had never shared his intellectual interests. She had never been able to hold her own with him in discussions, but she had always been there as a companion, a kind of anchor to reality, and a constant reminder of their youth. “Rosa is not clever”, he once said. “I am clever, but she is wise and I am not.” It was often her wisdom that had turned aside the dangerous cutting edge of his cleverness.

It had been fun acquiring a position and accumulating academic distinctions and pitting his wits against the “experts” of the establishment, but now that Rosa had gone, nothing seemed to matter any longer. There was nobody left to care.

This, however, was an illusion. He had sons and grandchildren, and an enormous range of friends, all of whom would have gone far out of their way to have done him some service.

Yet life went on; visits to the School as an elder statesman, dinners with the Society, visits to his few surviving relatives and his old time neighbours at Loughton. Vincent Nello came in increasingly to play chess. In October he delivered the Heath Clark Lectures at the School, his subject being *Some British Pioneers of Social Medicine*. When they were published he dedicated them to Rosa with a Latin quotation, which she would never have understood.

On 20th December he wrote to his younger son, “I have put your flowers on your mother’s resting place. I visit it every day and say a little prayer; not because I suppose any God there may be is in need of my admonitions, but because it is an emotional relief to do so ….”

11.3. The Letter from Downing Street

On January 2nd 1947 he got a letter from Sir WR Dunstan a friend of long standing, who was astonished at not seeing Greenwood's name in the New Year's Honours List. "Unless you have *refused* a knighthood I am amazed at the omission of your name from the Honours list after all the valuable work you have done. If I have not overlooked your name, then I think the omission scandalous, especially when I remember the sort of honours even mediocrities in the Civil Service get."

Greenwood was ill at the time, heart trouble, and spent the first few weeks of 1947 in bed. In May the "Honours" business came up again. He got a letter from the Prime Minister's office intimating that his name had been forward for a CBE in the forthcoming Birthday Honours List, and asking if such a decoration would be agreeable to him. He replied tersely:

"Sir, I was born in 1880 and am long past by first childhood but not yet, as I think, entered upon second childhood. Consequently the intimation contained in your letter is *not* agreeable to me

Faithfully yours, M Greenwood" (May 15th 1947)

In his diary for the day he simply noted, "Received and declined an offer of CBE (Cheek)." When Isserlis heard about the affair he was indignant. "In offering to "honour" you by a CBE (the Prime Minister) was insulting the whole body of scientific statisticians – and not only the RSS. Your official work for the Ministry and the MRC alone deserves generous recognition. Still, I suppose no government honour would give you as much pleasure now as the Royal (Society) gave you with its medal."

The "honour" was certainly offered in a careless way, but there *were* extenuations. He had continually rebelled against working full time for a government department, and had consistently sneered at titles in circles, where they were held in regard and where influence in the awarding of them was not unknown.

11.4. The Communist Danger

The later part of the year 1947 was to some extent enlivened by a somewhat exotic interest inspired by Sir Ernest Graham Little. Graham Little, who had long been MP for London University was at this period anxious to oppose the influence of Communist infiltrators. The exposure of Alan May Nunn, who had once been a somewhat distant associate of Greenwood's at the University, as a traitor who had been selling scientific secrets to the Russians, had come as a revelation to Graham Little. He saw academic spies lurking in many corners. He asked Greenwood to employ his journalistic skills in exposing the menace. Graham Little's excitement was somewhat naïve and boyish – he was just turning 80 years old – but nevertheless Greenwood did allow himself to become mildly infected and eventually produced a manuscript. What is much more important is that Graham Little roused his interest in the darker aspects of Russian political psychology. He had already been considering the possibility of Epidemics of the Mind in relation to the Nazis and now it seemed, Russian Marxism and its derivatives might provide data well worth studying in this connection. He dipped into dialectical materialism and its curious application to science via the Lysenko controversies. He began reading the works of Hegel, and gradually there emerged, or at least seemed to emerge, some kind of epidemic pattern. He started to work out his ideas on paper. "Within the present generation two psychological epidemics, Fascism and Communism, have devastated Europe and Asia. Each infected group has hated, tortured and killed individuals of the other group with the same ruthlessness as characterised the wars of religion in the 16th and 17th centuries …

Eventually he finished the manuscript of a book entitled *Epidemics of Body and Mind*. He submitted it to a publisher but withdrew the manuscript on the offer of only a 10% royalty. It still remains unpublished.

11.5. Towards Three Score and Ten

Greenwood was not yet 68 and seemed at times surprised that he had survived so long. Various entries in his diary for 1948 suggest that he was curious to know what the signs of senescence really were. Yule was only too ready to provide them. His carcass was steadily wearing out, and he spent much of his time in bed. But Yule was older than

Greenwood, and though his decrepitude was much more apparent, he survived his friend by several years.

The escape route into books and talk and letter writing was still open but the way was becoming a little weary, and when he was not travelling it, Greenwood felt increasingly lonely. He still went up the School three times a week in a leisurely way, and went to Chelmsford about the County's statistical affairs on the other two. He did fewer sums now, and talked and wrote more about the generalities of his subjects. "I have been spending much time," he wrote to his younger son in October, "on a statistical report sent to the Medical Research Council. It suggests to my partly senile mind that although the younger generation of "expert statisticians" know a good deal more algebra than I did at the same age, they have less common sense. They seem to me like the old fashioned schoolmasters who quoted a great deal of Latin when the points could have been made as well or better, in English".

He lectured from time to time and was listened to with awe and a certain amount of incomprehension. Up and coming students asked his advice; former colleagues confided their troubles and asked him to help in their careers; he examined for Doctorate degrees, was asked his opinion on promotions and to support nominations for awards. American Universities asked him to use his influence in persuading English training epidemiologists to teach in the United States. The London Natural History Society made him their Honorary President.

It was all very flattering in a way, but life was becoming emotionally uncomfortable. Many of his old friends had gone into retirement and moved away, or become senile, or died. Leonard Hill, the hero of his youth was still alive, but he was now over 80 and scarcely to be visited. Yule was an invalid, Isserlis was in Dorset, Edge in Wales, Culpin was still accessible at St. Albans and so were one or two others, but usually when he went to stay with his old friends and their families he grew restive and longed to be at home again. There he followed the old routine, forest walks with Toby, the solemn beauty of Monk Wood …. Chess with Nello …. And he went occasionally to church ….

11.6. Diary Extracts 1949

The New Year started badly. On 5th January his dog Toby had to be put away. As usual on these occasions he was miserable for days. "Poor me," he wrote. "No *close* friend left. I *must* work."

On January 19th he participated in a Third Programme BBC broadcast on Voluntary Euthanasia. That he should have agreed to advocate it throws a stark light on his soul. Against him were a woman physician and a Catholic KC. He rested his advocacy on pure reason. They tore it to shreds with human emotion. "I dare say many abusive letters will be directed to me," he had written to Culpin's wife a little earlier, "but I shall not read them." With a sob in his voice he had recalled the necessity of putting his dog Toby to sleep, because he was old and ill. If dogs, why not human beings, seemed to be the implication. One recalls Bernard Shaw's suspicion of the reasoning powers of the biometricians!

As the year wore on he began to imagine difficulties at home. Reluctant to leave *Hillcrest* when Rosa had died, he had asked his elder son and his family to move in. The house was amply big enough, but in post-war terms it was old fashioned, inconvenient and expensive to maintain. Unwilling to modernise and sweep away things that had associations for him, and over anxious about the cost of upkeep, he considered moving into a smaller house and letting his son's family return to their own. Vaguely he built up in his mind an ideal place for retirement, an ancient rectory, perhaps, preferably in East Anglia, with a Victorian library and an old garden surrounded by stately trees, which he could share with a like-minded retiree. He wrote around and soon one or two possibilities turned up. But there was usually something wrong. At one place the *hauteur* of the owner, whose improbable sounding double-barrelled name sent him searching doubtingly into Debrett, put him off. A 14th century manor house with 20th century amenities and a first class housekeeper sounded very attractive, but there was really no hurry, and he determined to look further afield.

He got in touch with a Suffolk parson who had an old rectory. They met and liked each other. For a time it looked as though he really would leave *Hillcrest*. But the pull of old associations was still strong. *Hillcrest* high on the hill, was full of memories, the country house of his youth, still in a wistful way as marvellous as when he had seen it on moonlit nights cycling homewards from Loughton station. It was still haunted by the ghosts of the past, by Rosa, and the children when they were younger, and Arthur Bacot, and the Culpins and the rest. It took little imagination to lie in bed and hear the tinkling of the bicycle bells and the barking of the dogs on Sunday mornings. The church bells would clash out, and there would be the walk in Monk Wood, and Rosa at lunch asking whom they had met there …. It was a hard decision, to leave all this.

But the decision never had to be made. His son became ill. He put aside his plans. Then on September 6th 1949 his old friend and neighbour Vincent Nello died in his sleep, surrounded by his books with the bedside lamp still on. "A very good way to end," he commented and fell to wondering when his own term would come. Once he admired the last exit of Sanderson, headmaster of Oundle School, who, having made a speech on some ceremonial occasion sat down to applause and died.

Within a month he added his own variation to this gloomy theme. On the evening of October 5th he attended a meeting of the Cancer Research Committee at St. George's Hospital. He was invited to take the Chair of a Statistical Sub-Committee, rose to accept, and fell back. This time, unlike his dream of so long ago, people rushed to pick him up. But he was dead, surrounded by doctors!

He was buried with Rosa in St. John's Cemetery at Loughton. When his effects were being tidied up his grandson, aged 13, came upon the diary lying on his desk, and under the date October 5th, in tiny characters wonderingly wrote, "The owner of this book died today."
